# Normalization for Relative Quantification of mRNA and microRNA in Soybean Exposed to Various Abiotic Stresses

**DOI:** 10.1371/journal.pone.0155606

**Published:** 2016-05-13

**Authors:** Weican Liu, Yu Deng, Yonggang Zhou, Huan Chen, Yuanyuan Dong, Nan Wang, Xiaowei Li, Aysha Jameel, He Yang, Min Zhang, Kai Chen, Fawei Wang, Haiyan Li

**Affiliations:** College of Life Sciences, Engineering Research Center of the Chinese Ministry of Education for Bioreactor and Pharmaceutical Development, Jilin Agricultural University, Changchun, Jilin, 130118, China; Pennsylvania State University, UNITED STATES

## Abstract

Plant microRNAs are small non-coding, endogenic RNA molecule (containing 20–24 nucleotides) produced from miRNA precursors (pri-miRNA and pre-miRNA). Evidence suggests that up and down regulation of the miRNA targets the mRNA genes involved in resistance against biotic and abiotic stresses. Reverse transcription quantitative real-time polymerase chain reaction (RT-qPCR) is a powerful technique to analyze variations in mRNA levels. Normalizing the data using reference genes is essential for the analysis of reliable RT-qPCR data. In this study, two groups of candidate reference mRNAs and miRNAs in soybean leaves and roots treated with various abiotic stresses (PEG-simulated drought, salinity, alkalinity, salinity+alkalinity, and abscisic acid) were analyzed by RT-qPCR. We analyzed the most appropriate reference mRNA/miRNAs using the geNorm, NormFinder, and BestKeeper algorithms. According to the results, *Act* and *EF1b* were the most suitable reference mRNAs in leaf and root samples, for mRNA and miRNA precursor data normalization. The most suitable reference miRNAs found in leaf and root samples were 166a and 167a for mature miRNA data normalization. Hence the best combinations of reference mRNAs for mRNA and miRNA precursor data normalization were *EF1a* + *Act* or *EF1b* + *Act* in leaf samples, and *EF1a* + *EF1b* or *60s* + *EF1b* in root samples. For mature miRNA data normalization, the most suitable combinations of reference miRNAs were 166a *+* 167d in leaf samples, and 171a + 156a or 167a + 171a in root samples. We identified potential reference mRNA/miRNAs for accurate RT-qPCR data normalization for mature miRNA, miRNA precursors, and their targeted mRNAs. Our results promote miRNA-based studies on soybean plants exposed to abiotic stress conditions.

## Introduction

MicroRNAs are endogenous and non-coding small RNAs that are generally found in plants. The mature miRNAs are approximately 20–24 nucleotides and are derived from miRNA precursors (i.e., pre-miRNA and pri-transcripts) by Dicer-like enzymatic digestion. The miRNAs play an important regulatory role at the post-transcriptional level by targeting mRNA cleavage or translation repression through the RNA-induced silencing complex, where they form complexes with mRNA[1]. Under stress conditions, plant miRNAs are up- or down-regulated by targeting specific stress-associated mRNAs to increase tolerance to adverse environmental conditions[2–4]. Identification and characterization of miRNAs, miRNA precursors, and their targeted mRNAs are essential for the analysis of the miRNA molecular regulatory mechanism.

RT-qPCR is considered the gold standard for the quantification of mRNA expression because of its accuracy, sensitivity, specificity, reproducibility, and robustness[5–7]. However, because miRNAs are small, RT-qPCR for miRNA detection is more complicated than traditional RT-qPCR methods for mRNA detection. To address this problem, several RT-qPCR-based procedures have been developed to detect miRNA. First, Schmittgen et al. [8, 9] recommended using the traditional RT-qPCR method to monitor the expression of miRNA precursors, including pri-miRNA and pre-miRNA containing the hairpin sequence. The RNA template was reverse transcribed to cDNA using either gene-specific primers or random hexamers and reverse transcriptase. To quantify samples, sense and antisense primers were designed based on the hairpin sequence of miRNA precursors. The pri-miRNA and pre-miRNA templates were simultaneously amplified. Second, Poly(A)-RT-qPCR[10] were developed to monitor mature miRNAs, in which the RNA templates, including miRNAs, were polyadenylated by polymerase and then reverse transcribed to cDNAs using poly(T) adapters. The designed miRNA-specific forward primer and the universal reverse primer complementary to the poly(T) adapter were used for RT-qPCR. The third method involved stem-loop RT-qPCR[9,11–14]. The stem-loop reverse primers were designed with a universal backbone sequence to form a stem-loop structure due to the complementarity between the 5′ and 3′ ends. The specificity of the stem-loop reverse primers for an individual miRNA were based on the fact the last six nucleotides were the reverse complement of the six nucleotides at the 3′ end of the miRNA. The reverse-transcribed product was amplified using the miRNA-specific forward primers and the universal reverse primers. The three RT-qPCR methods to detect miRNAs have their own advantages and disadvantages respectively. Poly(A)-tailing and stem-loop methods are mainly used to detect mature miRNAs. They cannot distinguish between different miRNAs in a family if they produce the same mature miRNA. Therefore, to detect different types of miRNAs, the specific precursors must be analyzed[8]. Mou et al.[15] reported that stem-loop RT-qPCR and poly(A)-tailing RT-qPCR can detect highly abundant miRNAs at similar levels of accuracy and specificity. However, stem-loop RT-qPCR cannot detect low abundant miRNAs, suggesting poly(A)-tailing RT-qPCR may be a better option for miRNA analysis. In this study, we selected poly(A)-tailing RT-qPCR to detect mature miRNAs because the polyadenylated miRNA reverse transcription template enabled universal miRNA detection. This allowed the detection of numerous miRNAs using one template set. Another reason we used poly(A)-tailing RT-qPCR was that stem-loop RT-qPCR was more expensive (e.g., higher costs associated with specific primer synthesis). Moreover, miRNA precursor RT-qPCR provides complementary data for a more comprehensive characterization of miRNA expression.

There is a lack of consensus regarding how best to perform and interpret RT-qPCR experiments. Many technical defects can affect the accuracy of RT-qPCR analysis. Therefore, the minimum information required to publish RT-qPCR experiments (MIQE) [16] and the Real-Time PCR Data Markup Language (RDML) were published[17]. These standards for RT-qPCR experiments focus on the reliability and consistency of results. According to the MIQE, the selection of suitable reference genes is one of the essential components to ensure the accuracy of a RT-qPCR assay. This is because RT-qPCR results are influenced by factors such as variations in RNA extraction, reverse transcription, and efficiency of amplification. Thus, data normalization of RT-qPCR using reference genes enables comparisons of different mRNA concentrations from various samples [16]. Ideal reference genes should be stably expressed between different plant varieties, tissues, stages of development, and biotic or abiotic stress conditions[18, 19]. However, numerous reports have determined that candidate reference genes may be stably expressed only under certain conditions [20–24]. It is necessary to identify and select suitable reference genes through specific experimental procedures [16]. Unfortunately, there is no universally accepted method for selecting reference genes and assessing stability. GeNorm[25], NormFinder[26], and BestKeeper[27] are three algorithms widely used to analyze the stability of candidate reference genes. Using different algorithms to evaluate the stability of reference gene expression may facilitate the selection of reliable reference genes for precise data normalization of RT-qPCR.

Soybean (*Glycine max*) is an important crop for seed protein and edible oil production. In our earlier study, the miRNAs and transcriptional profiles of genes associated with several stress responses were sequenced using deep sequencing technology [28, 29]. The objective of the previous study was to confirm the accuracy of deep sequencing technology and determine the variations in expression of mature miRNAs, miRNA precursors, and their targeted mRNAs in soybean under various abiotic stress conditions. In the current study, we aimed to identify suitable reference mRNA/miRNAs from the eight traditional candidate reference mRNA genes and eight candidate reference miRNAs. Soybean roots and leaves exposed to various abiotic stress conditions (PEG-simulated drought, salinity, alkalinity, salinity+alkalinity, and abscisic acid) that were used for relative quantification analysis. We used soybean cultivar ‘*Williams 82*’ of its known genome sequence [30]. Statistical analysis was completed using the geNorm, NormFinder, and BestKeeper algorithms. To the best of our knowledge, this study is the first to use the entire set of reference mRNA/miRNAs for accurate RT-qPCR data normalization for mature miRNAs, miRNA precursors, and their targeted mRNAs in soybean leaf or root tissues exposed to various abiotic stresses.

## Materials and Methods

### Plant materials and abiotic stress treatments

Soybean seeds (‘*Williams 82*’) were treated with ethanol for 10 min and then rinsed several times with sterile distilled water. The seeds were cultured in Hoagland nutrient solution and grown at 30°C with a 16 h light/8 h dark photoperiod (80 μmol m^−2^ s^-1^ photon flux density) and 50% relative humidity. When the first pair of unifoliate leaves fully opened, we initiated stress treatments as follows: PEG-simulated drought (8% PEG 8000), salinity (120 mM NaCl), alkalinity (100 mM NaHCO_3_), salinity+alkalinity (70 mM NaCl+50 mM NaHCO_3_), and ABA (200 μM ABA). Untreated plants were used as controls. The seedlings were incubated for 0, 1, 3, 6, 9, and 12 h, with leaf and root samples collected at each time point. Three biological replicates were performed for each stress treatment. All samples were immediately frozen in liquid nitrogen and stored at −80°C until required. All samples information above can be found in S1 Table.

### RNA extraction and quality controls

Total RNA was extracted using RNAiso Plus (Takara, Japan) according to the manufacturer’s instructions. The purified RNA was analyzed using a Thermo Scientific NanoDrop2000 spectrophotometer. A 260 nm/280 nm optical density ratio of 1.8–2.0 indicated high quality RNA. The RNA integrity was checked by 1% agarose gel electrophoresis. Clearly visible RNA bands and a 25S/18S ratio close to 2:1 indicated intact RNA.

### Primer designing and their validation

We selected eight candidate reference mRNA genes for data normalization of mRNAs and miRNA precursors during RT-qPCR analysis. We also selected eight candidate reference miRNAs for data normalization of mature miRNAs. The selection of the candidate reference mRNA/miRNAs was based on previous reports [20, 21, 31–39]. The mRNA sequences were obtained from the Phytozome 10.3 website [40] (http://phytozome.jgi.doe.gov/pz/portal.html#!info?alias=Org_Gmax) and the miRNA sequences were obtained from the miRBase(Release 21) website [41] (http://www.mirbase.org/). Primers were designed using DNAstar software[42]. The mRNA primers were designed possibly across two exons while the miRNA precursor primers were designed at stem-loop regions [8, 43]. Primers were designed to amplify mRNA and miRNA precursor products of 80–200 bases. The possible secondary structures were assessed using Mfold[44] (http://unafold.rna.albany.edu/?q=mfold) and the amplicon specificity of the primers was analyzed using BLAST tools (http://blast.ncbi.nlm.nih.gov/Blast.cgi)[45]. Additionally, the miRNA-specific forward primers were designed using the NCode VILO miRNA cDNA Synthesis Kit (Invitrogen) following the manufacturer’s protocol. The miRNA universal reverse primers were provided in the kit. The designed primer sequences were listed in Table 1. All primers were synthesized at the Suzhou GENEWIZ Biological Technology Services Company, China.

**Table 1:** Descriptions of mRNAs and miRNAs and RT-qPCR amplification characteristics.

Symbol	Name	Annotation	Accession No	Primer design	Amplicon length(bp)	Amplicon Tm(°C)	Regression coefficient (R^2^)	PCR efficiency (%)
**Candidate reference mRNA genes for microRNA and miRNA precursor normalization**								
***Act***	*Actin*	Cytoskeletal structural protein	(phytozome)Glyma.02G091900.1	F: GACCTTCAACACCCCTGCT	143bp	85.0°C	1.000	101.8%
				R: GTGGGAGTGCATAACCCTC				
***Cyp***	*Cyclophilin*	Cyclophilin type peptidyl- prolyl cis-trans isomerase activity	(phytozome)Glyma.12G024700.1	F: TCCGAGCACCGCCGAGAACT	135bp	91.0°C	0.999	99.8%
				R: AAGTCGCCGCCCTGGCACAT				
***EF1a***	*Elongation Factor 1-alpha*	translation elongation factor	(phytozome)Glyma.19G052400.1	F: GCTCTTCTTGCTTTCACCCTT	111bp	82.5°C	0.999	106.8%
				R: TTCCTTCACAATTTCATCATACC				
***EF1b***	*Elongation Factor1- beta*	translation elongation factor	(phytozome)Glyma.14G039100.1	F: TGGTGATGAGACAGAGGAGGA	106bp	83.0°C	1.000	98.1%
				R: AACATCGAGAAGGACAGAAGA				
***Fbox***	*F-box*	F-box protein	(phytozome)Glyma.12G051100.1	F: CATCCGTGCTAATGATATTGT	127bp	83.5°C	0.996	107.8%
				R: TGATGGTGTTGGTAGAGGC				
***TuB***	*Beta Tubulin*	Structural constituent of cytoskeleton	(phytozome)Glyma.19G127700.1	F: CCAGTTGGTGGAGAATGCT	129bp	86.0°C	0.996	92.9%
				R: ATGGTTGCGGAGATCAAGTGA				
***TuA***	*Alpha Tubulin*	Structural constituent of cytoskeleton	(phytozome)Glyma.05G157300.1	F: TTGCCACCATCAAGACTAAGA	104bp	86.0°C	0.998	95.9%
				R: ACCACCAGGAACAACAGAAG				
***60s***	*60s ribosomal*	60s ribosomal protein	(phytozome)Glyma.13G318800.1	F: GTCCTGAAGGCCATTCCTAAG	132bp	82.5°C	0.998	100%
				R: ACTGATGCCCTGCTCCAACT				
**Candidate reference miRNAs for mature microRNA normalization**								
**156a**	Mature gma-miR156a-5p	Mature miRNA	(miRBase) MIMAT0001686	F: TGACAGAAGAGAGTGAGCACA	- ^b^	78.5°C	0.999	108.8%
				R: Universal RT Primer^a^				
**166a**	Mature gma-miR166a-3p	Mature miRNA	(miRBase)MIMAT0001677	F: GGACCAGGCTTCATTCCCCA	-	80.5°C	0.999	97.1%
				R: Universal RT Primer				
**167a**	Mature gma-miR167a-5p	Mature miRNA	(miRBase) MIMAT0001679	F: TGAAGCTGCCAGCATGATCTA	-	79.0°C	0.999	94.5%
				R: Universal RT Primer				
**171a**	Mature gma-miR171a-3p	Mature miRNA	(miRBase)MIMAT0007358	F: TGAGCCGTGCCAATATCACGA	-	79.5°C	0.998	107.5%
				R: Universal RT Primer				
**172a**	Mature gma-miR172a-3p	Mature miRNA	(miRBase) MIMAT0001682	F: GAGAATCTTGATGATGCTGCAT	-	78.0°C	0.999	100.3%
				R: Universal RT Primer				
**393a**	Mature gma-miR393a-5p	Mature miRNA	(miRBase)MIMAT0007362	F: TCCAAAGGGATCGCATTGATCA	-	78.5°C	0.993	100.1%
				R: Universal RT Primer				
**397a**	Mature gma-miR397a-5p	Mature miRNA	(miRBase) MIMAT0021627	F: TCATTGAGTGCAGCGTTGATGA	-	79.0°C	0.998	104.7%
				R: Universal RT Primer				
**1520d**	Mature gma-miR1520d-3p	Mature miRNA	(miRBase) MIMAT0007379	F: ATCAGAACATGACACGTGACAA	-	78.5°C	0.991	108.7%
				R: Universal RT Primer				
**Reference mRNA and miRNA validation**								
**396a**	Mature gma-miR396a-5p	Mature miRNA	(miRBase)MIMAT0001687	F: TTCCACAGCTTTCTTGAACTGA	-	78.5°C	0.998	98%
				R: Universal RT Primer				
**Pre-396a**	Precursors gma-miR396a	Mature precursors	(miRBase)MI0001785	F: GCTTTCTTGAACTGCATCCAA	94bp	81.0°C	0.999	101.6%
				R: TCCCACAGCTTTATTGAACCG				
***GRF9***	*Growth Regulating Factor 9*	Transcription factors	(phytozome)Glyma.01G144900.1	F: GATGGAAAGAAATGGAGGTG	137bp	79.0°C	0.999	107.3%
				R: GGCTTTGTTGCCAGATGAG				

^a^miRNA universal reverse primers were provided in the NCode VILO miRNA cDNA Synthesis Kit (Invitrogen).

^b^Amplicon length of the miRNA precursors cannot be determined.

Robust and precise RT-qPCR assays are usually correlated with high PCR efficiency[16]. The PCR amplification efficiency should be calculated using the formula E = 10^(−1/Slope)^-1 and the slope of the calibration curve. Calibration curves were produced using mixed cDNA samples as a starting template and five or six replicates of 10-, 4-, and 2-fold serial dilutions to create a gradient of concentrations. The Mx3000P RT-qPCR system automatically generated calibration curves. Only primers with PCR amplification efficiencies close to 100% were used. Furthermore, the amplicon specificity for all candidate reference mRNA/miRNAs was confirmed by the presence of a single peak.

### cDNA synthesis and qPCR

The mRNA and miRNA precursor expression levels were determined using standard RT-qPCR [7]. Following treatment with gDNA Eraser, 1 μg total RNA was used as the template for first-strand cDNA synthesis with the PrimeScript RT Reagent Kit with gDNA Eraser (Takara, Japan). Reverse transcription was completed with oligo(dT) and random hexamer primers. The cDNA was diluted 10-fold for mRNA and miRNA precursor analysis. The expression of mature miRNA was measured using Poly(A)-RT-qPCR[10]. Total RNA including miRNA was reverse transcribed using the NCode VILO miRNA cDNA Synthesis Kit according to the manufacturer’s protocol. The miRNA was polyadenylated using ATP and poly-A polymerase, and cDNA was synthesized by reverse transcribing the tailed RNA using the universal RT Primer in a single reaction volume of 20 μl. We diluted the cDNA 10-fold for miRNA analysis stored at −20°C until required.

The RT-qPCR was completed in 96-well blocks using an Applied Mx3000P Real-Time Thermocycler (Stratagene). The reaction mixtures were prepared using the SYBR Premix Ex Taq II kit (Takara, Japan). The primer sequences were listed in Table 1. The RT-qPCR program consisted of an initial step at 95°C for 2 min to activate the Taq DNA polymerase, followed by 40 cycles of 94°C for 5 s and 62°C for 20s. We verified the amplicons specificity by melting curve analysis from 62°C to 95°C. Each reaction was completed with three technical replicates. However, three biological replicates have been pooled together after cDNA synthesis for expression stability assessment of candidate reference mRNA/miRNAs. All candidate reference mRNA genes or candidate reference miRNAs were quantified using the same batch of cDNA to minimize experimental variation. The no-template controls were included in each sample batch.

### Analysis of gene expression stability

We initially examined the expression stability of eight candidate reference mRNA genes and eight candidate reference miRNAs using boxplot analysis[46]. Boxplots of the quantification cycle (Cq) values for each candidate reference mRNA/miRNA were assessed using MATLAB software. The expression variation range of Cq values (ΔCq) was calculated using the formula ΔCq = Cq_max_ − Cq_min_. Then we used geNorm[25] analysis (https://genorm.cmgg.be/). The sample with the highest expression level (i.e., with the lowest Cq value) for each gene was used as a control with an expression level of 1. The relative expression levels for the other samples were calculated using the formula 2^−(average Cq samples − average Cqmin)^. The obtained data were analyzed to determine the gene stability value (M) for each candidate reference mRNA/miRNA. Additionally, the average pairwise variation (V_n/n+1_) was calculated to determine the optimal number of reference mRNA/miRNAs. Next we analyzed the samples using NormFinder [26] (http://moma.dk/normfinder-software). The raw average Cq values were converted to linear scale expression quantities using a standard curve. The expression stability (S) values and best gene combination between inter-group were assessed by NormFinder. We also completed BestKeeper[27] analysis (http://gene-quantification.com/bestkeeper.html), which was based on average raw Cq values of the candidate reference mRNA/miRNAs. The pair-wise correlation assessments of all candidate mRNA/miRNAs pairs were conducted and geometric expression means were calculated. BestKeeper algorithm identified the most stably expressed gene based on the highest correlation coefficient. Finally, the consensus rankings of the candidate reference mRNA/miRNAs were determined according to the geometric rank means from three analyses (i.e., geNorm, NormFinder, and BestKeeper).

### Validation of gene expression stability

To confirm the identities of the most stable candidate reference mRNA/miRNAs and the best reference combinations, the relative expression levels of *Growth Regulating Factor 9* (*GRF9*), precursor gma-miR396a (Pre-396a), and mature gma-miR396a-5p (396a) in leaf and root samples treated for 3 h under various abiotic stresses(PEG-simulated drought, salinity, alkalinity, salinity+alkalinity, and abscisic acid)were determined using RT-qPCR. Each reaction was completed with three technical and three biological replicates. The primer details are listed in Table 1. The relative expression levels were calculated using the 2^−ΔΔCt^ formula[5]. The significance of the expression level differences was determined using one-way analysis of variance in SPSS 13.0.

## Results

### RNA quality and characteristics of RT-qPCR amplified products

RNA quality, primer specificity, and amplification efficiency are key issues related to RT-qPCR according to the MIQE[16] guidelines. The ribosomal RNA bands observed during 1% agarose gel electrophoresis were clearly visible and the 25S:18S ratio was approximately 2:1 (S1A Fig). The 260 nm/280 nm optical density ratio ranged from 1.8 to 2.0 (S1B Fig), indicating the RNA quality was appropriate for RT-qPCR assays.

The candidate reference mRNA/miRNAs were selected based on their amplification efficiency and specificity. The gene-related information and RT-qPCR amplified product characteristics were listed in Table 1. The primer pair locations on transcript sequences are indicated in S2 File. Primer specificity was confirmed based on the dissociation curves of the amplicons consisted of a single peak (S1 File). The amplification efficiency for the tested gene primers varied from 94.5 to 108.8% (Table 1), and the correlation coefficients of standard curves (S1 File) were between 0.991 and 1.000 (Table 1).

### Candidate reference mRNA/miRNA expression profiles

**Fig 1:**
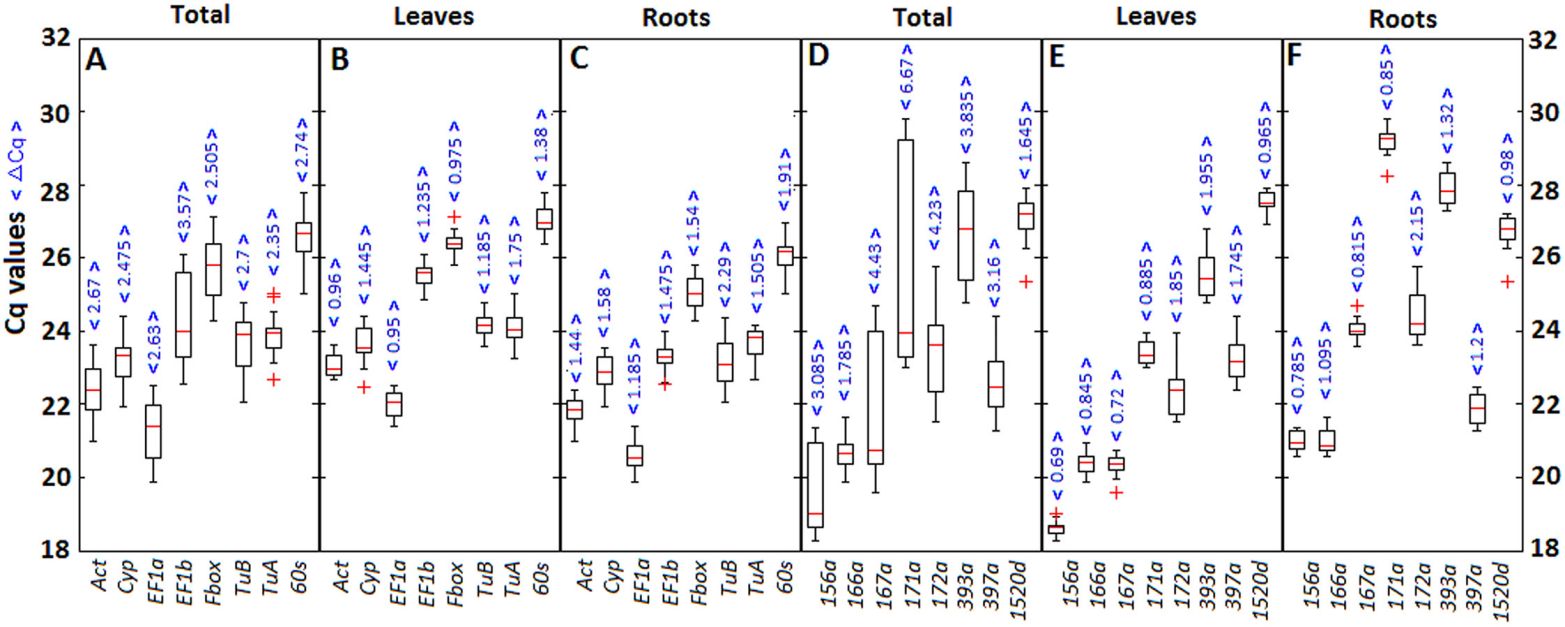
Boxplots of the quantification cycle (Cq) values of candidate reference mRNAs and candidate reference miRNAs. Boxplots of Cq values for each candidate reference mRNA/miRNAs were assessed in sets of samples, respectively(S1 Table). (A) Candidate reference mRNA genes were analyzed in leaf and root combined samples (n = 52). (B) Candidate reference mRNA genes were analyzed in leaf samples (n = 26). (C) Candidate reference mRNA genes were analyzed in root samples (n = 26). (D) Candidate reference miRNAs were analyzed in leaf and root combined samples (n = 52). (E) Candidate reference miRNAs were analyzed in leaf samples (n = 26). (F) Candidate reference miRNAs were analyzed in root samples (n = 26). The box indicates the 25th and 75th percentiles. The line across the box corresponds to the median, and whiskers represent the maximum and minimum values. The plus sign indicates the maximum or minimum outlier.

The boxplots of Cq values for each candidate reference mRNA/miRNA were assessed in sets of samples respectively (Fig 1). The sets of samples were listed in S1 Table. The Cq values for each candidate reference mRNA/miRNA were ranged in 18–30 cycles, which corresponded to a wide dynamic normalization range (Fig 1).

Because the cDNA templates for the different samples were reverse transcribed from the same amount of total RNA, we assumed that ΔCq was related to the expression stability of candidate reference mRNA/miRNAs. A narrow range of Cq values indicated more stable expression. The ΔCq for each candidate reference mRNA gene in leaf and root combined samples ranged from 2.35 to 3.57 cycles (Fig 1A), while the ΔCq for each candidate reference miRNA in leaf and root combined samples ranged from 1.785 to 6.67 cycles (Fig 1D). The ΔCq values indicated a wider inter-tissue range. The ΔCq for each candidate reference mRNA gene in leaf samples ranged from 0.95 (*EF1a*) to 1.75 (*TuA*) cycles (Fig 1B), while in root samples it ranged from 1.185 (*EF1a*) to 2.29 (*TuB*) cycles (Fig 1C). The ΔCq for each candidate reference miRNA in leaf samples ranged from 0.69 (156a) to 1.955 (393a) cycles (Fig 1E), while in root samples it ranged from 0.785 (156a) to 2.15 (172a) cycles (Fig 1F). The ΔCq values exhibited a narrow intra-tissue range. All results indicated that the intra-tissue candidate reference mRNA/miRNAs expression was more stable than the inter-tissue expression. However, comparisons of ΔCq were insufficient, which required further assessments using three statistical algorithms to validate the expression stability of the candidate reference mRNA/miRNAs.

### GeNorm assessment of expression stability

**Fig 2:**
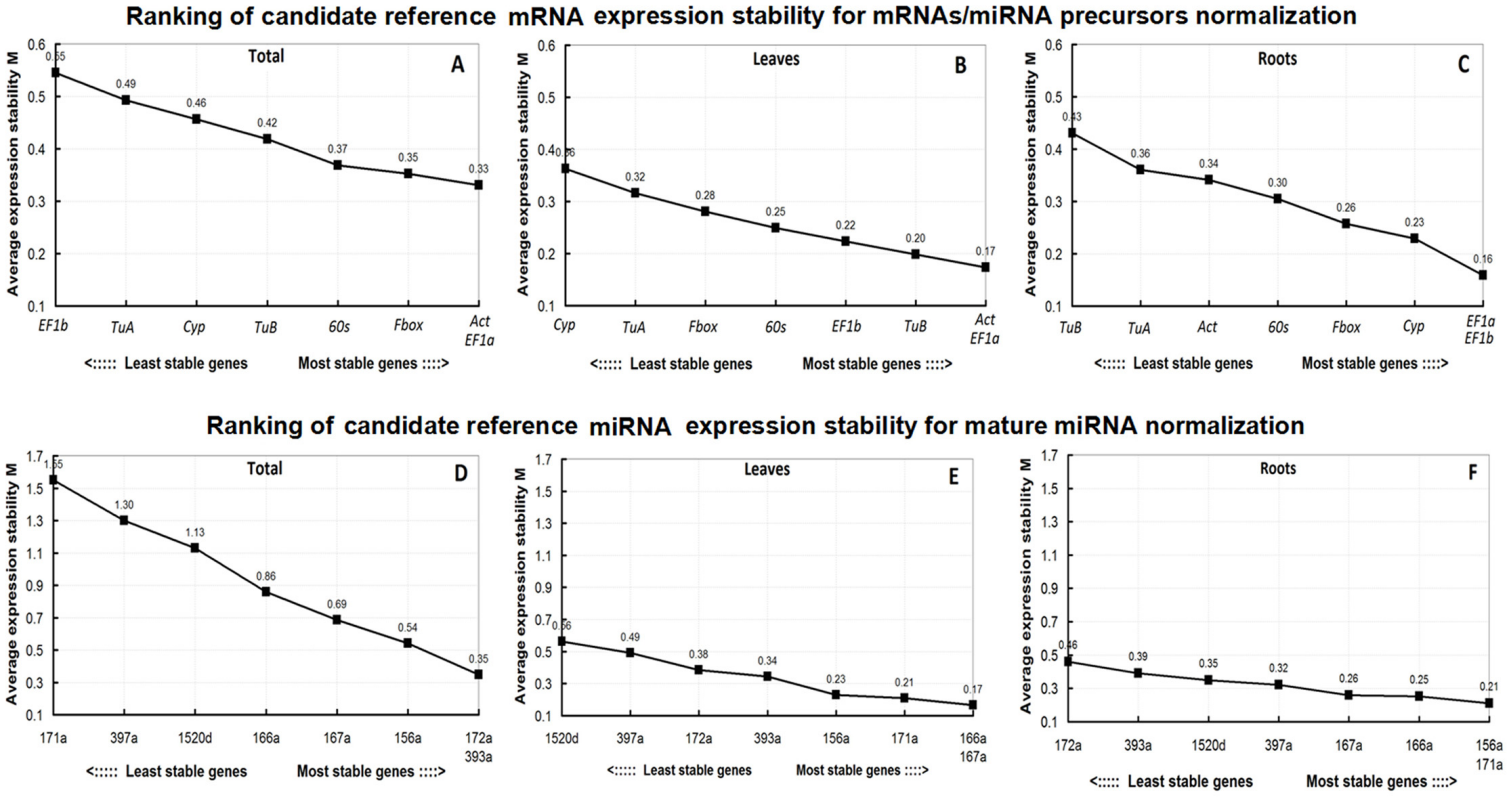
Ranking of the candidate reference mRNAs and candidate reference miRNAs by geNorm. The ranking of candidate reference mRNA/ miRNAs was based on average expression stability values (M). A lower M value indicates a more stable expression level. The six tested groups are the same as those indicated in Fig 1 (Group A to F).

**Fig 3:**
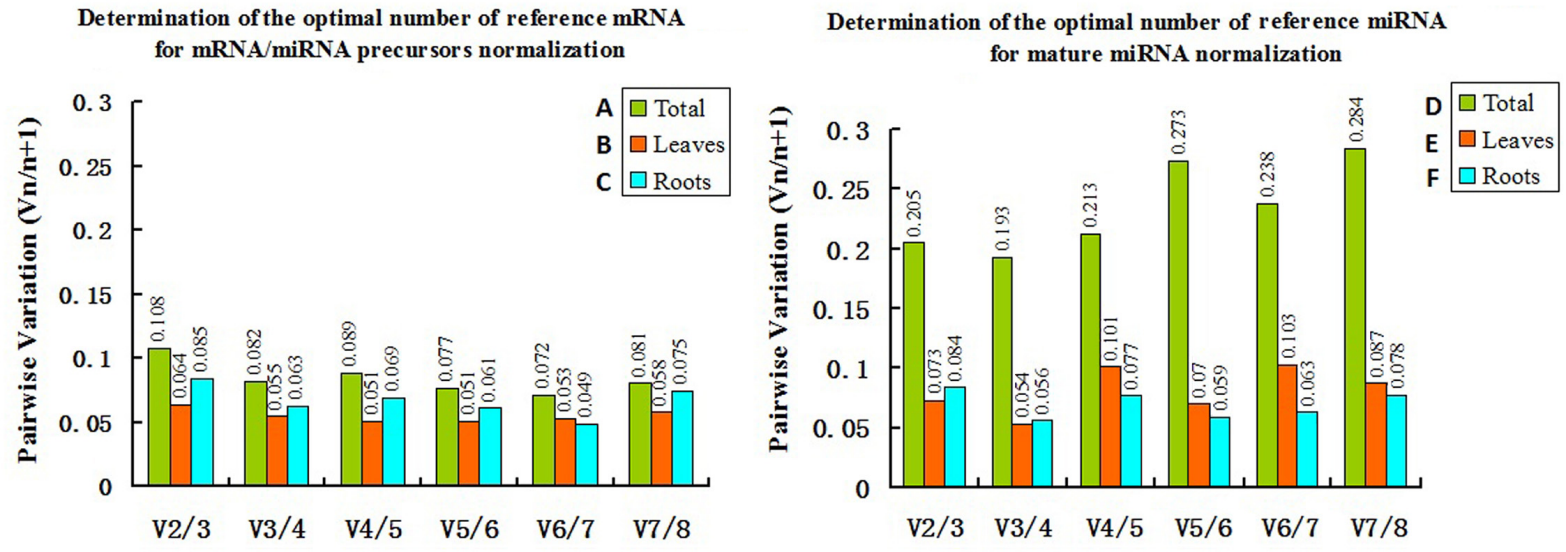
Determination of the optimal number of reference mRNAs and candidate reference miRNAs for normalization according to pairwise variation (V_n/n+1_) using geNorm. V_n/n+1_ > 0.15 indicated an additional (n + 1) reference was required while V_n/n+1_ ≤ 0.15 indicated an additional reference was not required. The six tested groups are the same as those indicated in Fig 1 (Group A to F).

The geNorm algorithm eliminates the least stable reference gene and then recalculates the average M values for the remaining candidate reference genes. The genes with smaller M values were the more stably expressed genes [25]. Additionally, the optimal number of reference genes was determined based on pairwise variation (V_n/n+1_). If V_n/n+1_ > 0.15_,_ an additional (n + 1) reference gene was required, but if V_n/n+1_ ≤ 0.15, an additional (n + 1) reference gene was not required[25]. The assessment of candidate reference mRNA/miRNAs expression stability was completed in sets of samples, respectively (same as in Fig 1A to 1F) using geNorm. In stepwise analysis, we observed that the M values of the most stable candidate reference mRNAs and candidate reference miRNAs in leaf and root combined samples were bigger than those of leaf or root samples with M values of *EF1a*/*Act* (0.33) > *EF1a*/*Act* (0.17) or *EF1a/EF1b* (0.16) (Fig 2A, 2B and 2C) and 172a/393a (0.35) > 166a/167a (0.17) or 156a*/*171a (0.21) (Fig 2D, 2E and 2F). Meanwhile, the V_n/n+1_ values of the candidate reference mRNAs and candidate reference miRNAs for leaf and root combined samples all were greater than those of leaf or root samples (Fig 3), the data demonstrated that the best combinations of reference mRNA/miRNAs was better in leaf or root tissues than in leaf and root combined samples. More specifically, the V_n/n+1_ values of the candidate reference miRNAs for leaf and root combined samples all were higher than the threshold value of 0.15 (Fig 3), it indicated we could not get suitable combination of reference miRNAs for leaf and root combined samples. This results was consistent with the results of the ΔCq analysis. The intra-tissue candidate reference mRNA/miRNAs expression was more stable than the inter-tissue expression. Therefore, we subsequently analyzed the gene expression stability of candidate reference mRNA/miRNAs in leaf and root samples.

The geNorm gene expression stability results for candidate reference mRNA/miRNAs in leaf and root samples are provided in Table 2 and Fig 2. Regarding candidate reference mRNA genes for mRNA and miRNA precursor data normalization, the most stable reference mRNA (i.e., with the lowest M values) in leaf and root samples were *EF1a*/*Act* (M value: 0.17) and *EF1a/EF1b* (M value: 0.16), respectively. The most unstable reference mRNA (i.e., with the highest M values) were *Cyp* (M value: 0.36) and *TuB* (M value: 0.43), respectively. In terms of candidate reference miRNAs for mature miRNA data normalization, the most stable reference miRNA in leaf and root samples were 166a/167a (M value: 0.17) and 156a*/*171a (M value: 0.21), respectively. The most unstable reference miRNA were 1520d (M value: 0.56) and 172a (M value: 0.46), respectively. Additionally, to determine the optimal number of candidate reference mRNA genes for data normalization in leaf and root samples, the V_2/3_ values were 0.064 and 0.085, respectively (Fig 3B and 3C); for candidate reference miRNAs in leaf and root samples, the V_2/3_ values were 0.073 and 0.084, respectively (Fig 3E and 3F). To determine the optimal number of candidate reference mRNA/miRNAs for data normalization in leaf and root samples all V_2/3_ values were lower than the cut-off value of 0.15 (Fig 3B, 3C, 3E and 3F), which meant a third reference mRNA/miRNA was unnecessary. Therefore, the optimal number for both the reference mRNA/miRNAs was 2. The best reference mRNA combinations for mRNA and miRNA precursor data normalization in leaf and root samples were *EF1a* + *Act* and *EF1a + EF1b*, respectively. The best reference miRNA combinations for mature miRNA data normalization in leaf and root samples were 166a *+* 167a and 171a *+* 156a, respectively.

**Table 2:** Ranking of candidate reference mRNAs and candidate reference miRNAs using the geNorm, NormFinder, and BestKeeper algorithms.

Symbol	Leaf tissue samples in soybean(n = 26, S1 Table)							Root tissue samples in soybean(n = 26, S1 Table)						
	GeNorm		NormFinder		BestKeeper			GeNorm		NormFinder		BestKeeper		
	M	Rank	S	Rank	r	Rank	Con	M	Rank	S	Rank	r	Rank	Con
**Ranking of candidate reference mRNA genes for microRNA and miRNA precursor normalization**														
***Act***	0.17	1	0.099	2	0.95	1	**1**	0.34	6	0.146	4	0.81	6	**6**
***Cyp***	0.36	8	0.198	8	0.65	8	**8**	0.23	3	0.191	7	0.81	5	**5**
***EF1a***	0.17	1	0.114	4	0.93	2	**2**	0.16	1	0.145	3	0.82	4	**2**
***EF1b***	0.22	4	0.109	3	0.90	4	**4**	0.16	1	0.106	1	0.87	1	**1**
***Fbox***	0.26	6	0.160	6	0.67	7	**6**	0.26	4	0.149	5	0.85	2	**4**
***TuB***	0.20	3	0.093	1	0.91	3	**2**	0.43	8	0.240	8	0.74	8	**8**
***TuA***	0.32	7	0.164	7	0.81	6	**7**	0.36	7	0.157	6	0.76	7	**7**
***60s***	0.25	5	0.117	5	0.87	5	**5**	0.30	5	0.141	2	0.85	3	**3**
**Best gene**	*EF1a/Act*		*TuB*		*Act*		***Act***	*EF1a/ EF1b*		*EF1b*		*EF1b*		*EF1b*
**Worst gene**	*Cyp*		*Cyp*		*Cyp*		*Cyp*	*TuB*		*TuB*		*TuB*		*TuB*
**Best combination**	*EF1a & Act* (0.064)		*EF1b & Act* (0.063)					*EF1a & EF1b* (0.085)		*60s & EF1B* (0.081)				
**Ranking of candidate reference miRNAs for mature miRNA normalization**														
**156a**	0.23	4	0.080	5	0.63	6	**4**	0.21	1	0.109	4	0.89	2	**2**
**166a**	0.17	1	0.021	1	0.95	1	**1**	0.25	3	0.102	3	0.88	3	**4**
**167a**	0.17	1	0.032	2	0.88	3	**2**	0.26	4	0.067	1	0.95	1	**1**
**171a**	0.21	3	0.034	3	0.91	2	**3**	0.21	1	0.095	2	0.87	4	**2**
**172a**	0.38	6	0.180	6	0.80	5	**6**	0.46	8	0.225	8	0.82	6	**8**
**393a**	0.34	5	0.209	7	0.87	4	**5**	0.39	7	0.143	7	0.86	5	**5**
**397a**	0.49	7	0.218	8	0.31	8	**8**	0.32	5	0.142	6	0.68	8	**7**
**1520d**	0.56	8	0.061	4	0.56	7	**7**	0.35	6	0.129	5	0.72	7	**6**
**Best miRNA**	166a /167d		166a		166a		**166a**	171a/156a		167a		167a		**167a**
**Worst miRNA**	1520d		397a		397a		**397a**	172a		172a		397a		**172a**
**Best combination**	166a & 167d (0.073)		166a & 167a (0.020)					171a & 156a (0.084)		167a & 171a (0.067)				

M: Stability value determined by geNorm analysis. A lower M value indicates higher expression stability.

S: Stability value determined by NormFinder analysis. A lower S value indicates higher expression stability.

r: Pearson correlation coefficient determined by BestKeeper analysis. The most stably expression has the highest correlation.

Con: Consensus ranking, which corresponds to the geometric mean of ranks determined by the geNorm, NormFinder, and BestKeeper algorithms.

### NormFinder assessment of expression stability

NormFinder was an algorithm to identify the optimal reference gene for data normalization among a set of candidate reference genes [26]. It ranked the expression stability of the candidate reference genes according to their S values. The lowest S values represented the most stable expression levels. The S values and rank order of the candidate reference mRNA/miRNAs are provided in Table 2 and S3 File. Regarding candidate reference mRNA genes for mRNA and miRNA precursor data normalization, the most stably expressed reference mRNA were *TuB* (S value: 0.093) in leaf samples and *EF1b* (S value: 0.106) in root samples. The most unstable reference mRNA were *Cyp* (S value: 0.198) in leaf samples and *TuB* (S value: 0.240) in root samples. *TuB* was the most stable gene in leaf samples, but the least stable gene in root samples. In terms of candidate reference miRNAs for mature miRNA data normalization, the most stable reference miRNA were 166a (S value: 0.021) in leaf samples and 167a (S value: 0.067) in root samples. The most unstable reference miRNA were 397a (S value: 0.218) in leaf samples and 172a (S value: 0.225) in root samples.

NormFinder uses a solid statistical framework to estimate the overall variability in expression of the candidate genes and the variations among tested sample subgroups [26]. Using NormFinder, we calculated intra-group and inter-group variations among the five groups exposed to different abiotic stresses (Table 2 and S3 File). We determined that *EF1b* + *Act* (S value: 0.063) was the best mRNA combination in leaf samples and *60s* + *EF1B* (S value: 0.081) in root samples for mRNA and miRNA precursor data normalizations. In leaf and root samples, the best miRNA combinations for mature miRNA data normalization were 166a + 167a (S value: 0.020) and 167a + 171a (S value: 0.067), respectively.

### BestKeeper assessment of expression stability

The BestKeeper algorithm evaluates gene expression stability based on the standard deviation (SD), coefficient of variation (CV), and Pearson correlation coefficient (r). Estimating the reference gene expression stability based on the SD and CV values, reference genes with SD values higher than 1 should be excluded. The BestKeeper Index is calculated as the geometric mean of each candidate gene. This index is used along with a pair-wise correlation analysis of all pairs of candidate genes to identify the optimal reference genes[27]. A high r value indicates the reference gene pairs have very similar expression patterns, which makes them stable reference genes. The BestKeeper analysis results are provided in Table 2 and S4 File. Each candidate reference mRNA/miRNA had low variation values with SD values below 1. The r values suggested that the most suitable reference mRNAs for mRNA and miRNA precursor data normalization were *Act* (r value: 0.95) in leaf samples and *EF1b* (r value: 0.87) in root samples. We determined that the most suitable reference miRNAs for mature miRNA normalization was 166a (r value: 0.95) in leaf samples and 167a (r value: 0.95) in root samples. *Cyp* (r value: 0.65) and *TuB* (r value: 0.74) were the most unsuitable reference mRNAs for mRNA and miRNA precursor data normalization in leaf and root samples, respectively. 397a was the most unsuitable reference miRNA for mature miRNA normalization in leaf (r value: 0.31) and root (r value: 0.68) samples.

### Comprehensive assessment of expression stability

The summarized data in Table 2 indicated that the geNorm, NormFinder, and BestKeeper algorithms produced similar rank orders for the candidate reference mRNA/miRNAs expression stability. However, the results lacked consistency, which may be due to differences in calculation methods among the three algorithms.

We also determined the consensus rank of each candidate reference mRNA/miRNAs using geometric means of the rankings determined by the geNorm, NormFinder, and BestKeeper algorithms (Table 2). Based on a comprehensive assessment, we identified *Act* and *EF1b* as the most suitable reference mRNA for mRNA and miRNA precursor data normalization in leaf and root samples, respectively, while the most unsuitable candidate reference mRNA were *Cyp* and *TuB* in leaf and root samples, respectively. We determined that 166a and 167a were the most suitable reference miRNA for mature miRNA data normalization in leaf and root samples, respectively, while the most unsuitable candidate reference miRNA were 397a and 172a in leaf and root samples, respectively.

### Validation of reference mRNA/miRNA for relative quantification

**Fig 4:**
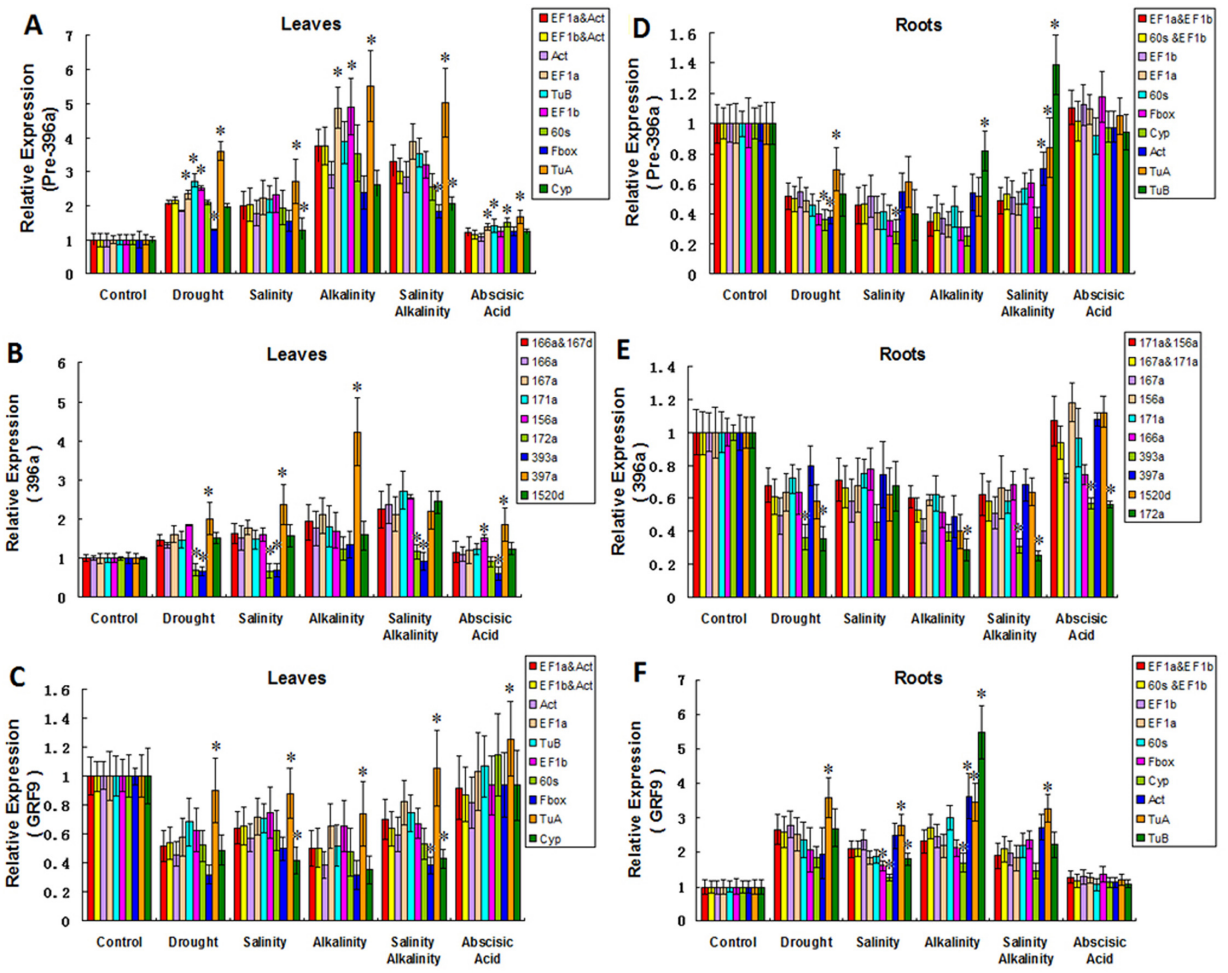
Validation of reference mRNAs and reference miRNAs for relative quantification under various stress conditions. Comparison of Pre-396a, 396a, and *GRF9* relative expression levels determined using data normalized with the best reference mRNA/miRNA combination (according to geNorm and NormFinder) and each candidate reference mRNA/miRNA (consensus ranking from best to worst). (A) Pre-396a normalized by the best reference mRNA combination and each candidate reference mRNA in leaf samples; (B) Pre-396a normalized by the best reference mRNA combination and each candidate reference mRNA in root samples; (C) Mature miR396 normalized by the best reference miRNA combination and each candidate reference miRNA in leaf samples; (D) Mature miR396 normalized by the best reference miRNA combination and each candidate reference miRNA in root samples; (E) *GRF9* normalized by the best reference mRNA combination and each candidate reference mRNA in leaf samples; (F) *GRF9* normalized by the best reference mRNA combination and each candidate reference mRNA in root samples. Samples were treated for 3 h with various stresses (PEG-simulated drought, salinity, alkalinity, salinity+alkalinity, and ABA). The error bars represent the standard deviations (SD) of three biological replicates. *P* < 0.05 (instead of *) indicated a significant difference according to one way analysis of variance between the data normalized using the recommend combinations or the best reference mRNA/miRNA and the data normalized using the other candidate ones.

To validate the accuracy of the selected reference mRNA/miRMA in analyses of relative quantification, we compared the relative expression levels of Pre-396a, 396a and *GRF9*, calculated using the best combination indicated by geNorm and NormFinder and each candidate reference mRNA/miRMA. As indicated in Fig 4, the relative quantities of Pre-396a, 396a, and *GRF9* calculated using data normalized by the best combination and the most stable reference mRNA/miRNA were similar. Except for ABA treatment, all abiotic stresses produced the expected expression tendencies for each gene in leaf and root samples. For example, we observed up-regulated Pre-396a and 396a expression (Fig 4A and 4B) and down-regulated *GRF9* expression (Fig 4C) in leaf samples. In contrast, the expression of Pre-396a and 396a were down-regulated (Fig 4D and 4E) while the expression of *GRF9* was up-regulated in leaf samples (Fig 4F). As reported previously, miR396 expression is affected by various environmental stresses [28,47–51]. Our results indicated that there was a positive correlation between Pre-396a and 396a expression levels, which is likely because 396a is derived from Pre-396a. The gene expression levels of 396a and *GRF9* were inversely correlated, probably because *GRF* genes are miR396 targets in plants [51–53]. However, following ABA-treatment, there were no obvious changes to *GRF9*, Pre-396a, and 396a expression levels. This was consistent with the results of previous studies that concluded miR396 gene expression was insensitive to ABA[54]. All of our results were in agreement with those of other reports, which increased our confidence in the reliability and viability of the selected reference mRNA/miRNAs. However, one way analysis of variance detected significant differences between the results generated using recommended mRNA/miRNA combinations or the most stable reference mRNA/miRNA and the results produced using the other candidate reference mRNA/miRNAs. Significant differences were observed for the results from the same experimental conditions. For example, in some instances, *TuA* as the reference gene for mRNA and miRNA precursor normalization in leaf tissue samples; 172a or 393a as the reference miRNA for mature miRNA normalization in leaf tissue samples; *TuB* as the reference gene for mRNA and miRNA precursor normalization in root tissue samples; 172a as the reference miRNA for mature miRNA normalization in root tissue samples. These results indicated that the use of inappropriate reference genes may lead to inaccurate results.

## Discussion

RT-qPCR has become the preferred method for determining gene expression levels because it is more sensitive, simpler, and less time-consuming than approaches such as northern blotting, microarrays, and deep sequencing[5–7]. Normalization using reference genes eliminates sampling differences to enable the identification of gene-specific variations and is an essential component of a reliable RT-qPCR assay[16]. For an ideal reference gene, the gene expression variations among tested samples should be minimal or none [18, 19]. However, such genes are difficult to identify because plant gene expression is affected by environmental conditions[20–24]. In accordance with the MIQE, the reference genes for RT-qPCR assays should be identified and selected using specific experimental protocols [16].

Reference genes in soybean have been evaluated after exposure to some abiotic stresses in other studies. However, the current study differs from previous investigations because one of our objectives was to identify more stable reference genes for data normalization of samples exposed to specific experimental conditions. As Kulcheski et al. (2010)[36] reported, miR156b and miR1520d are stable reference miRNA in different soybean tissues and genotypes treated with abiotic or biotic stresses. In our study, miR1520d expression was unstable in leaf and root samples. Our observations indicated that 166a in leaf samples and 167a in root samples are the most suitable reference miRNA for mature miRNA data normalization. This inconsistency in results may be due to differences in cultivars, developmental stages, tissues, abiotic stress conditions, and treatment methods causing changes to miRNA expression levels. As Le et al. (2012) [35] reported, the most stable reference genes in soybean root samples treated with four abiotic stresses [dehydration, salinity, cold, and ABA (n = 9)] were *ABC*, *60s*, and *EF1b*. According to our findings, the three most stable reference genes in soybean root samples treated with various abiotic stress conditions (PEG-simulated drought, salinity, alkalinity, salinity+alkalinit, and abscisic acid) were *EF1b*, *EF1a*, and *60s*. The abiotic stresses used in these two studies were similar, but we used different treatment methods. Additionally, we investigated the effects of alkalinity and salinity+alkalinity, which differed from other related studies. Similar to other reports, we identified *60s* and *EF1b* as suitable reference genes, which inspired confidence when they normalized in root samples treated with abiotic stresses. Ma et al. (2013)[37] reported that *EF1b* and *UKN2* were the two most reliable reference genes for soybean leaf tissue (‘*Jidou 7*’ and ‘*Nannong 1138–2*’) treated with various stresses (i.e., salt, drought, darkness, and soybean mosaic virus infection). However, it is contradictory, the results of Ma et al. (2013)[37] also showed *EF1b* and *UKN2* expression levels are not stable enough in soybean leaf tissue exposed to drought stress. In our study, we identified *Act* as the most stable reference gene in soybean leaf samples exposed to PEG-simulated drought, salinity, alkalinity, salinity +alkalinity, and ABA.

In conclusion, we evaluated the expression stability of eight candidate reference mRNAs and eight candidate reference miRNAs for use in the normalization of expression level data for mature miRNAs, miRNA precursors, and mRNAs in soybean leaf and root samples treated with various abiotic stresses. The results of ΔCq analysis (Fig 1) and geNorm anlysis (Figs 2 and 3) indicated the intra-tissue candidate reference mRNA/miRNA expression was more stable than the inter-tissue expression. Especially, we found candidate reference miRNAs unlike tanditional housekeeping gene, there weren’t reference miRNA or reference miRNA combination stable enough for data normalization in leaf and root combined samples. This may be because of the differential expression of candidate reference mRNA/miRNAs between leaf and root samples. So we suggest to select reference mRNA/miRNA in leaf or root samples, not in leaf and root combined samples. In addition, it is proved that candidate reference miRNAs have some specific function[55–58], this may lead to expression variation related to its own function under some conditions. The selected reference mRNA/miRNAs may express differently in different experimental conditions, so it is recommended to keep the conditions used in this study. In contrast the stability of candidate reference mRNA/miRNAs has to be re-evaluated to adopt their own experimental conditions. Generally, We assessed the expression stability of candidate reference mRNA/miRNAs in our study according to the consensus rank orders determined by geNorm, NormFinder, and BestKeeper algorithms in leaf or root samples (Table 2). We recommended that *Act* is the most suitable reference gene in leaf samples and *EF1b* in root samples for mRNA and miRNA precursor normalization. The most suitable reference is l66a in leaf samples and 167a in root samples for mature miRNA normalization. Furthermore, the use of multiple combinations of reference for data normalization can improve the reliability of RT-qPCR results, but it is time-consuming and more expensive. We also suggest to consider the accuracy and cost of reference combinations to be used. The best combination of reference genes for mRNA and miRNA precursor normalization were *EF1a* + *Act* (geNorm) and *EF1b* + *Act* (NormFinder) for leaf samples, and *EF1a + EF1b* (geNorm) and *60s* + *EF1b* (NormFinder) for root samples. The best combination of reference for mature miRNA normalization were 166a + 167a (geNorm or NormFinder) in leaf samples, and 171a + 156a (geNorm) and 167a + 171a (NormFinder) for root samples.

## Supporting Information

S1 Fig Quality assessment of random RNA samples. (A) 1% agarose gel electrophoresis was used to check RNA integrity. The ribosomal RNA bands are clearly visible and the 25S:18S ratio was approximately 2:1, which indicated that the RNA was intact. (B) RNA purity was determined using a NanoDrop spectrophotometer. A 260 nm/280 nm optical density ratio ranging from 1.8 to 2.0 indicated high quality RNA. (A and B) Samples were randomly selected from the various abiotic stress samples used for RT-qPCR (see S1 Table).
(TIF)

S1 File Primer pair amplification specificities for RT-qPCR. (DOC)

S2 File Primer pair annealing locations on their respective transcripts. (DOC)

S3 File NormFinder analysis. (DOC)

S4 File BestKeeper analysis. (DOC)

S1 Table Description of the samples used for RT-qPCR. (DOC)
