# Supplementary figures and images for: Normalization for Relative Quantification of mRNA and microRNA in Soybean Exposed to Various Abiotic Stresses
Source: PLoS One. 2016 May 13;11(5):e0155606. doi: 10.1371/journal.pone.0155606 (PMC4866712; doi:10.1371/journal.pone.0155606)

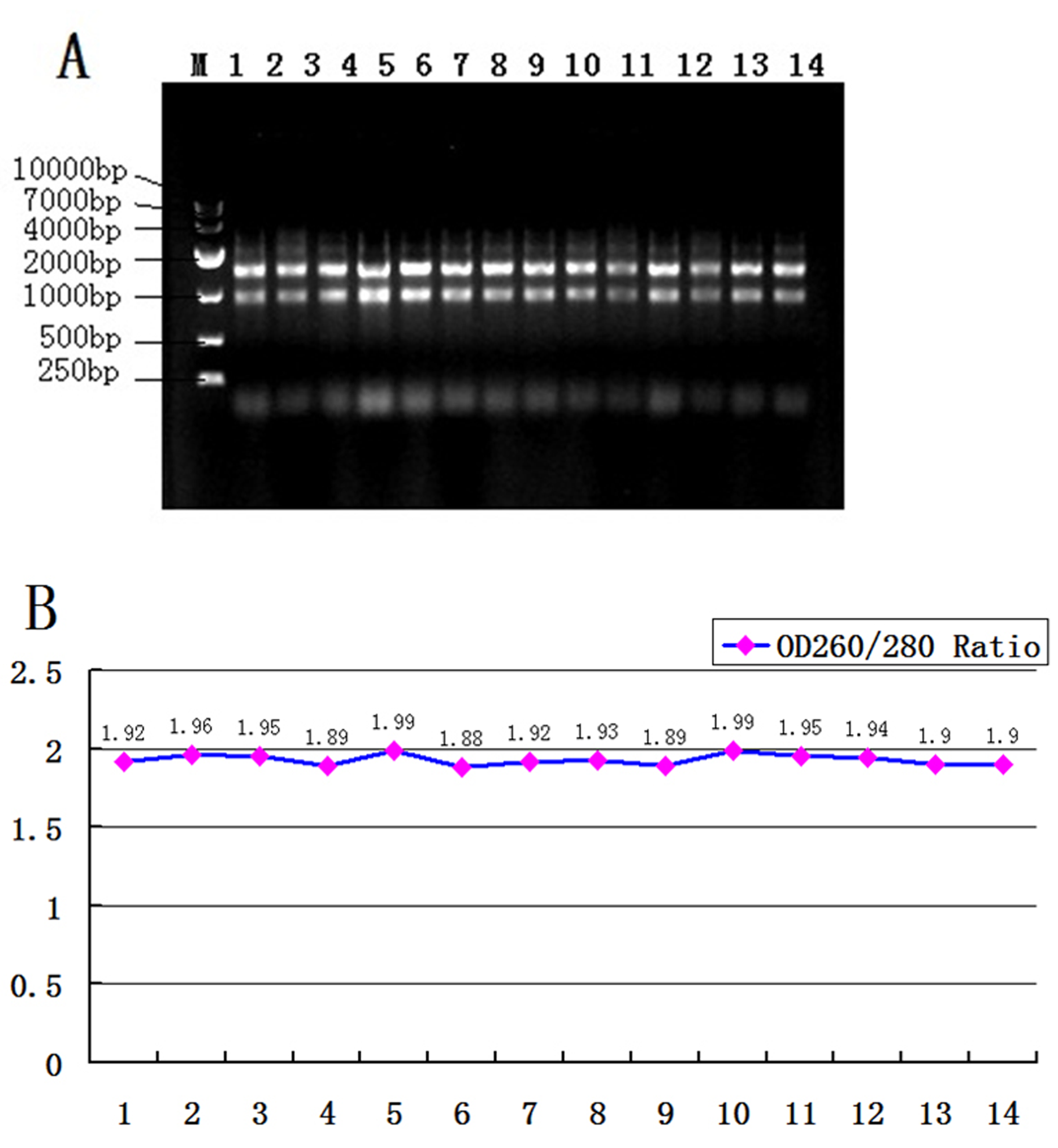

Supplement: S1 Fig — (A) 1% agarose gel electrophoresis was used to check RNA integrity. The ribosomal RNA bands are clearly visible and the 25S:18S ratio was approximately 2:1, which indicated that the RNA was intact. (B) RNA purity was determined using a NanoDrop spectrophotometer. A 260 nm/280 nm optical density ratio ranging from 1.8 to 2.0 indicated high quality RNA. (A and B) Samples were randomly selected from the various abiotic stress samples used for RT-qPCR (see S1 Table). (TIF) [file pone.0155606.s001.tif]
