# Supplementary material for: Normalization for Relative Quantification of mRNA and microRNA in Soybean Exposed to Various Abiotic Stresses
Source: PLoS One. 2016 May 13;11(5):e0155606. doi: 10.1371/journal.pone.0155606 (PMC4866712; doi:10.1371/journal.pone.0155606)
Supplement: S1 File — (DOC) [file pone.0155606.s002.doc]

S1 File **Primer pair amplification specificities for RT-qPCR.**

| **symbol** | **Amplification plots** | **Dissociation curve** | **Standard curves** |
| --- | --- | --- | --- |
| **The amplification specificity of eight candidate reference mRNA genes.** | | | |
| ***Act*** | 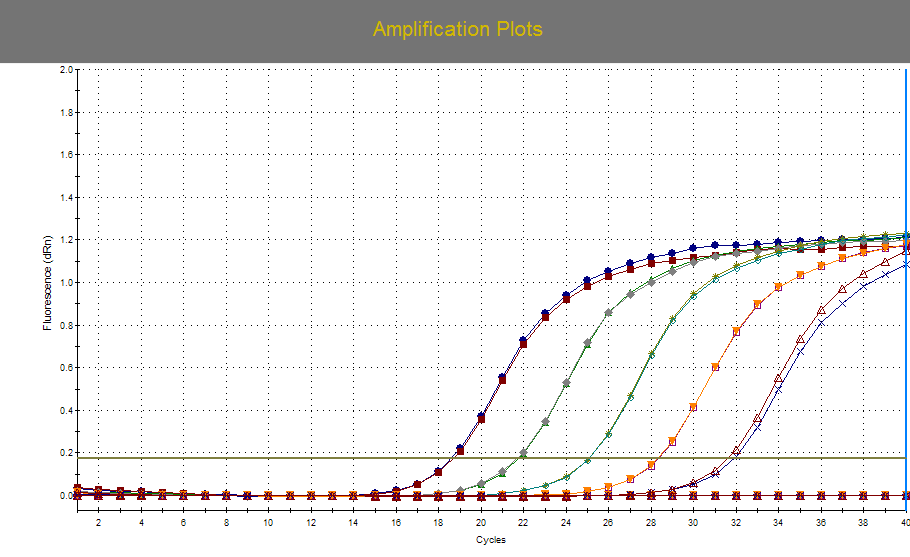 | 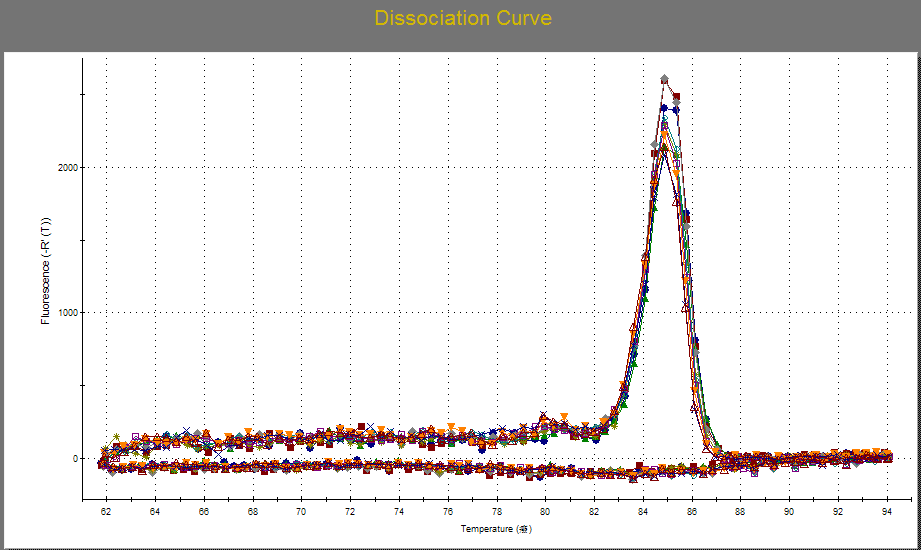 | 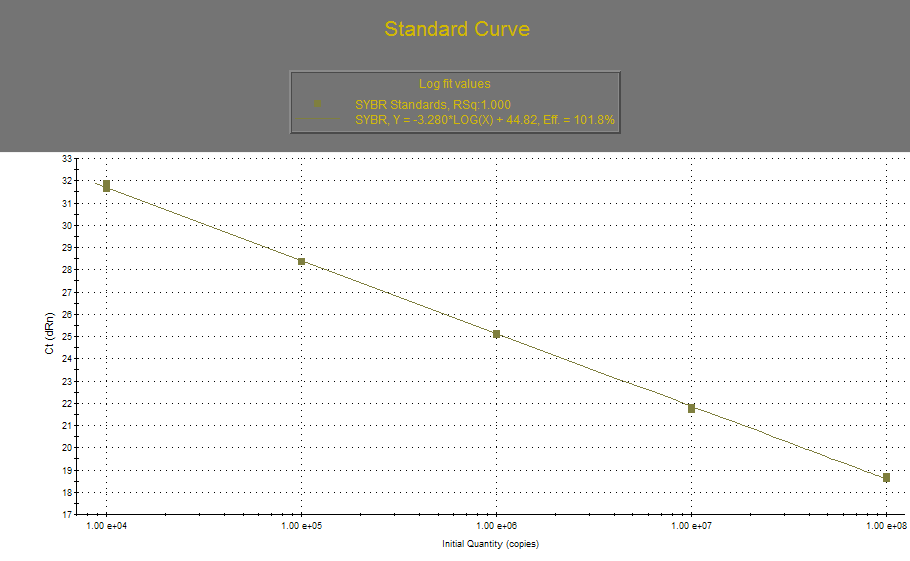 |
| ***Cyp*** | **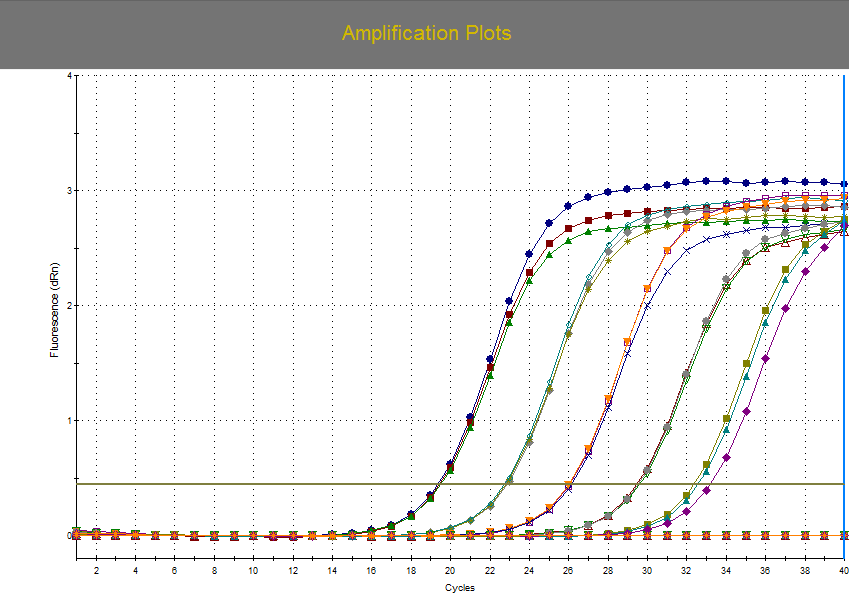** | **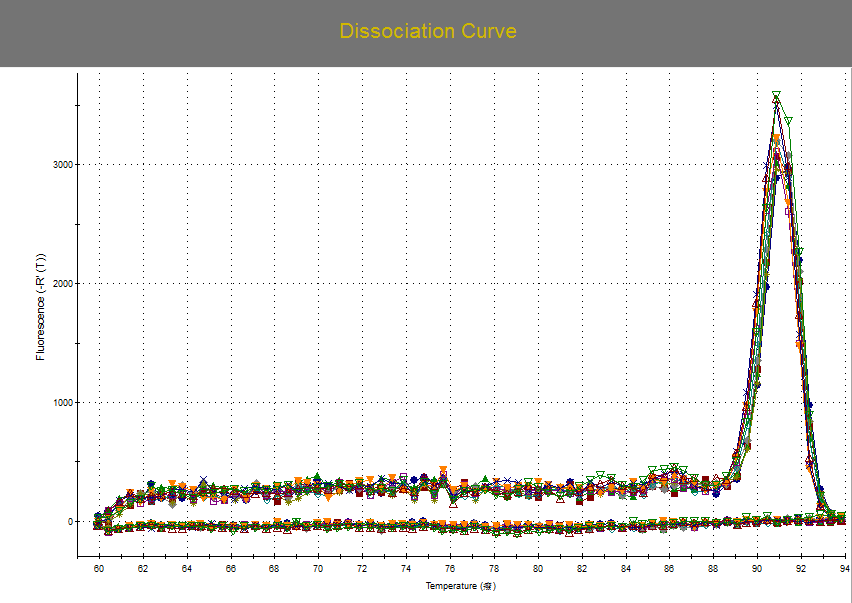** | **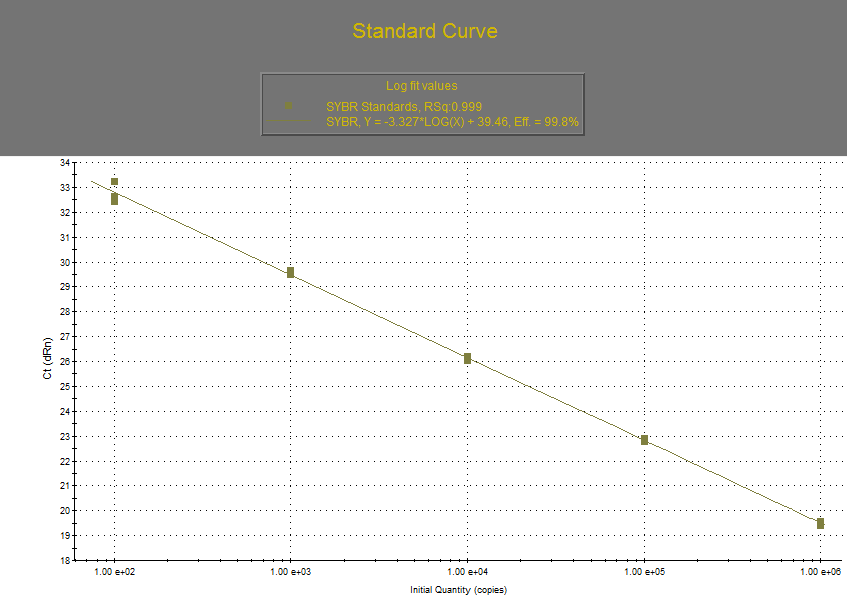** |
| ***EF1a*** | 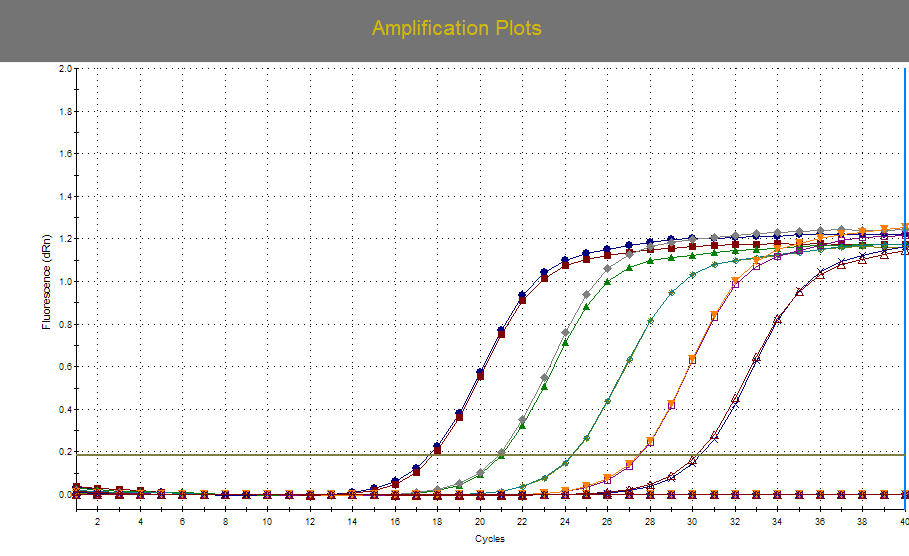 | 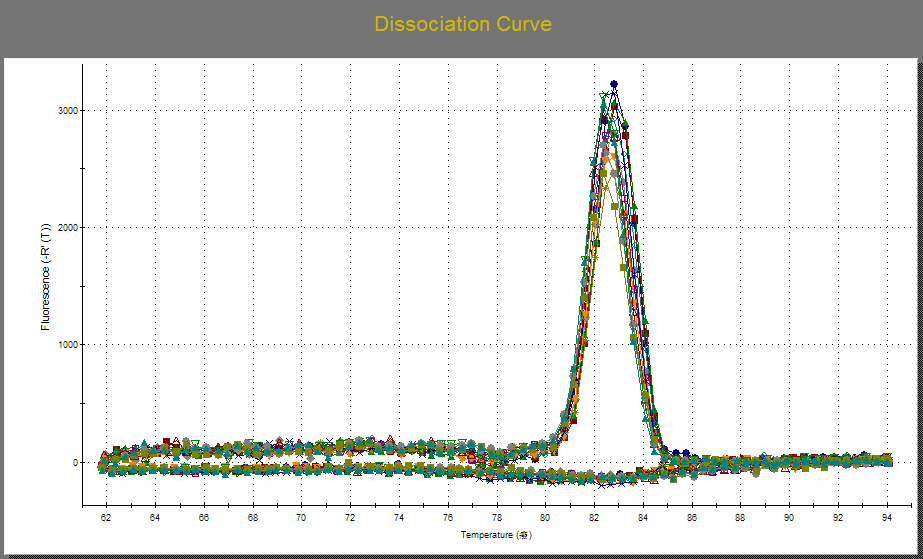 | 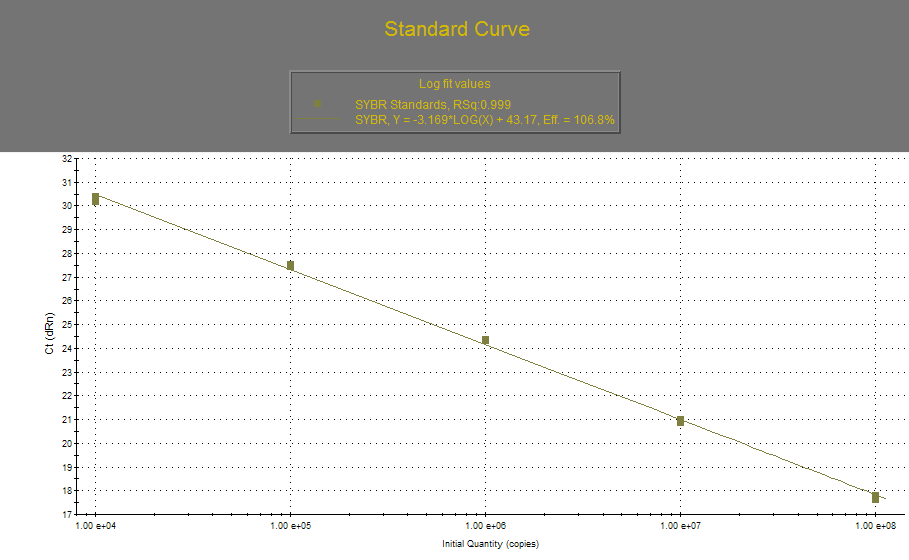 |
| ***EF1b*** | 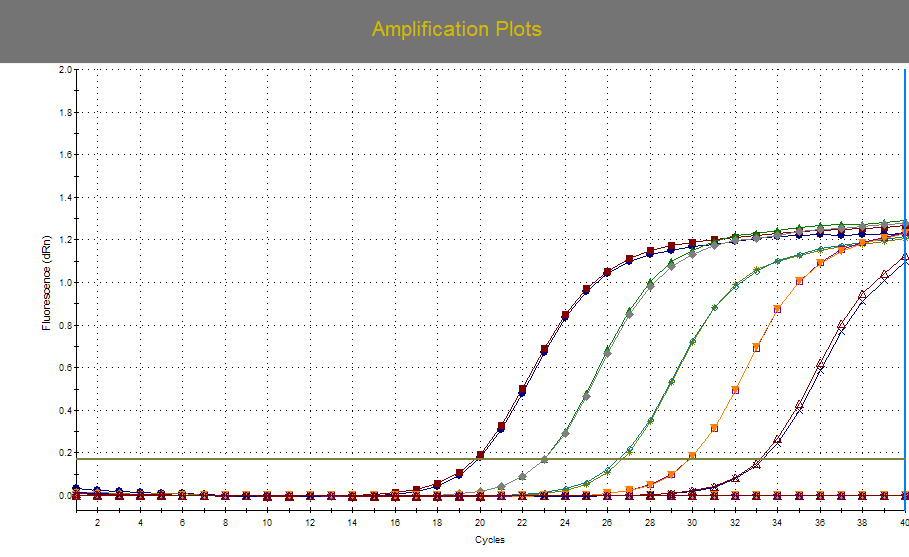 | 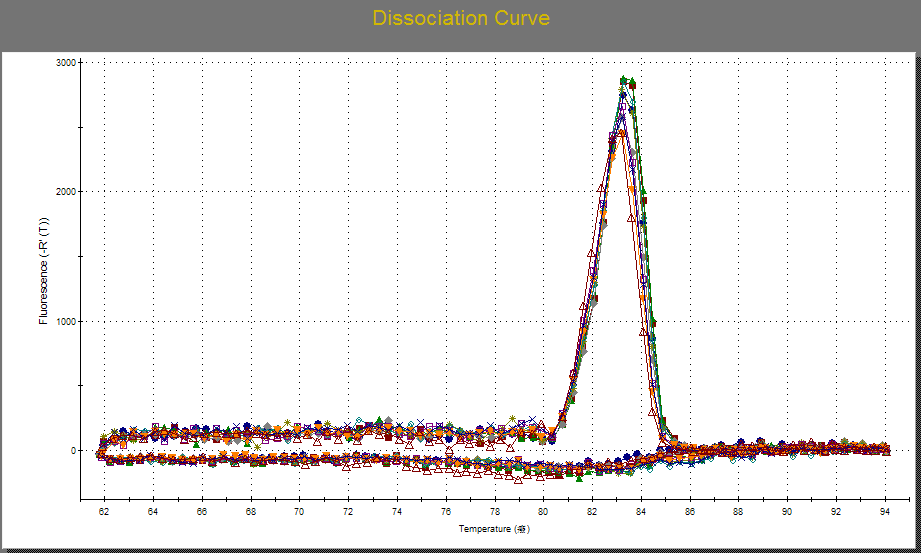 | 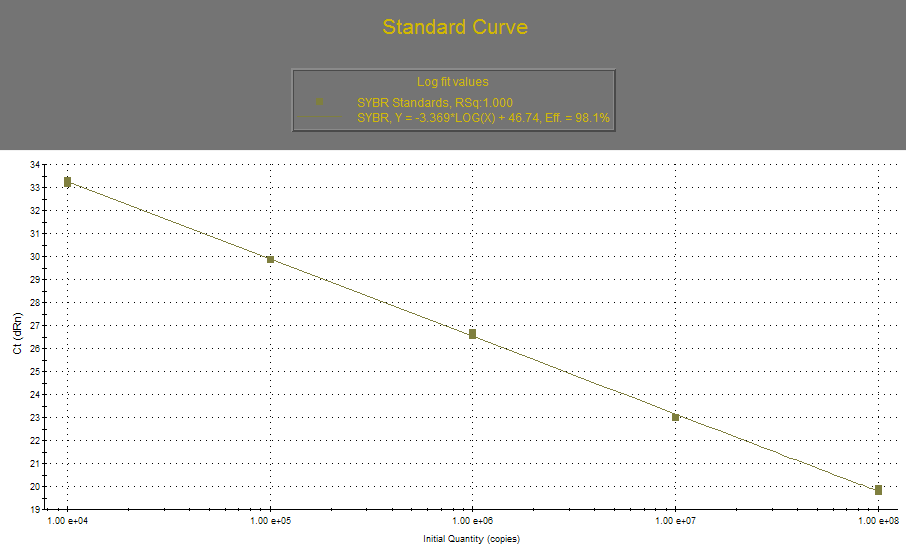 |
| ***Fbox*** | **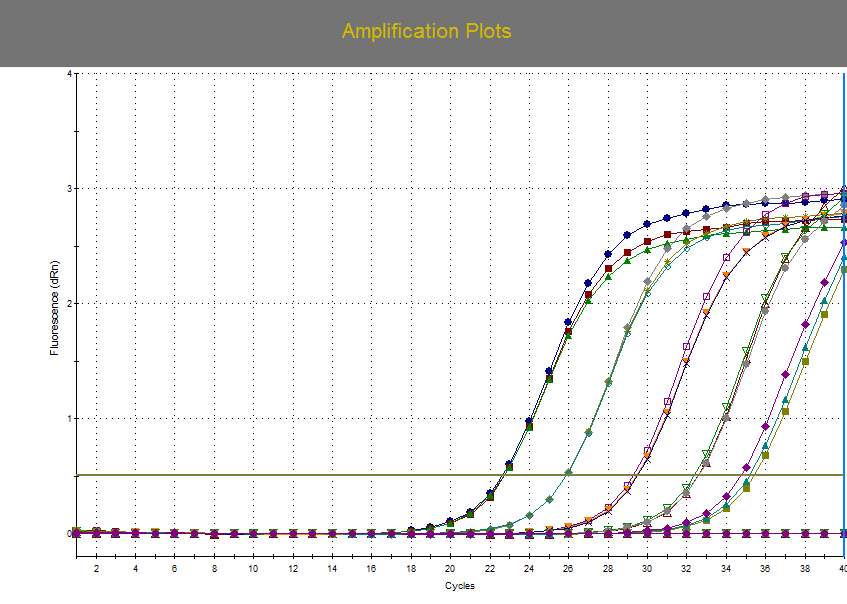** | **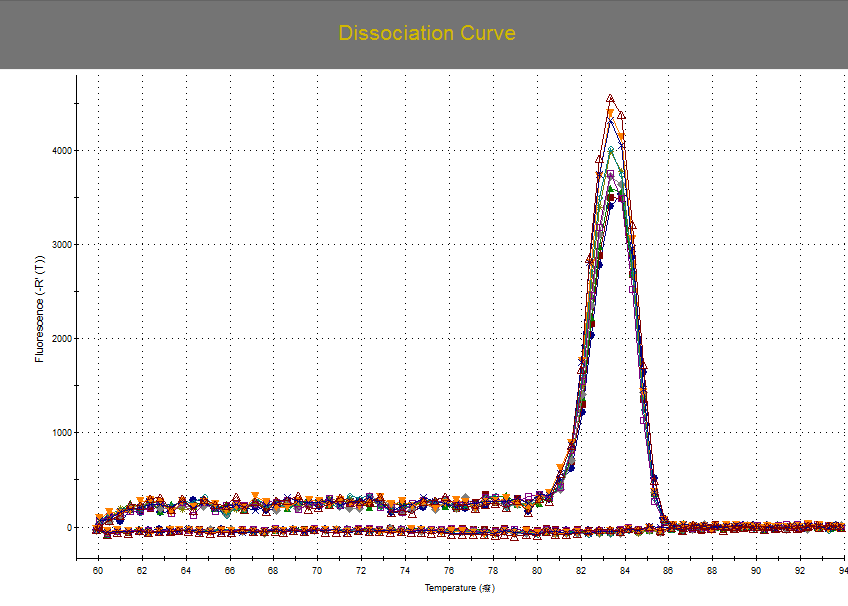** | **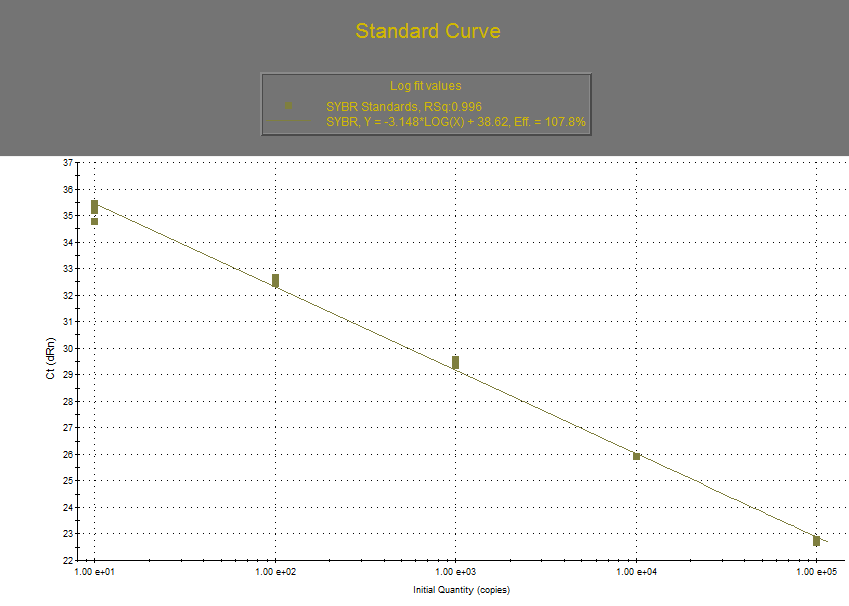** |
| ***TuA*** | **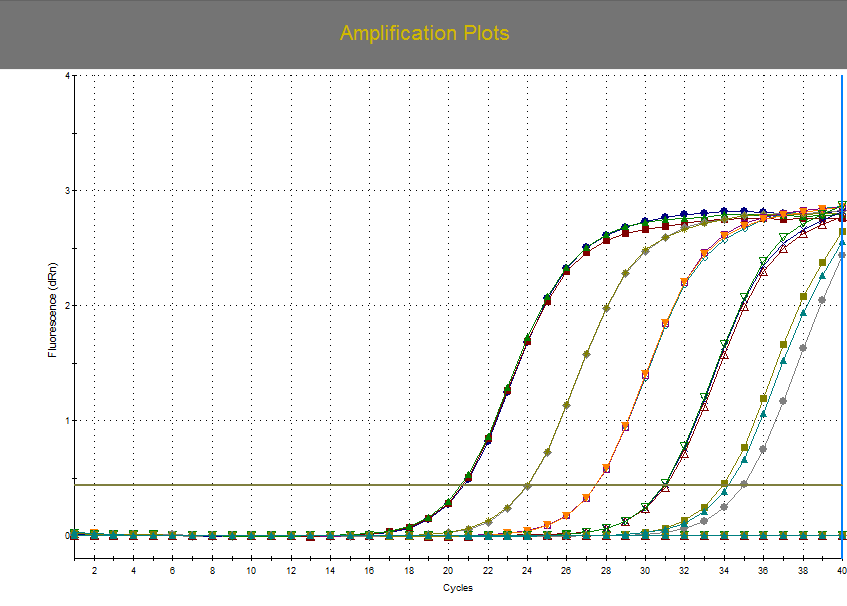** | **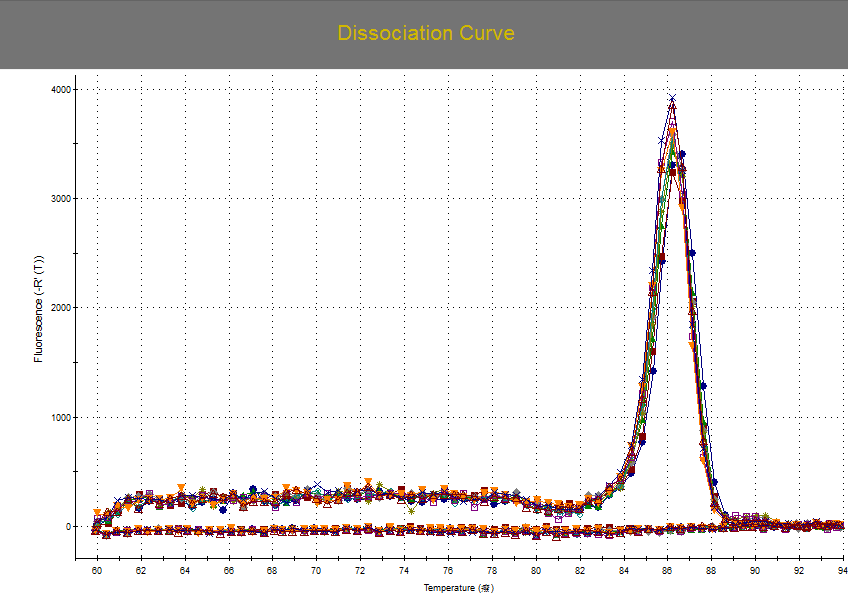** | **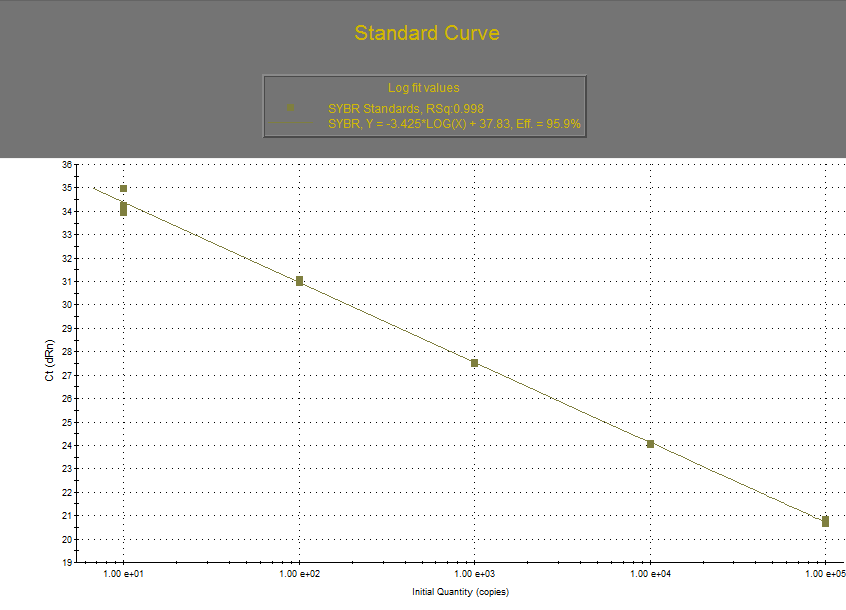** |
| ***TuB*** | **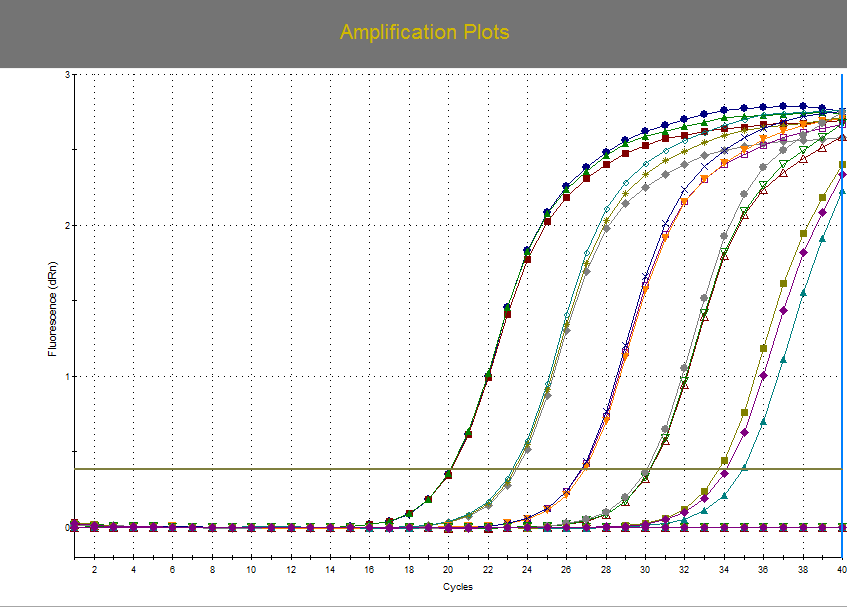** | **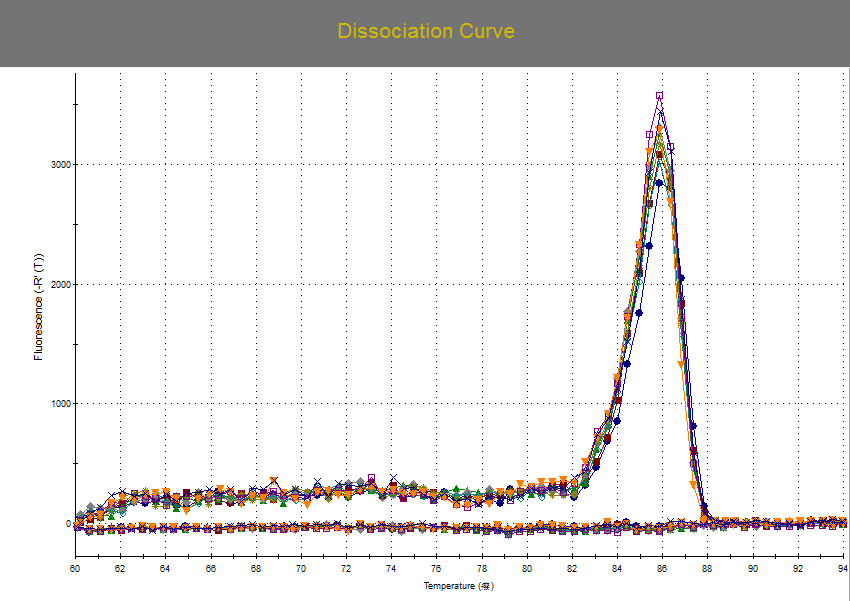** | **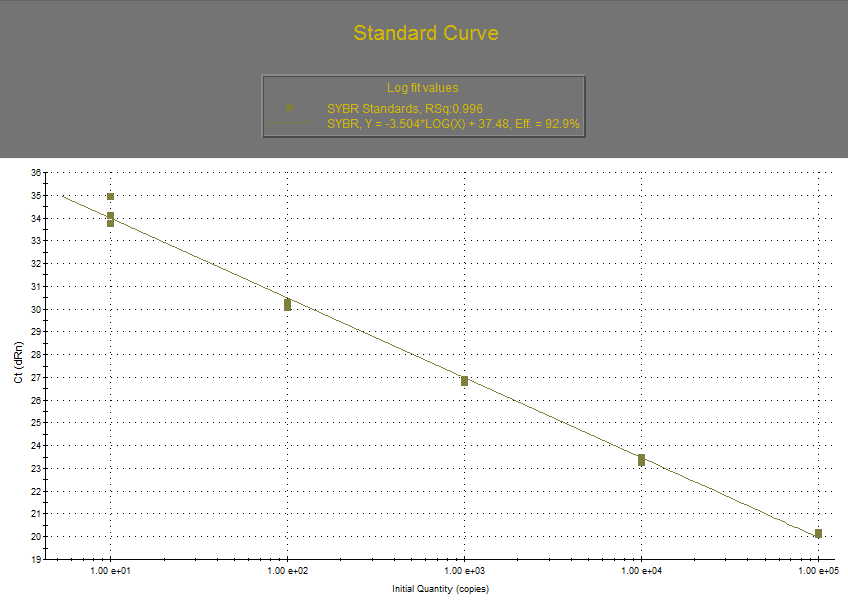** |
| ***60s*** | **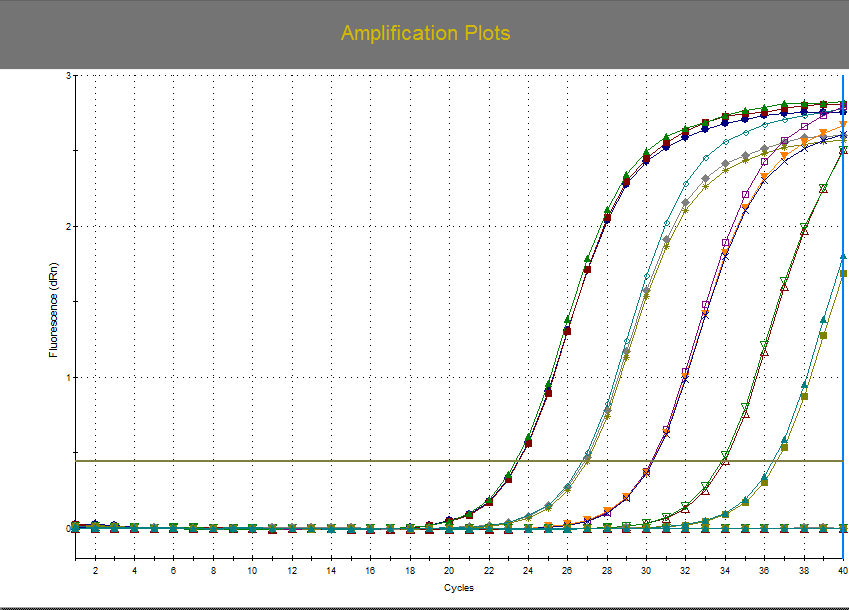** | **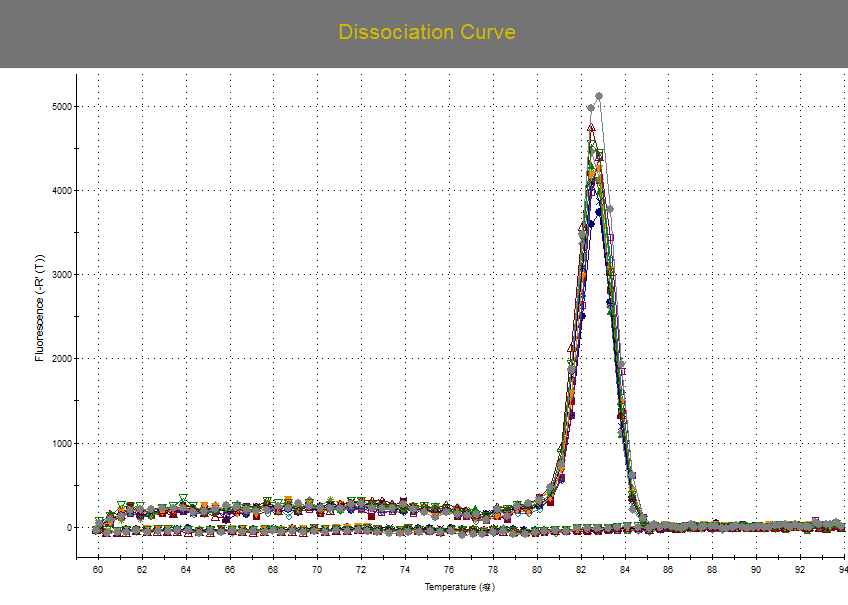** | **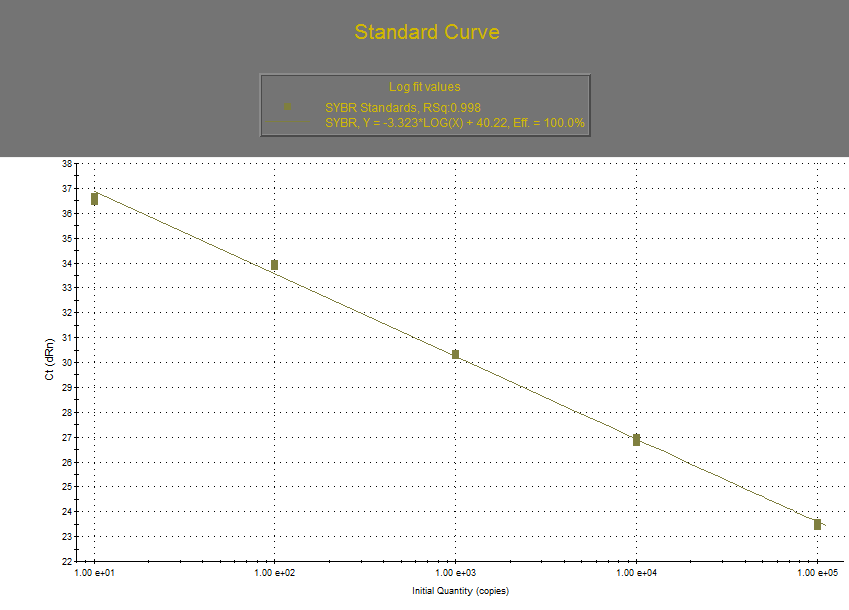** |
| **The amplification specificity of eight candidate reference miRNA.** | | | |
| ***156a*** | 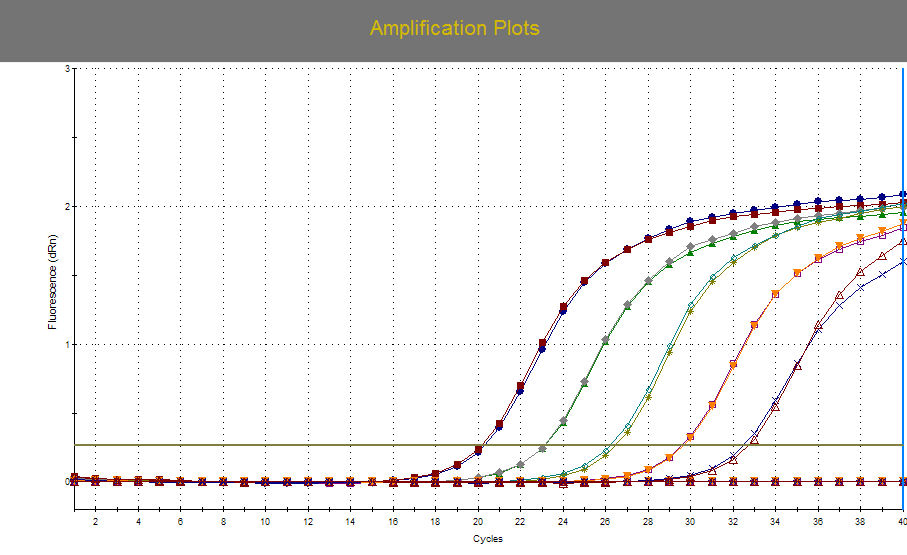 | 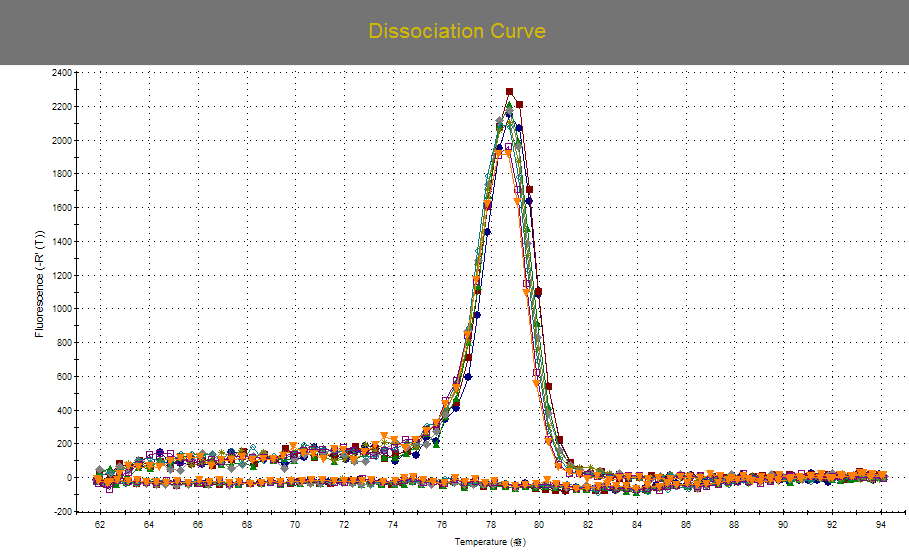 | 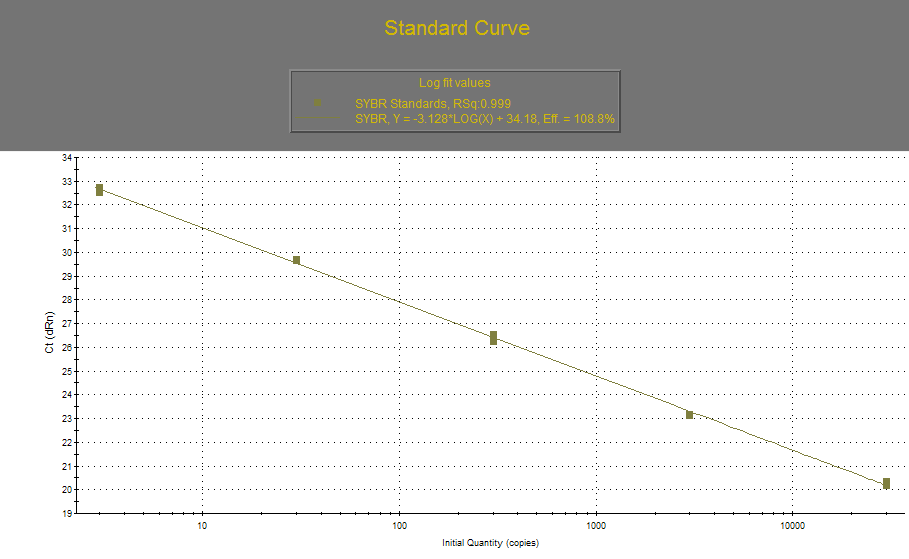 |
| ***166a*** | 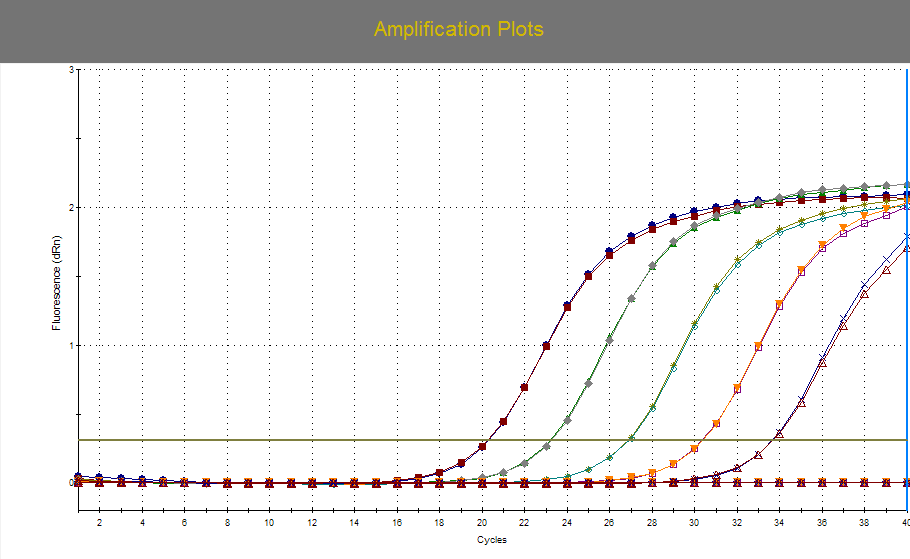 | 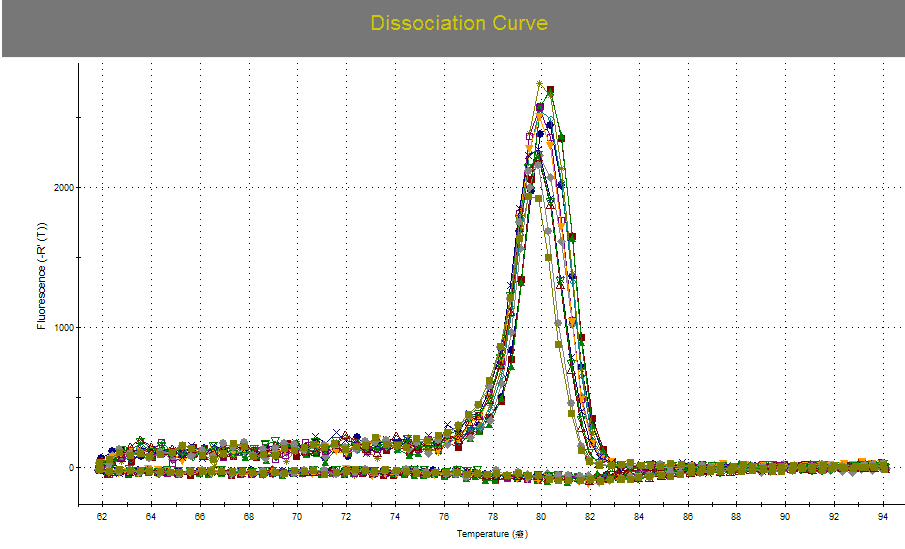 | 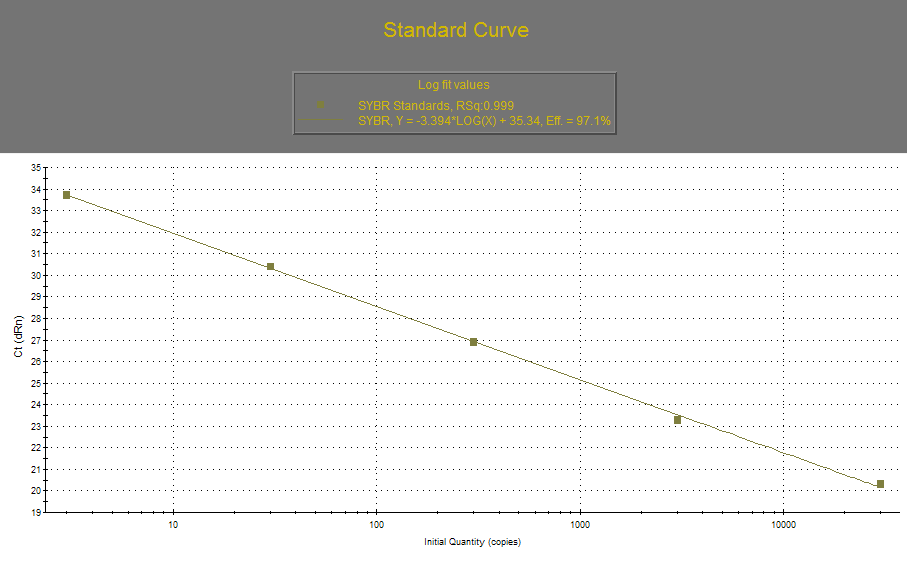 |
| ***167a*** | 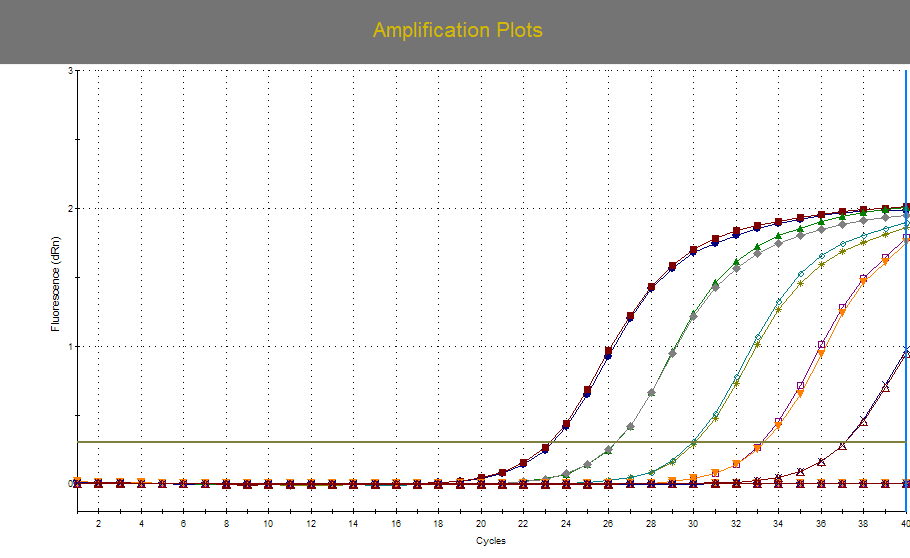 | 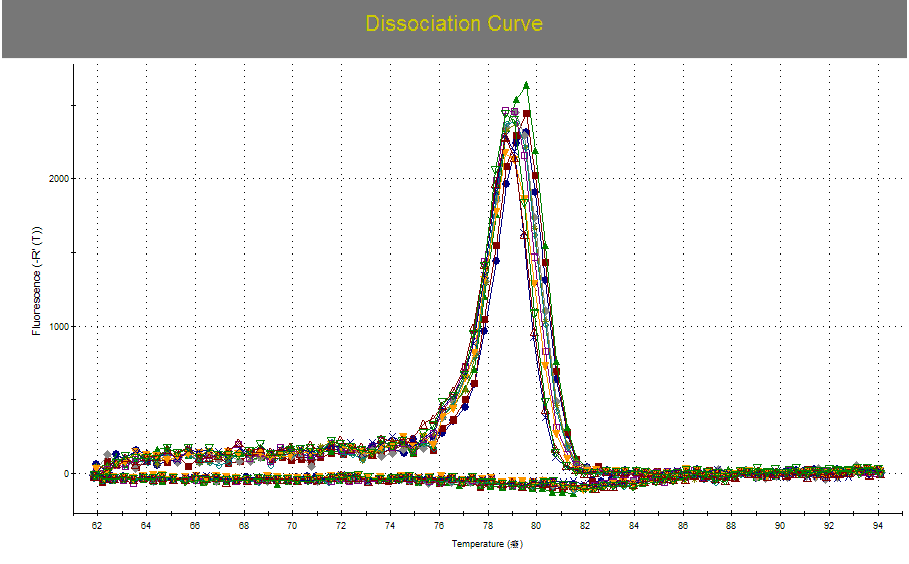 | 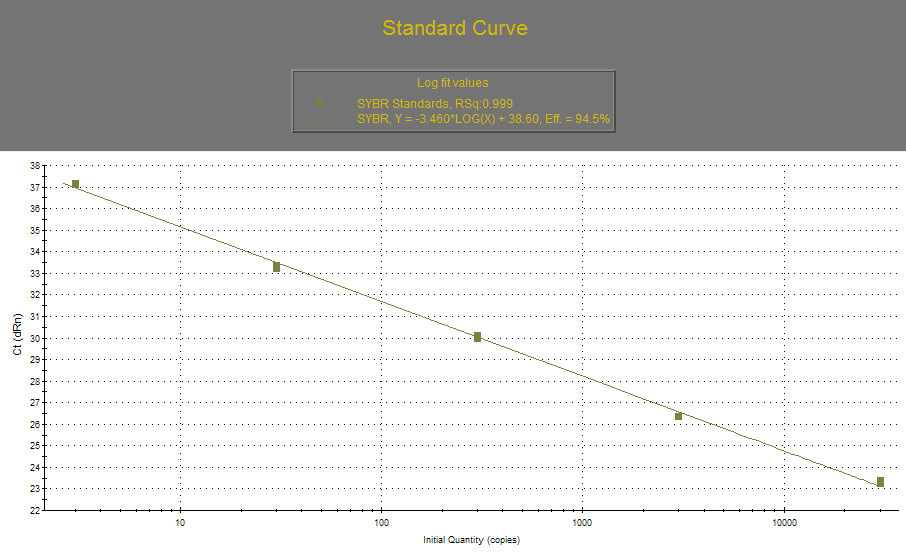 |
| ***171a*** | 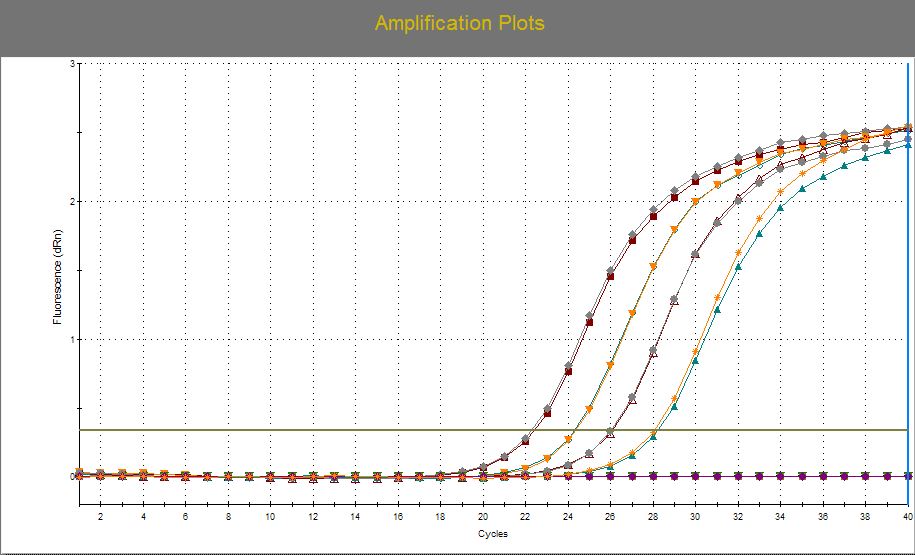 | 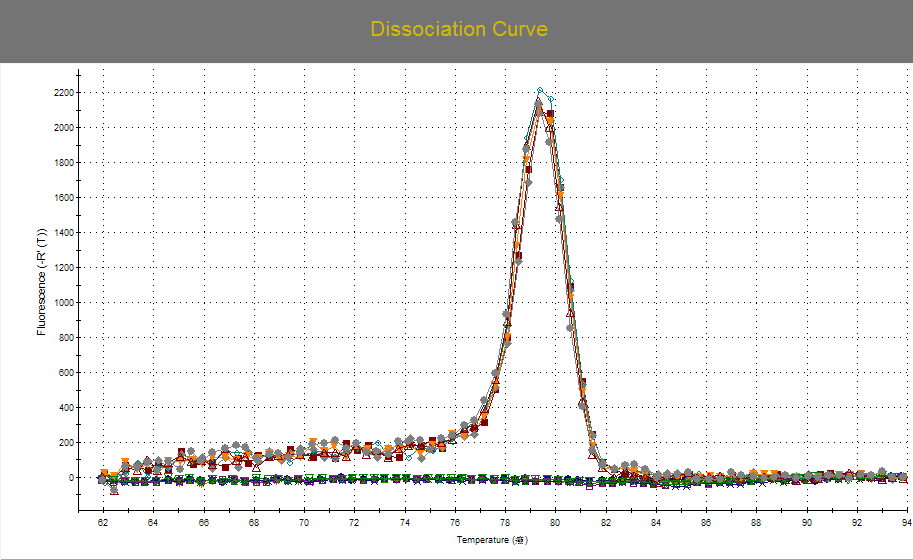 | 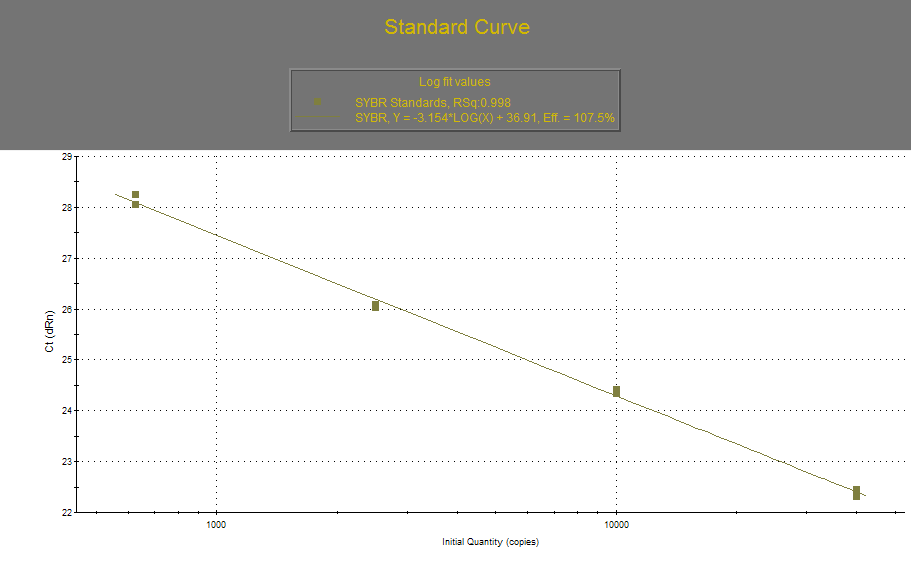 |
| ***172a*** | 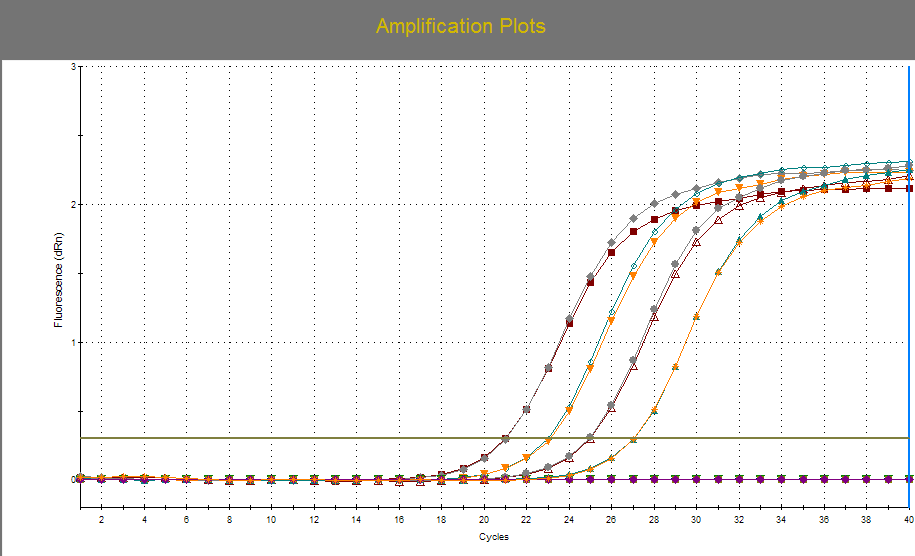 | 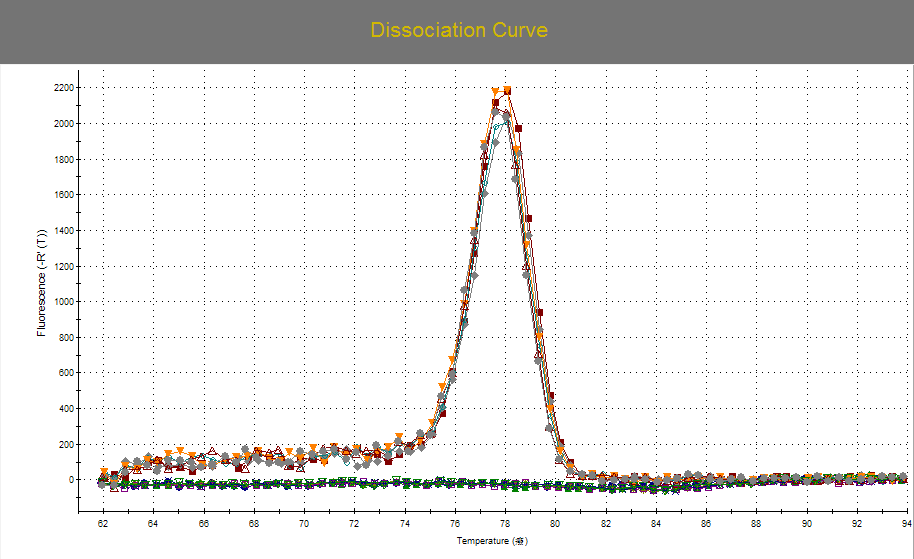 | 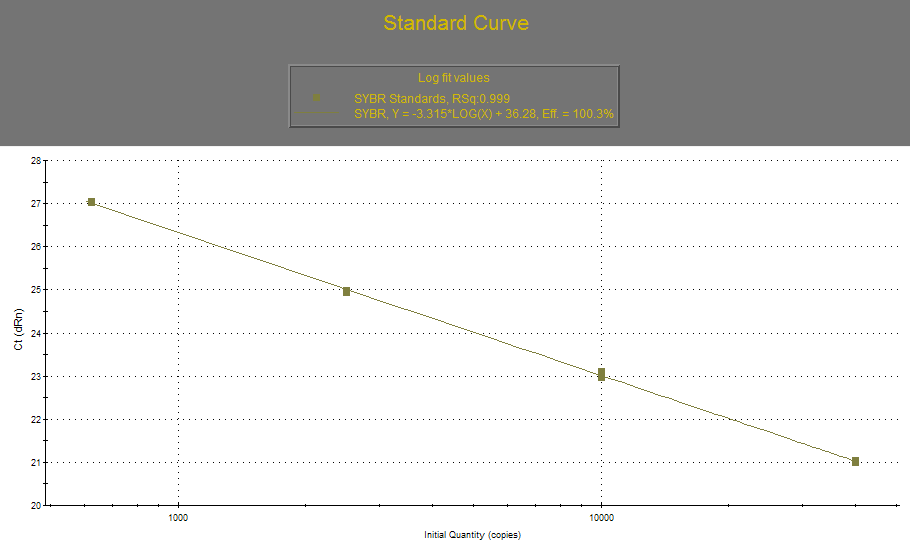 |
| ***393a*** | 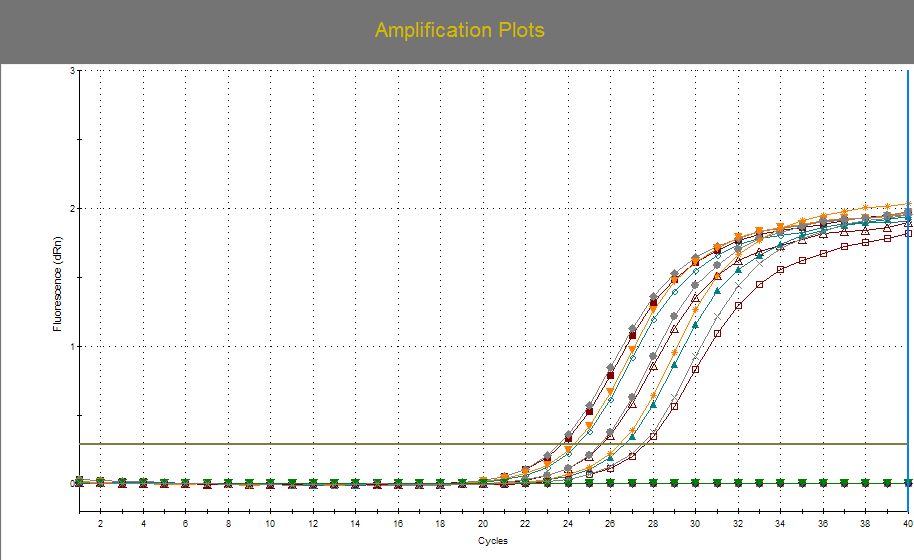 | 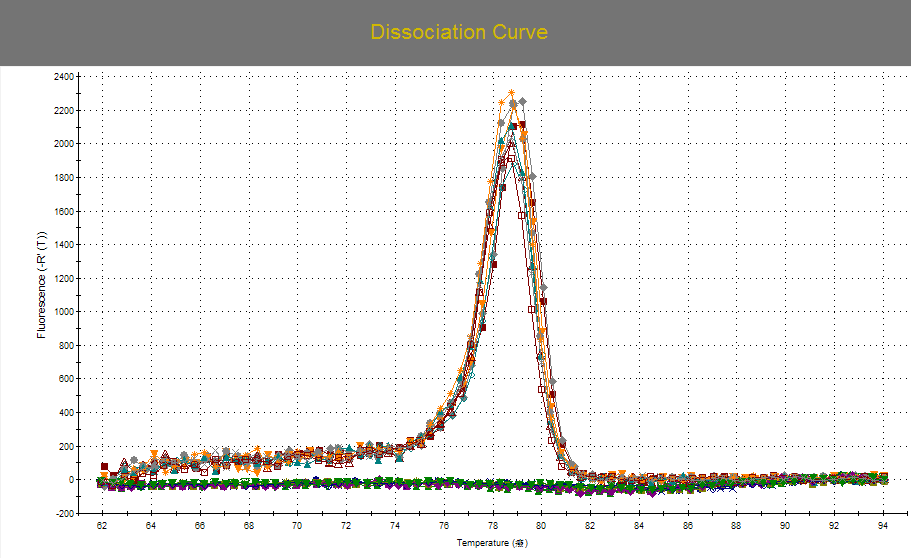 | 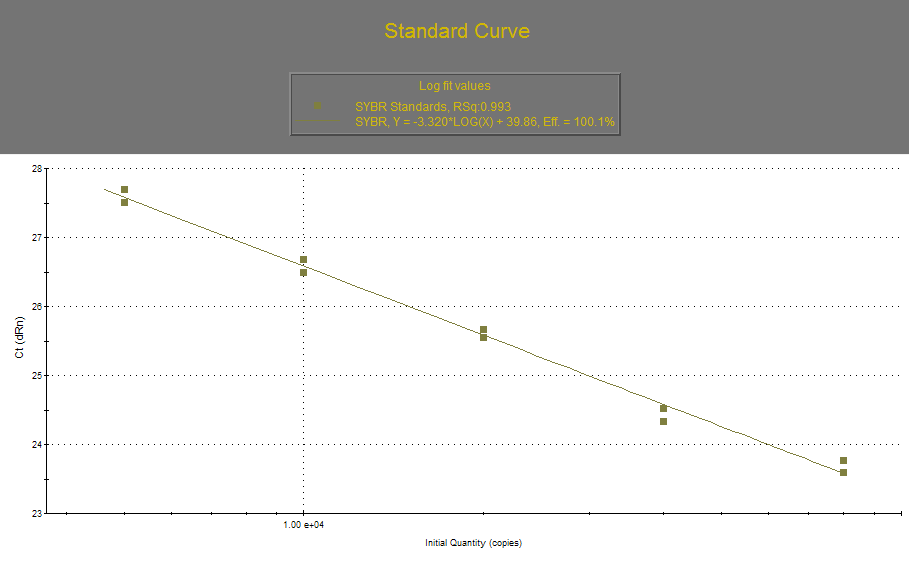 |
| ***397a*** | 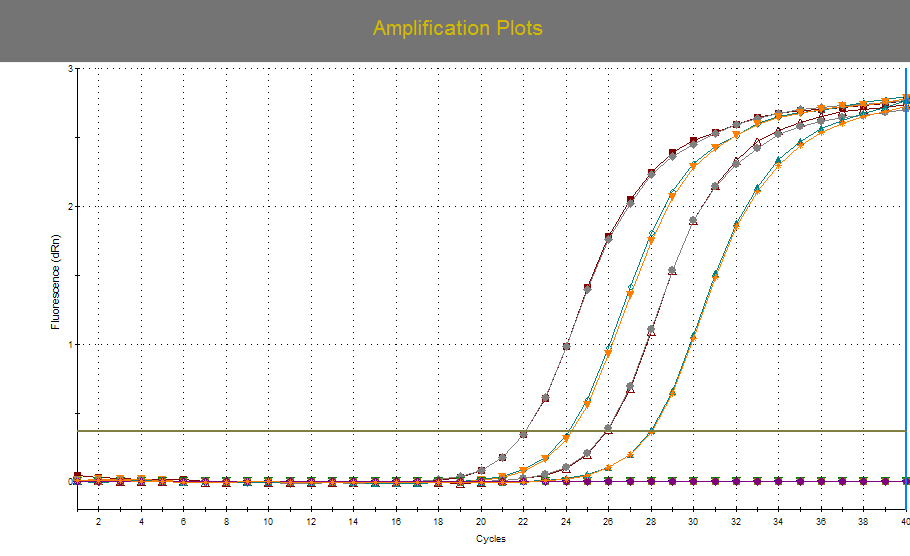 | 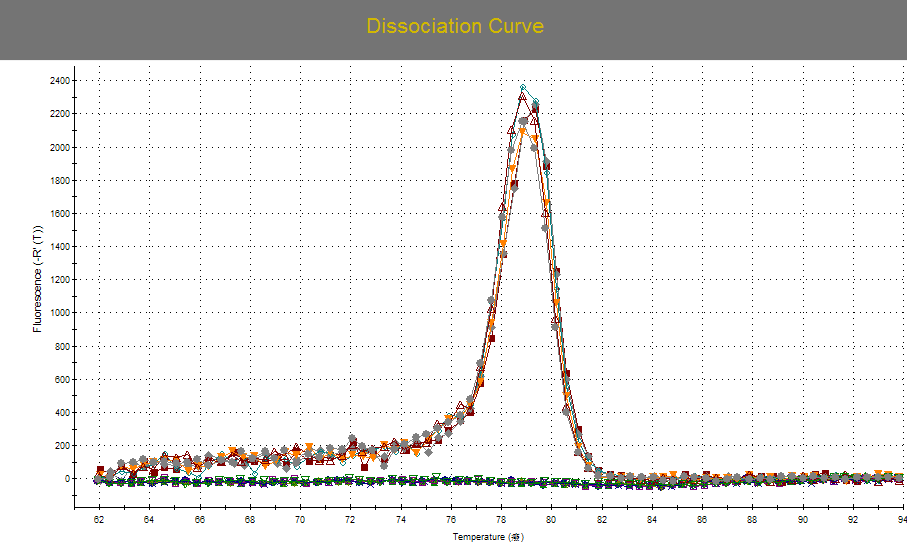 | 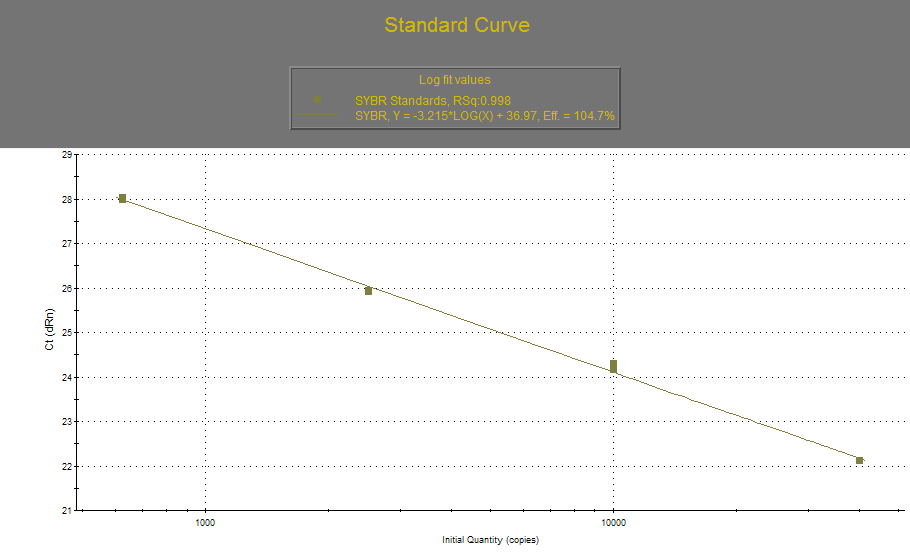 |
| ***1520d*** | **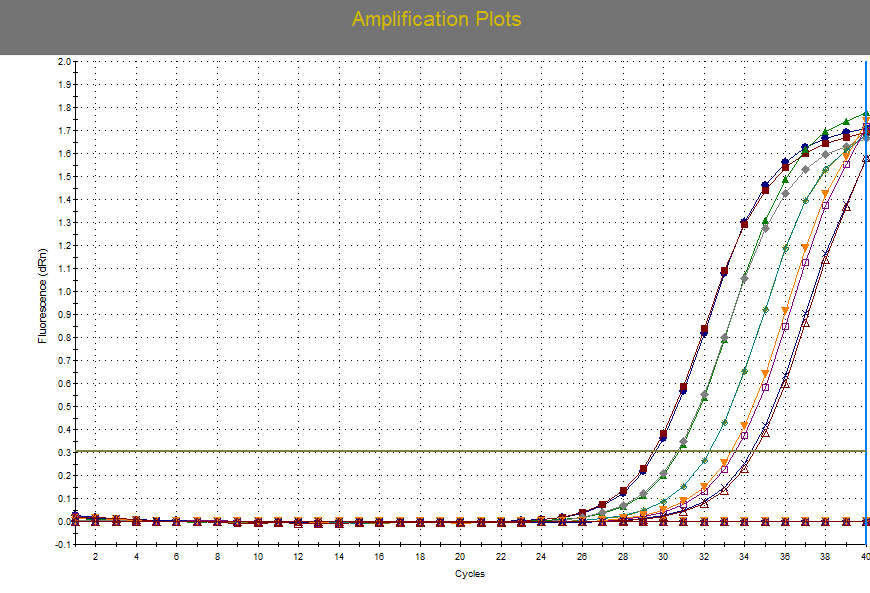** | 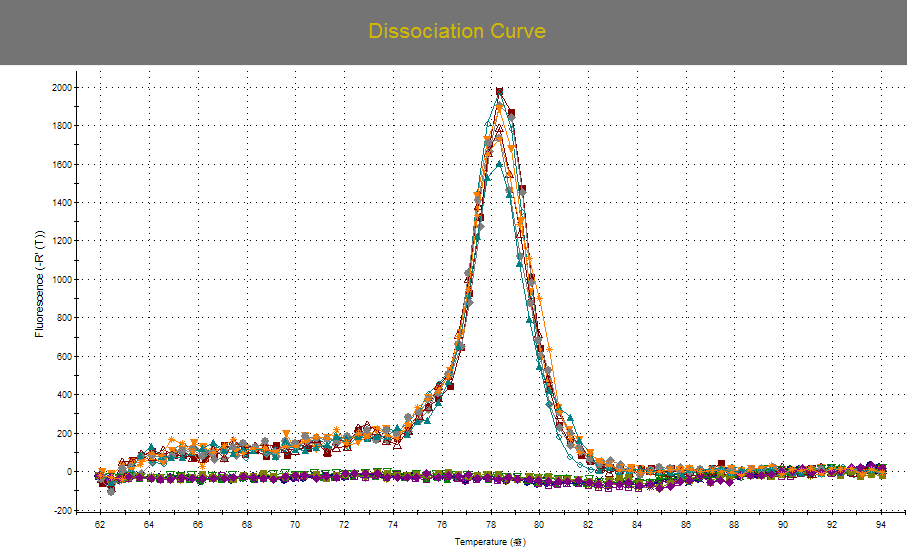 | **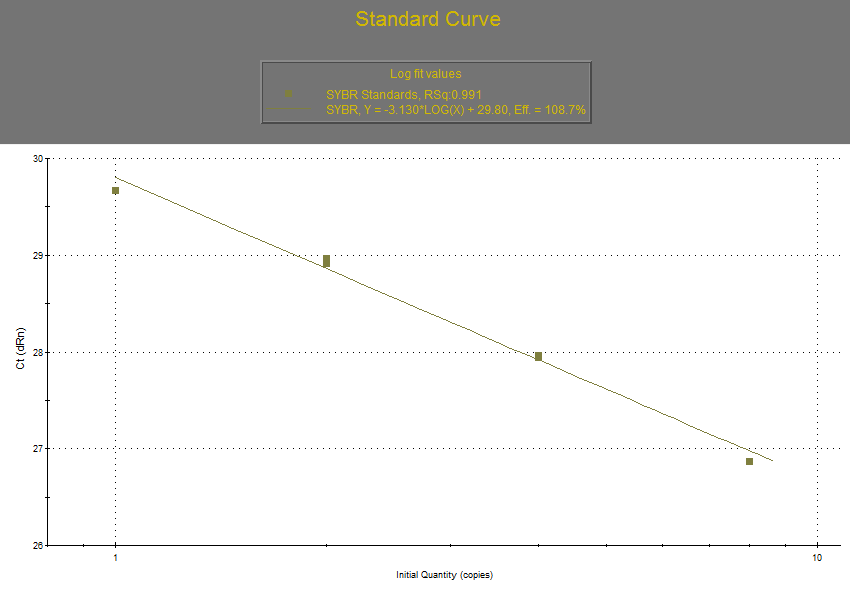** |
| **The amplification specificity of the interested genes.** | | | |
| ***396a*** | **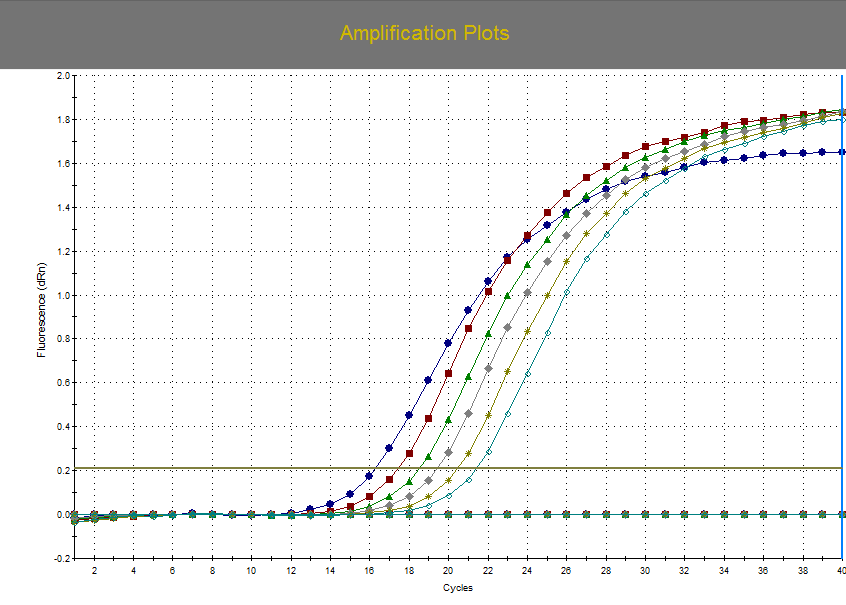** | **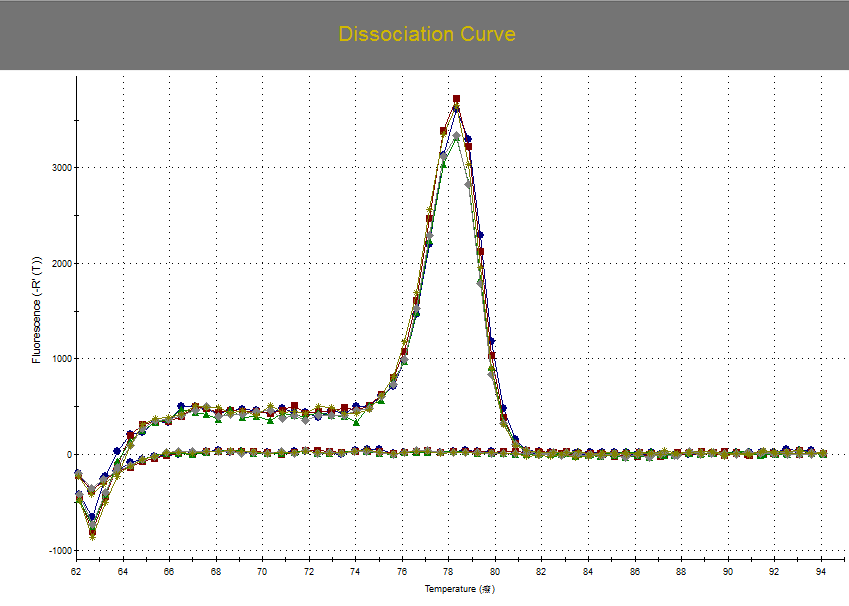** | **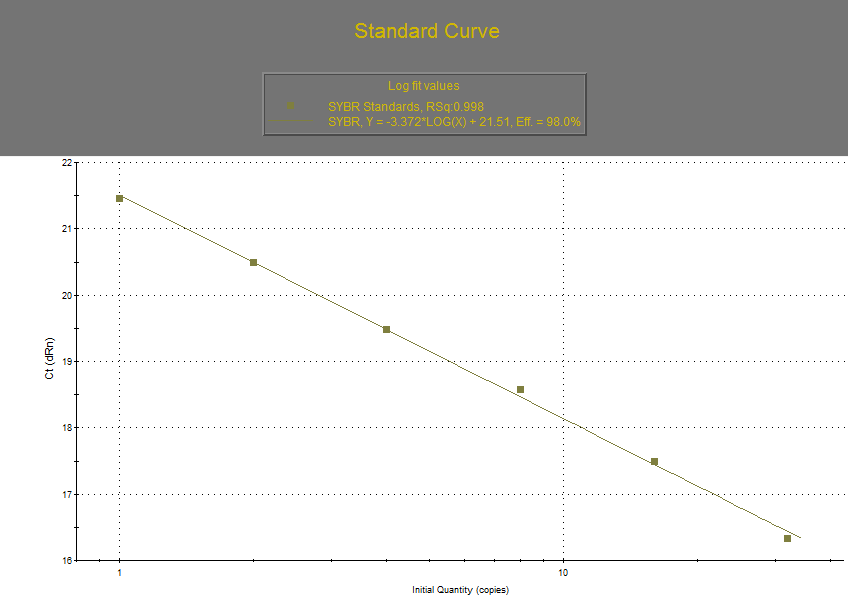** |
| ***Pre-396a*** | **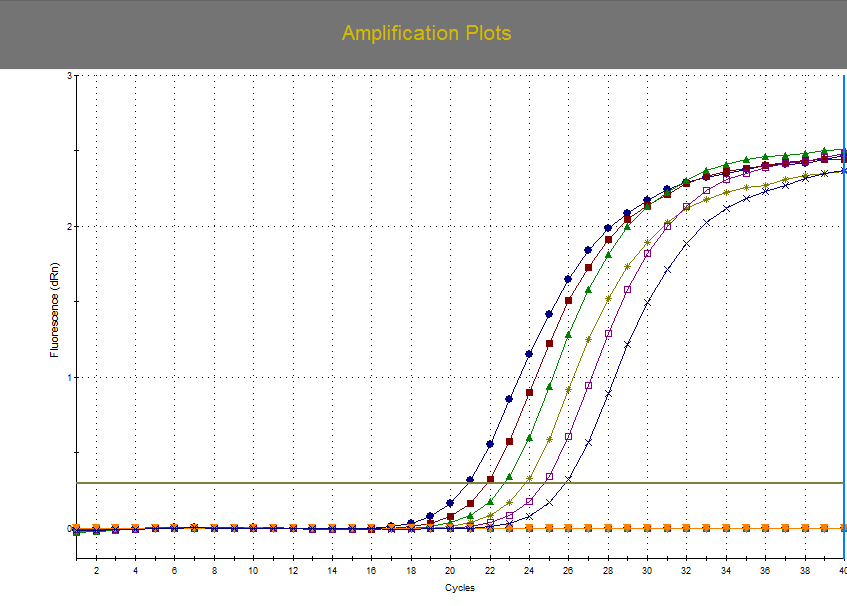** | **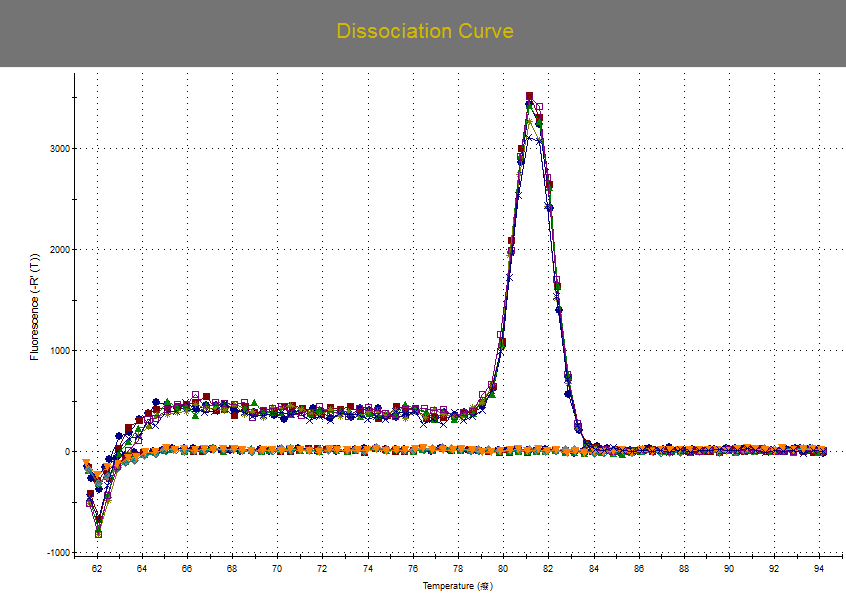** | **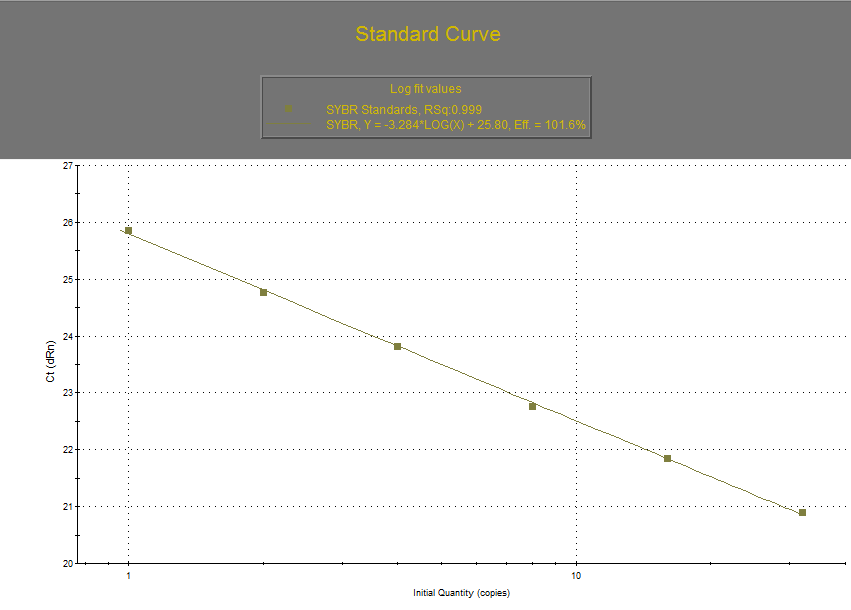** |
| ***GRF9*** | **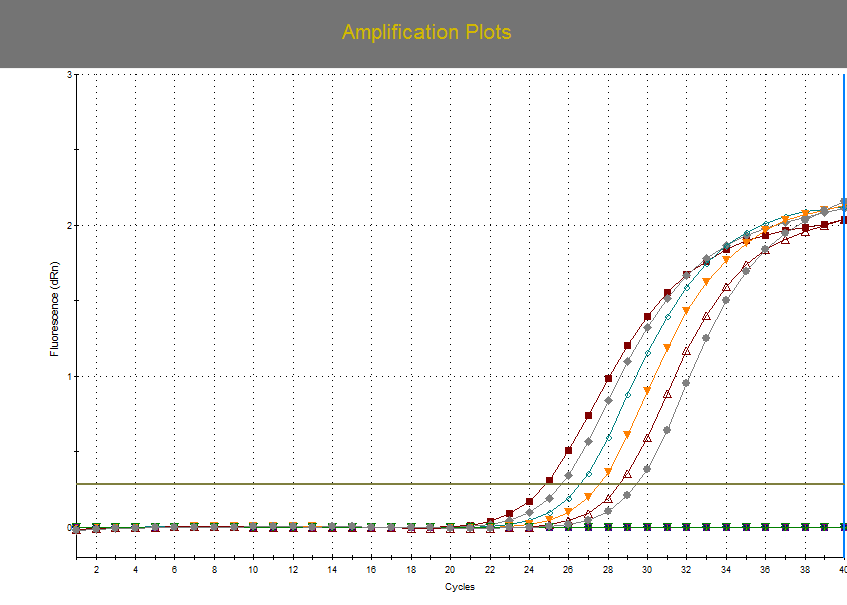** | **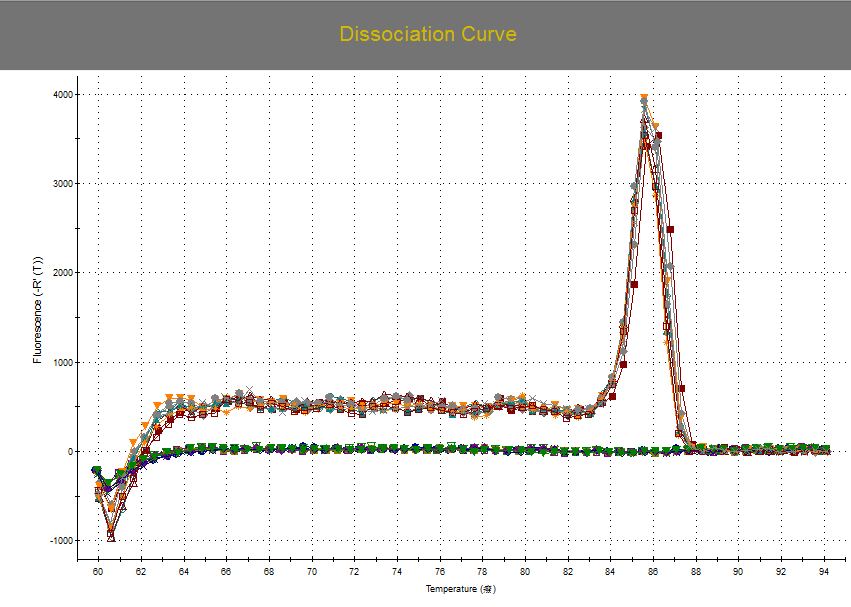** | **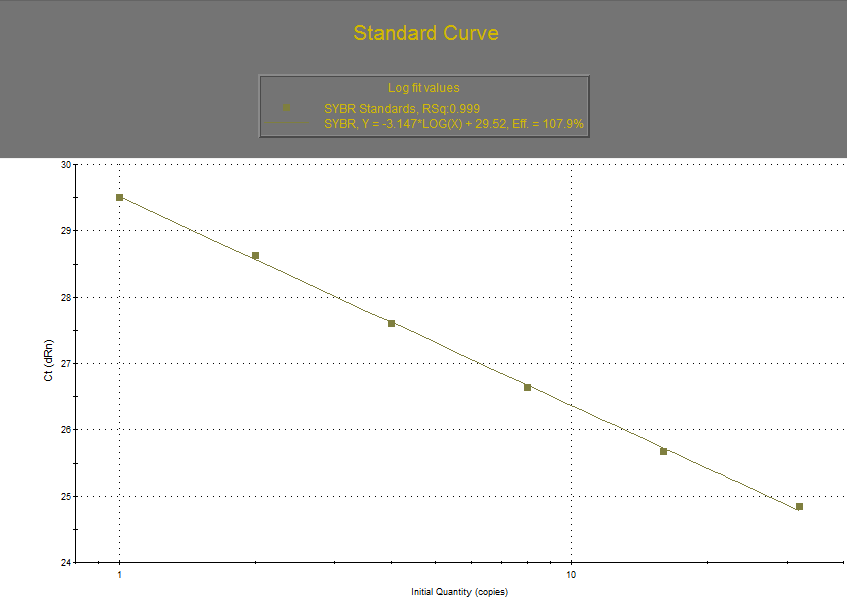** |
