# Supplementary material for: Normalization for Relative Quantification of mRNA and microRNA in Soybean Exposed to Various Abiotic Stresses
Source: PLoS One. 2016 May 13;11(5):e0155606. doi: 10.1371/journal.pone.0155606 (PMC4866712; doi:10.1371/journal.pone.0155606)
Supplement: S2 File — (DOC) [file pone.0155606.s003.doc]

**S2 File The primer pairs location on respective transcript sequence**

Primer sequences were in red boxes. Primer pairs on two different blue sequences were on two different exon.

**>Glyma.02G091900.1**  ***Ac t*
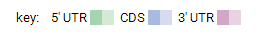
**

GCAAAAACCAAGCACCGTGGAAATCGCTGTTTGATTTAAAACCAAAAAAATCAACGGTCCACATTGCATCTCGTGGTTCTTATATTGAATTCCAATTTCCATTTCCACTTGGTCCTTCTCTCTCTCTCCCTCTTTCTCGTCACTAGCTCTGTGCATTCTTCTCTCGCTCTCTGCCTTCCATCACACTCGGCATTTCCTTTCCCAAGATAGTAGAAGATGGCAGACACTGAGGATATTCAACCTCTTGTTTGTGACAATGGAACAGGAATGGTTAAGGCTGGATTTGCTGGAGATGATGCCCCACGTGCTGTGTTTCCCAGCATTGTAGGTCGTCCTCGTCACACTGGTGTGATGGTTGGCATGGGCCAAAAAGATGCATATGTTGGTGATGAGGCACAATCTAAGAGGGGTATTTTGACTCTCAAGTACCCCATTGAACATGGAATTGTGAGCAATTGGGATGATATGGAAAAGATTTGGCATCATACCTTCTATAATGAGCTTCGTGTTGCCCCAGAAGAGCATCCAGTTCTCTTAACTGAGGCCCCTCTTAACCCCAAGGCTAATCGTGAGAAGATGACCCAAATCATGTTTGAGACCTTCAACACCCCTGCTATGTACGTAGCCATCCAAGCTGTTCTTTCACTGTATGCAAGTGGTCGTACAACTGGTATTGTGTTGGATTCTGGTGATGGTGTCAGCCACACTGTCCCTATCTATGAGGGTTATGCACTCCCACATGCCATCCTTCGTCTTGATCTGGCCGGTCGTGACCTCACTGATTTCTTGATGAAAATTTTGACTGAGCGTGGTTATTCCTTCACCACCTCAGCCGAGCGGGAAATTGTAAGGGATGTGAAAGAAAAGCTGTCTTACATTGCCCTTGACTACGAGCAAGAACTGGAGACAGCCAGGACCAGCTCATCCGTCGAGAAGAGCTACGAGTTGCCTGATGGGCAGGTCATCACTATTGGCGATGAGCGTTTCAGATGTCCAGAGGTTCTGTTCCAACCATCCATGATCGGAATGGAAGCTTCAGGCATTCATGAGACAACATACAACTCCATAATGAAGTGCGATGTTGATATCAGGAAGGACCTTTATGGTAACATTGTTCTTTCAGGAGGAACAACCATGTTCCCTGGTATTGCTGACAGAATGAGCAAGGAAATTTCTGCATTGGCACCCAGCAGCATGAAGATCAAGGTTGTAGCACCACCTGAGAGAAAATACAGTGTCTGGATCGGTGGTTCTATCTTGGCATCTCTTAGCACCTTCCAGCAGATGTGGATTGCAAAGGCAGAATATGACGAATCTGGTCCATCAATTGTGCACAGAAAGTGCTTCTAAACTGTTTCGATAGATAGGGTTATTGAAGGGAAAGACTACTATCATCACGATTTGTCTGGAAGCAATGCATTGCAGATTGTAACCCTTTGATTCTAGCTTTTGTTATCCTTTCATTTTATTCTCTTGTTGGAGTGATCATTTATTTGCAGAAGGTGAGGAGAGTTGTTTTTTAAAATGGTCCAGTTCTTTTCTTATACTTTGATAAGTTTTTAACTGGTTCATTCTGAGTATGAGTTAGCTGGATTATATCAAACTACGCTTGTACCCTTGGTATGCATAATTATAATCCTTCATCCGTTGAACTACAAGTTAACTCTTTTCAAATACAACAGTAGCAAGTTAATTCACTGAGC

**>Glyma.12G024700.1**  ***Cyp***

TTTACTTCACTCACACCCCGCCACGTGTTCCAATCGAACGGTCACTTCTGCATCACGCGCCACTATAAATATCTCTCTCTCGTCATCCGCAACCCCAAGCAAAACCCTAATCCCTCTTTCTTCCTCTTCCTCAGTAGTGCGATTTTCGATTCTCTTCTCTGCAACTATGCCTAACCCTAAGGTCTTCTTCGACATGACCATCGGCGGTCAACCTGCCGGCCGCATCGTGATGGAGCTCTACGCCGACGTGACTCCGAGCACCGCCGAGAACTTCCGCGCGCTCTGCACCGGCGAGAAGGGCGCCGGGCGGAGCGGCAAGCCCCTCCACTACAAAGGCTCGTCCTTCCACCGCGTGATCCCGAACTTCATGTGCCAGGGCGGCGACTTCACCGCCGGAAACGGCACCGGAGGCGAGTCGATCTACGGCGCCAAGTTCGCCGACGAGAACTTCGTGAAGAAGCACACTGGTCCCGGCATCCTCTCCATGGCGAACGCCGGTCCCGGAACCAACGGATCTCAGTTCTTCATCTGCACGACGAAGACGGAGTGGCTCGACGGAAAGCACGTCGTGTTCGGACAGGTCGTCGAGGGGATGGACGTCGTCAAGGAGATAGAGAAGGTCGGATCCAGCTCCGGCAGGACCGCCAAGCCTGTCGTCGTCGCCGACTGTGGTCAACTCTCTTAGATAGACGACGTTGACCCTAGGCTTTTATCTCTGTGTCGGTGGCTCTGAATGTGTCGTTTCTTTCGGTGTCGTTTTTGCCTTTTTTTTCTCTTTCCCATGTCGTTTAGGGGATTGCGGTGTTATGGATATCATTTCGAAACCCTTCCCCTTTGAATCCTTCCTTTCCTTTCTAAAATATCAGTATCATGTGGTTCGTTCATAGAGTGAATGGAGTCTGTTATGAGAAACATAAAAAAATATGATGAGCTACCTTCAATAAAAATTAGGACCTCTATTATTCGCCTAGTAATTCTCTTTAATCTTCGATTATTTTTTTTATATACGTTTAGTTGTTTCTGATTATAAATTGTGGAAAATTGGCGTTTATTAAATACCATAGTTTACGATAATTAGAACCTACAACTAGGAACGTTCATTGGTTTGATGGATTGATATTTGTGCAGATAATACATACGACTGTGTTATTTTTGATGAAATTGTGATCCGTGTTTCCCATCATTATTGTACTTTTATTGTCTTATCTTATAATATTATCTGTG

**>Glyma.19G052400.1**  ***EF1a***

ATCACTCTGCTGCCTCGCTCTGTTTCTGTCTCTGTGTTTGCGGCTGAGGATTCCGAACGAGCGACCTTCTTCGTTTCTCGCAAAGGTAACAGCCTCTGCTCTTGTCTCTTCCATTCGATCCATGCCTGTCTCTTCTTTACGATGATGTTTCTTCGGTGTATGTTTATTTATTTATTTATTTATGCTTTATGTTGTGAATGTTCGGTTGTTTTGTTTCGCTTTGCTTTTGTGGATTCTATTGGTTTTTGAATCAGTTAATCGGAAGAGATTTTCGAGTTGTTTTGTGTTTTGGAGGTGAATCTTTTTGTTGAGGTCGCAGATCTGTTGATTTTGTGTCATAAACGTGCGACTCTGTTTGATTTTTTACGAGGTTATGACGTTTTGGTTGTTTTATTATGGATCTGTTAAGGCAGAACCATGATTTATGTTTATGTTCGTTTACACGATTAAATTTTCTTGTAACACGATGAAGTTTTTTTAAACACGTTGAAGGAGTCTTGTTGATATGAATTTGTCGATTGTTTTTTTGTGGTTTTGTTCTCACGTTATCAAGCGTAATCTTTTACTATGTACGCGAACATATCTAGATCTAGCAGAGCTTTTTTTTTTTTTAATTCTTTGTGAAGCTTTTGAAATATGAAATTTGTTTTTCAAATTTTTTTTTAATTTATTGAAAACACTGTGGATACTGAATAAGGTTCTATGTATGATGCGAACCATGTTTGATATGTTTGTTTTTGTTGTTCCATATGGATTTTTCTGTTAGATTTCCATATGCTTTTGAGTTTGTTTTTGCTGTTACAGATTGATTTACTTTTTAGAATATTTCTCTTGGCTTTCACTGTTTTAGAAATTTTTTGTTATTGGTAACTATAAATGTGTGAATTTGGATTATACCTTTACCTTGTTATTTAGTTGTGTGATAATTTGGTTTATAGTTATTTTGAGTTCTGACTCGTGTTTCTTTGAATTGATTCCAGTTTAAGTCATCATGGGTAAGGAAAAGACTCACATCAACATTGTCGTCATTGGACATGTCGACTCTGGGAAGTCAACTACCACTGGTCATTTGATCTACAAGCTTGGAGGTATTGACAAGCGTGTGATTGAGAGGTTCGAGAAGGAGGCTGCTGAGATGAACAAGAGGTCATTCAAGTATGCCTGGGTGCTTGACAAGCTCAAGGCTGAGCGCGAAAGAGGAATTACCATTGATATTGCTTTGTGGAAGTTTGAAACCACCAAGTACTACTGCACAGTCATCGATGCCCCCGGACATCGTGACTTTATCAAGAACATGATTACTGGTACCTCCCAGGCTGATTGTGCTGTCCTTATCATTGACTCCACCACTGGTGGTTTTGAAGCTGGTATTTCTAAGGATGGACAGACTCGTGAGCATGCTCTTCTTGCTTTCACCCTTGGTGTGAAGCAGATGATCTGCTGCTGTAACAAGATGGATGCCACTACCCCGAAGTACTCTAAGGCTAGGTATGATGAAATTGTGAAGGAAGTCTCTTCCTACTTGAAGAAGGTTGGTTACAACCCAGACAAGATTCCATTTGTGCCCATCTCTGGTTTCGAGGGTGACAACATGATTGAGAGGTCCACCAACCTTGACTGGTACAAGGGACCAACTCTCCTTGAGGCTCTTGACCAAATCAATGAGCCCAAGAGGCCCTCAGACAAGCCACTCAGGCTTCCATTGCAGGATGTCTACAAAATTGGTGGTATTGGTACCGTGCCAGTGGGACGTGTTGAGACTGGTGTCGTGAAGCCCGGTATGGTGGTGACTTTTGGTCCCACTGGGCTGACAACTGAGGTTAAGTCTGTTGAGATGCACCATGAGGCTCTCACAGAGGCTCTTCCAGGTGACAATGTTGGCTTTAATGTGAAGAATGTTGCAGTCAAGGATCTCAAGCGTGGTTTTGTTGCATCCAACTCCAAGGATGACCCTGCCAAGGAAGCTGCCAACTTCACATCTCAAGTCATTATCATGAACCACCCTGGCCAGATTGGAAATGGATACGCACCGGTGCTTGATTGCCACACTTCTCACATTGCTGTCAAGTTTTCTGAAATCTTGACCAAGATTGACAGGCGATCTGGTAAGGAGCTTGAGAAGGAGCCCAAATTTTTGAAGAATGGTGATGCAGGTATGGTTAAGATGATTCCAACCAAGCCCATGGTGGTTGAAACTTTCTCTGAGTATCCTCCCCTTGGTCGTTTTGCTGTGAGGGACATGCGTCAAACTGTGGCTGTCGGAGTCATCAAGAGTGTTGAGAAGAAAGACCCCACCGGAGCCAAGGTCACCAAGGCCGCTGCCAAGAAGAAGTGATTGCATTTGGGCAATTTTGCTAGCACATGTGATCATCATCGTGGTTACTCCTTTATAGTAGTTTTATCCTTGCAGAGTCTTAGGTGTTTTGTTTAAGTTATATTTTTAAGTTTCTGCCGATTTCATGTAGCCGTAACTTTCAAAACTAGGTTCTTGATCGGCGGTGGTCAATTTTCATTGCTGTTTGTTTTTGATGAGTACTGTTTTTTGTTTTGATGGTAAGAAGAGTCTGAGATATTTCGAATTTCACAAGCAGCTATAGGGTTTTAGTCCATTTCCTTTGCTGCTGAGGGATGTTTTAAGTTGCATTTAATTTATAACGAAGTTTTATAAACTGTTTATGGTTTAAAGGCTATTATTCTTTAA

**>Glyma.14G039100.1 *EF1b***

GTGAATTTGAGTTCAGAATACAGAAGTGGGGACTGATCTAGGGTTTGTGACTGTACATGCTATAAATATAAATTATTGAGCATCGAAGGAGTGTCTCTCTTTTTCACTCACTCTGCACCCACCATTACTCAGCACTGCCACTCTTCAATCACACTAATGGCTGTCACCTTCTCAGATCCTCACACCGAAGAGGGCATCAAATCCCTCGATCAATTCCTTTCTGGGAAAACCTATATCTCTGGGGATCAATTGACAAAGGATGATATCAAAGTGTATGCAGCTGTTGTGGAAAAGCCAGGAGACTCTTTTCCTAATGCTGCTAAGTGGTACGATGCCGTCTCTTCTCAACTTGCTCCAAGCTTCCCTGGGCACGCTCAAGGGGTAAGATTCAGTGGCGCTGCTGCTCCAGCTGAAGCTGCACCTGCCAAAGCGGCTGCCACTGCTGAAGAAGATGATGATGATCTTGATCTCTTTGGTGATGAGACAGAGGAGGATAAGAAGGCAGCAGAGGAAAGGGAGGCTGCTAAAAAGCCTGCCAAGAAAAAAGAGAGTGGCAAGTCTTCTGTCCTTCTCGATGTTAAGCCTTGGGACGATGAGACCGACATGAAGAAGCTGGAAGAGGCTGTCCGTAGTATTGAGATGCCTGGTTTATTGTGGGGAGCATCCAAACTAGTTCCCGTGGGATATGGTATCAAGAAGTTGCAGATCATGATGACTATTGTTGATGACCTTGTATCAGTGGACACTCTTGTTGAGGAACGCTTGACAGTTGAGCCATGCAACGAGTATATCCAGAGCTGTGACATTGTTGCATTCAACAAAATCTAAGTTTTCTTGTTGACTTTTGAGTTTGGCCATTTTCAAGCGGCTGATCAGTCTGTTACTCTTCCTTTAATCGTTCATGTTTGGTCTGTTTCAATGGTGGTTTTCTTGCAAGAATTGTGAGAAGTAAAAAGAGATATTATAGATATATCAAGTATCCATGTTATGGTGGTTTTCTTGAATTAGATATATGGGATTAAAATAACTGAACGATGTGCTTCCATTTAAATTTTGATAAGTTTCTCTAAACCTCTAAATTTCATTGAATGAGCTTCAGGCTATATAGAATAAAACCAGTTTGGTTTTTGACTTATTATGAT

**>Glyma.12G051100.1 *Fbox***

GTTTTTGTTGTTTGGTGTTATTTTATTTTTTTAATCCGTAAAAATAAAAAGAAATACAACATTTCACCCACCCTTGTTGTTATGTGAGTGCGGCAATAGTGGAATAGCGGTAGAGTGTGCGATCGAGAAAGCAGAAAGCAGAAAGATGGGGTTGGAATCGGTGGGAGATTTAGCGATTAACGTGATTCTGAAAAAATTAGGAGCCCAAGACATTGCGAGAGTGGCGTGTGTGAGCAAAAGGTTCAGTTCTTCCGCTTCCGATGACACTCTTTGGATCAATCTCTGCTTCAATGAACTCGCTTTGACACAACCCCTCGATCATCTCGGAAACCCTCTCTCTTCCTTCAAGGAATGCTATCTAGCATGGAGAGGAGCTTTTGTTATGTACCCTTGGTCTCTTGTTAAGCGTGTAAAAAGGTGCTGGGATAGAATAAAAACCTGGTTGACCAATAATTTTCCTGAAGCGGAGGCCACTCTTTGTAAAGGTGCAACTGAAGCTGACATTCAGGAGTTGGAGAATGTATTAAAGGTGAAATTGCCTCTTCCTACAAGGATCCTTTATCGCTTTCACAATGGGCAAGAATTTGCAAAGGCAGATCCAGAAACTAGTACATTTGGCAGATCTTTGGGTCTAATTGGTGGCTACTCCTTCTATGGTCATTTGGTGAATGTTTATCTATTACCTATATGTCAGATAATCCTAGAAACTCAGCAAACTAGGCGTCGCTTGAGCTTTTTAAGAAGATCAAAGTATGTTCTTGTGGCTGCTTCATCCACATACAGTAGAAAGTTGTTTTTCCTCAACTGTACCAATGGTCAACTATATGTCGGGACCAGGTCTCCTCTTACCGAAAGAGACATAATCCCTTGTGTACCTCATGACCTGATTAGTTTACATCAGGAATTGAATAGTTCAGAGCAACAAGATGCCATGCTACTGTGGTTAGAAGAACATGGTCGCCGTTTAGAACACGGCTTTATCAAACTTCATGATGAAGGAAATGGCAAAAGCATTAATCTTTTCCCAGAAGAACCCCATATTTGTTCAACGGCTGTTACTAATGGTGTGAAGGTTCGCGCTTCTGCACTGGTTATCCCTGAGTTGATGGATCTTCAAGATGACCTTGGAGAGTACTTATTTGCTTATTCAATCCGCTTGTCCCTTGAACCTCAAGGATGCATTATTAATGGAATGTCCTTCAGCTCTTGCCAGCTCCATTGGAGGCACTGGATCATCCGTGCTAATGATATTGTTATATCTGATGTCAGTGGAAAAGCTGTTATAGGACAGTTTCCACTTTTGCGTCCGGGTGCTCAAGAATTTGTTTATCAGAGTTGCACGCCTCTACCAACACCATCAGGTTCTATTGAAGGTTCTTTTACATTTATACCCGGCAGATTGGCAGACCCAAAAGGAGACCCTTTTCTAGCTACAGTGGCTCGTTTCCCGCTCCAGCTGCCAGACTATATATTCTGATTTTGATTCTGGATGGGATTGAAGTATCTCTAATGGCAATTGCAGCTCTCAGATATTGTTTGGGTGTATTATGTCCCTTTCGGGAGAGGTTTTTAAGTTGGACCTGCACAATTTCCCTATCTATAAGAGAAGCAAAGTATGTACAATATTTGATCTCTATCATAAGAGAAGCAAAGTAGATACAATATTTGTTAGGGGTGTTCATGGTTTGATTAGGTTGGATTTTTTGTTAAAAAATCATCCAAATCAAACTTAAAAAACTTGTGATTTGGTTCGATTCAGTTTTCATTTAAAATAAAATTCAAATCAGACCAAGTTAATATATATTGCACTTTAGTTCAA

**>Glyma.19G127700.1 *TuB4***

CAGCGCCTTCTGAGGACAAAGCGAGGGAACAAACGTGAACCGTGGAGTCTCACTATAAAAATCTCGCTCACTAACTCTTGTTCTTCCAAAACCAATTCTCTGCACTCTTCATCAAGCTCCGCTCCAACACAGGGGAAAATGAGGGAGATCCTTCACGTGCAGGGAGGGCAATGCGGGAACCAGATCGGTTCCAAGTTCTGGGAAGTGGTGTGCGACGAGCACGGCATAGATCCGACGGGGAAGTACGTCGGAAACTCAGATCTGCAACTCGAGCGCGTGAACGTCTACTACAATGAAGCCTCGTGCGGGCGCTTCGTGCCACGCGCGGTGCTGATGGACCTGGAGCCCGGAACCATGGACAGCGTGCGGACCGGGCCGTACGGGCAGATCTTCCGCCCCGACAACTTCGTGTTCGGGCAGTCCGGCGCCGGCAACAACTGGGCTAAGGGGCACTACACCGAGGGCGCCGAGCTCATCGACTCCGTCCTCGACGTCGTGCGTAAGGAGGCCGAGAACTGCGACTGCCTCCAGGGGTTCCAGGTCTGCCACTCGCTCGGCGGCGGAACGGGCTCCGGGATGGGGACGCTTCTTATTTCCAAGATCAGAGAGGAGTATCCTGACAGGATGATGCTTACTTTCTCCGTTTTTCCTTCGCCCAAGGTCTCCGACACTGTTGTTGAGCCTTATAACGCTACTCTCTCTGTTCACCAGTTGGTGGAGAATGCTGATGAGTGTATGGTGCTGGATAATGAGGCGCTCTACGATATCTGCTTCAGGACTCTCAAGTTGACCACTCCTAGCTTTGGTGACTTGAATCACTTGATCTCCGCAACCATGAGTGGTGTTACATGCTGTCTTCGTTTCCCTGGTCAACTCAACTCTGATCTGAGGAAACTGGCCGTGAATCTCATCCCTTTCCCTCGTCTGCACTTCTTCATGGTTGGATTTGCGCCTCTCACCTCTCGTGGCTCTCAGCAGTACCGTGCATTGACAGTTCCAGAGCTGACACAGCAAATGTGGGATGCCAAGAATATGATGTGTGCCGCTGATCCCAGGCACGGGCGTTACCTCACGGCATCAGCCATGTTCCGTGGCAAGATGAGCACGAAGGAGGTGGATGAGCAGATGATAAACGTGCAGAACAAAAACTCTTCGTACTTTGTCGAGTGGATTCCCAACAATGTCAAGTCGAGTGTGTGTGACATTGCTCCTAGAGGGCTCTCCATGGCGTCCACATTCATTGGAAACTCGACCTCGATTCAGGAGATGTTCAGGAGGGTGAGCGAGCAGTTCACGGCCATGTTTAGGAGGAAGGCTTTCTTGCATTGGTACACTGGTGAAGGCATGGACGAGATGGAGTTCACGGAGGCAGAGAGCAACATGAATGACCTTGTGTCTGAGTACCAGCAGTACCAGGACGCCACTGCTGAGGACGAAGGGGAGTACGAGGATGAGGAGGAAGAGGATGGTGAAGCAGACGACCATATGTGAAGAGACTAAAATGTGAAGATGCCTGTGTCTTACTTATGCTATGTGATGCGTAAGTGTGTTTGCTCTGCACTCCAACGTGAAGAAGATTGTAATTACTAATAGTGTATGTACGGTTTGCGGGATAATACTTTGATGGTAGTGAGAGTGGAGTTTGGGACCTTTGGGTACATGTTATGCGGCACCTATCGGTTTGTTCTTATATTGGCTTGCTTTTTATTTTTATTTTTGTCCATCTGGTATATTGGATCTCATGTTTCCGTAATATGTTGTTGTTCTTATGCATGGCTTTTGTTTACATTGAATTCTTGGAATGTCTTGGGGGAAAGTCGTGCCTCATGTTTATGGCTCAAGAACCTTGCGCTGCCCATGTTATCCTTTGAGAAAGTGGAGGATTAATTAATCCCTTGCTTTACTTATATGCATAACAGAGCAACAAAGATTCCAATGCAATTAGAATGCAGAAGGAAACTGCTACCCGGTGAGAATTAAGTTTTATCACGCATCTAACCTTGTCCAAATTGCAGGACAGATCAATATGCCCTCAATTTGTAGGGGTAGTAGAGTTTTTGGAGTGAAAATCCGTTTCAAATTGTGGTCGTCCAGGGTGGTGGGTCTTCGGGTTTTATAGTAATTTTGATTCAATTTCACGC

**>Glyma.05G157300.1  *TuA5***

AGGAGTATGTTAAGACTTGAGAGTGAGAGAGAGAGTGAGAGAGTGAGTGAGATAGAGAGAGACGCTTCGCAAATCTCAATCGAATCTCAGAAAAATGAGAGAAATCATCAGCATTCACATAGGTCAGGCCGGGATCCAGGTCGGAAACTCCTGCTGGGAACTCTACTGCCTCGAACATGGCATCCAGCCCGACGGCATGATGCCTTCTGACTCCACCTTCGGTGTAGCCCACGACGCCTTCAACACCTTCTTCAGCGAAACCGGATCTGGCAAGCACGTCCCCCGTGCTGTCTTCGTCGACCTCGAACCCACCGTCATCGACGAGGTCCGCTGCGGCACCTACCGTCAACTCTTCCACCCCGAACAACTCATCTCCGGCAAGGAAGACGCCGCCAACAACTTCGCCCGCGGCCACTACACCGTTGGCAAAGAGATCGTAGATCTGTGCTTGGATCGCGTCCGCAAGCTCGCCGACAACTGCACCGGCCTACAAGGCTTCCTCGTCTTCAACGCCGTCGGCGGTGGCACCGGTTCCGGTCTCGGATCTCTCTTACTCGAGCGTCTCTCCGTCGATTACGGCAAAAAATCCAAATTAGGGTTCACCATTTACCCTTCCCCACAGGTTTCAACCGCAGTCGTTGAACCCTACAACAGCGTCCTCTCCACCCACTCCCTCCTGGAACACACCGACGTGGCGGTCCTCTTGGACAACGAAGCCATCTACGACATCTGCAGGAGATCCCTCGACATCGAGAGACCAACCTACACCAACCTCAACAGGCTCATTTCCCAGATCATTTCGTCCCTCACCACTTCCTTGAGGTTCGATGGTGCCATCAATGTGGATATCACTGAGTTCCAGACCAACCTTGTGCCCTACCCTAGGATCCACTTCATGCTTTCGTCCTATGCTCCGGTTATCTCTGCCGCCAAGGCCTACCACGAGCAGTTGTCGGTGCCGGAGATCACCAATGCCGTGTTCGAGCCCGCCAGCATGATGGCCAAGTGTGATCCAAGGCACGGCAAGTACATGGCTTGCTGCTTGATGTACCGTGGTGATGTTGTCCCTAAGGATGTCAATGCTGCTGTTGCCACCATCAAGACTAAGAGGACTGTTCAGTTTGTTGATTGGTGTCCAACTGGATTCAAGTGTGGTATCAACTACCAGCCACCTTCTGTTGTTCCTGGTGGTGACCTTGCTAAGGTTCAGCGTGCTGTATGCATGATCAGCAACAACACCGCAGTTGCTGAGGTGTTCTCTCGCATTGACCACAAGTTTGATCTGATGTATGCCAAGAGGGCTTTTGTTCACTGGTATGTTGGTGAGGGTATGGAAGAAGGGGAGTTTTCTGAGGCTCGTGAGGACCTTGCTGCTCTTGAGAAGGACTATGAGGAGGTTGGAGCTGAAGGAGCGGAAGATGATGAGGAAGGAGAGGACTATTGATAATCGAAGGCTTTTTTTTGTTATCAATGTAATTGTTTCCGAGTTAAAAGGGTATATGCTTCGTTACATTACCCTGAACCATTATTGTTCGTTCTTTTTTGTTTTTGCTCCTGCTGTGTGATATAGTTCTCCATTATTCAAACTGTGAAGTGATCAACATTATGCTTCTATTTGGTGGTTTAGGACCTGTTGTTTGCGATCCTCAGTCATGTATTCAGAATTGTCCATGTCAAAAGTTGATTTCTTATGTTTATCTCGCAAATTTTACCGGATTTCCCGTTTTGATTATCTCTTTATGAACCGTGAAAAACGTACCCCTCGTATGTTTTTTGCAATTCGCTTCATTCTGG

**>Glyma.13G318800.1 *60s***

CAAAATAAAGGGTTTTAGAATTAGGGCTTTTTTCAGTTTTCCTCTTCTGAATCCACCGCCACCGCAGCGTGGACGGAATCAGAGTGCAGCCATAGCAGCAGCAGCAGCAGTTATTGAAGTGCTGCTCTAGTTTGAGGCACAATGAGATTGGAGAAATGCTGGTTTTGCTCTTCAACCGTATACCCCGGACATGGAATCCAGTTTGTTCGTAATGATGCAAAGATTTTTCGGTTTTGTAGATCAAAATGCCACAAGAACTTTAAAATGAAGAGAAATCCTCGTAAGGTAAAGTGGACCAAGGCATATCGTCGAGTGCATGGAAAGGATATGACTCAGGACTCAACCTTTGAGTTTGAGAGAAAACGAAACAGGCCTGAAAGATATGACAGGAATCTTGCGGAGAATGTCCTGAAGGCCATTCCTAAGATTGATAAAATCAGAGTCACCAGGGAGGAGAGACACCATAAGAACAGGATGAAAGGAAAAAAGGAAAAGCTGCTGAAGGAGGCAGTGAAGGAGTTGGAGCAGGGCATCAGTTTGGTCAAAGCTCCTTCTGTGCTTCAACAGGATCCATCTCTCACATTACCAAAGATCAAAGTCAAGGTTTCCCAACAGCAATCAGAGGAGAATCATGCCATGGAAGAGTAATTCTAACAATGCTGCACTTAATTTTTGCCGTTTTAATATTTGTTGCTAAATTAGTTGCCTGTAAGGTCCAGTTTACCAGTTTCCTTGTGATCATTAATGTGAATGATGTGTTCTTCAAACATTCGATGTATTTGTCTCTTAAAACTATGGTTCTCTGAGATAGTCAACTATTATAAAGGAAATCGGAAAGTTGTTGCTATAGCCT

**>Glyma.01G144900.1 *GRF9*** TTGCAACGTAATAAGCTGTTTGTCAGAAACTTCTGACACCACTCTCTCTCTCTCTCTCTCTCTCTCCACATTTTGCATCTTTCAATAGAAGTAAATGGAGGCAGCAGCAGAAGCACTTCCGTGCCGAGCTTACCCTTTCTGAATCCCCTTTTAACAGTTTTTTATTATTCACACGGAAAAAAAGTAAGAAAAGAAGCTTTTTTCCCCCTTGATCATCACTATCAGATACAGACCCTCTAGAGAAAAAGGAAAAAAAAGCCTTCATGTGAATGTGACAATGGAAGCTAAGCCTCTTCGAACTGTTCCCTCTTCACACAACACTTCTGGAGGTGGACCCCAGAAGAAGATTGACATGGGACACAAGATGGTAGGTGATGTTGGTGTTGTTGTTGTTGATGAAAGAAAGAGGGTTGTTGTGGTCAAGGAAGAGAAAGAGAACACTTCTTCCTACAGTGTTGAACTTCATCTCGGGGTTGATGCTTCTCATTCTGCTCCACAAGAAATAAACCATGTGATCACTGAAGCTCAGAGGCGTGAGCTTCACCATCAAGTTTTCATCTTCAACCATTTGGCATATAATCTTCCTCCTCCTTATCACCTTGTGCAATTCCCGAGCAACATGTCAGAATACAGTTTCCTGGGTTTTGATCATGGGAGTATGGTGGATCCAGAGCCTCATAGGTGTAGAAGAACTGATGGAAAGAAATGGAGGTGTGGTAAGAATGTGGTGCCTAACCAGAAGTACTGTGAAAGACACATGCATAGAGGTCGAAATCGTTCAAGAAAGCCTGTGGAAACATCTCAAATTAACTCTCATCTGGCAACAAAGCCATCTAGCAAGTCACACAACAAACCCGCCTCAAGGACACAATTTGAAATTTCAAATCCAAACCTTATGGCCATTCGACATAGTGACACATCAAGTACCCCATCAAGGAGTCTCAGTGTTGCCAATTGCTCTTCTGCTAATAATAGGTGGAAAAATAGTGCAAGTTATGCTGACTACCTCACATCGTTTTCTTCTGCGTCTGCAGTGTCCCCTGGATCCACTCTTGCCACTCCAGTTGCTCCTAAGATGGCCACCTTCAGCAGTGTGACATCCATTGCTTCAGATAGAGGAAGTTGCCTAAATATATGCCAGAAAGATAACAAGTCCAAGAGCTGTATCAGCAACAACATCAGTGTTAAAAGTGGTGGGAAAGGAAGAATTGTTGGTGACACTAATGGCATTTCTACTGGAATAGGCTTCTCCCCAACGAGTGTTCTTCAAGTTTCTGGTTGCAACCCTTCATACCTGAATGACAGAACCAACATAGAATCTGCATCCGGTAGGTGCCGGAGAACAGATGGTAAGAAGTGGCAATGCAAGAGTGCTGTTCTTCCGGGTCAGAAGTACTGTGCCACACACATGCACAGAGGTGCTAAAAAGCGGCTCACAAGCCATGAACCGGCAGTTACCATTGCGCGGTTGCCTAACTCTTCAGTCACAACCAAAATGCAGAAAGCGCATTGTGCAATTCCAAACACAAATCTTTCCATGTCAGTCCCAGCAAGTGAACCATTCATACAATGTAATGAGAAAAGTCAAAGTACCAGTGACACTGATACCACCATCAGTGACACCTTGAATGAGTGTAGCTATGCTTCTTTCTGAGAAGCACTTATGTGTTTTGTTTTAACTTGTAACATTTGAGGTCTCAGTTATGTGTGGACTGAACAATGATCTCCATGAAGAAAAGATTAACCATGTGTTTGTTTTTAATCATGTCATCCTCACAATGTGAAGTAAAGTGGAAAAAAATATAACAAATTGGGGTCACCCGGACTTGAACCAGAGACCTTCAGTGTGTTAGACTGATATGATAACCAACTACACCACGCCACCACATGGTGTTGCTGCTACATTTCTTTTTGCAGTTTTGAATAGA

**>Glyma.13G159700.1 *Pre-miR396a*** ATGGCTCTCTTTGTATTCTTCCACAGCTTTCTTGAACTGCATCCAAAGAGTTCCTTTGCATGCATGCCATGGCACTCTTACTCCCAAATCTTGTTTTGCGGTTCAATAAAGCTGTGGGAAGATACAGATAGGGTCAACAACGGACTAGCTTATCTCAAACTCCAGATTCTTCTCTAA
