# Supplementary material for: Normalization for Relative Quantification of mRNA and microRNA in Soybean Exposed to Various Abiotic Stresses
Source: PLoS One. 2016 May 13;11(5):e0155606. doi: 10.1371/journal.pone.0155606 (PMC4866712; doi:10.1371/journal.pone.0155606)
Supplement: S3 File — (DOC) [file pone.0155606.s004.doc]

**S3 File NormFinder Analysis**

**No.1** NormFinder analysis of **candidate reference mRNA genes** for mRNA and miRNA precursors normalization in **leaf** tissue samples

| **Gene name** | **Stability value** |  |  |  |  |
| --- | --- | --- | --- | --- | --- |
| *Act* | 0.099 |  | **Best gene** | | *TuB* |
| *Cyp* | 0.198 |  | **Stability value** | | 0.093 |
| *EF1a* | 0.114 |  |  | |  |
| *EF1b* | 0.109 |  | **Best combination of two genes** | | *Act and EF1b* |
| *Fbox* | 0.160 |  | **Stability value for best combination of two genes** | | 0.063 |
| *TuB* | 0.093 |  |  | |  |
| *TuA* | 0.164 |  |  | |  |
| *60s* | 0.117 |  |  |

| **Intragroup variation** | | | |  |  |  | **Intergroup variation** | | | |  |  |
| --- | --- | --- | --- | --- | --- | --- | --- | --- | --- | --- | --- | --- |
| Group identifier | 1 | 2 | 3 | 4 | 5 |  | Group identifier | 1 | 2 | 3 | 4 | 5 |
| *Act* | 0.001 | 0.002 | 0.002 | 0.000 | 0.001 |  | *Act* | -0.098 | -0.095 | 0.059 | 0.030 | 0.105 |
| *Cyp* | 0.102 | 0.042 | 0.085 | 0.060 | 0.082 |  | *Cyp* | 0.075 | 0.276 | 0.024 | -0.125 | -0.251 |
| *EF1a* | 0.027 | 0.006 | 0.005 | 0.008 | 0.009 |  | *EF1a* | -0.055 | -0.061 | -0.051 | 0.052 | 0.115 |
| *EF1b* | 0.001 | 0.010 | 0.011 | 0.000 | 0.062 |  | *EF1b* | 0.091 | 0.024 | -0.060 | -0.059 | 0.004 |
| *Fbox* | 0.099 | 0.020 | 0.044 | 0.013 | 0.004 |  | *Fbox* | 0.100 | -0.054 | 0.008 | 0.126 | -0.180 |
| *TuB* | 0.010 | 0.012 | 0.018 | 0.026 | 0.010 |  | *TuB* | -0.017 | -0.015 | 0.028 | 0.012 | -0.007 |
| *TuA* | 0.157 | 0.037 | 0.022 | 0.027 | 0.019 |  | *TuA* | -0.171 | -0.091 | 0.007 | 0.013 | 0.242 |
| *60s* | 0.021 | 0.021 | 0.003 | 0.043 | 0.043 |  | *60s* | 0.075 | 0.016 | -0.016 | -0.049 | -0.027 |

**No.2** NormFinder analysis of **candidate reference mRNA genes** for mRNA and miRNA precursors normalization in **root** tissue samples

| **Gene name** | **Stability value** |  |  |  |  |
| --- | --- | --- | --- | --- | --- |
| *Act* | 0.146 |  | **Best gene** | | *EF1b* |
| *Cyp* | 0.191 |  | **Stability value** | | 0.106 |
| *EF1a* | 0.145 |  |  | |  |
| *EF1b* | 0.106 |  | **Best combination of two genes** | | *EF1b and 60s* |
| *Fbox* | 0.149 |  | **Stability value for best combination of two genes** | | 0.081 |
| *TuB* | 0.240 |  |  | |  |
| *TuA* | 0.157 |  |  | |  |
| *60s* | 0.141 |  |  |

| **Intragroup variation** | | | |  |  |  | **Intergroup variation** | | | |  |  |
| --- | --- | --- | --- | --- | --- | --- | --- | --- | --- | --- | --- | --- |
| Group identifier | 1 | 2 | 3 | 4 | 5 |  | Group identifier | 1 | 2 | 3 | 4 | 5 |
| *Act* | 0.003 | 0.013 | 0.019 | 0.009 | 0.023 |  | *Act* | -0.029 | -0.096 | -0.105 | 0.198 | 0.032 |
| *Cyp* | 0.060 | 0.064 | 0.088 | 0.019 | 0.007 |  | *Cyp* | 0.082 | 0.074 | 0.130 | -0.119 | -0.166 |
| *EF1a* | 0.023 | 0.001 | 0.016 | 0.018 | 0.001 |  | *EF1a* | 0.145 | -0.048 | 0.116 | -0.085 | -0.128 |
| *EF1b* | 0.016 | 0.004 | 0.007 | 0.022 | 0.000 |  | *EF1b* | 0.082 | -0.089 | 0.060 | -0.035 | -0.018 |
| *Fbox* | 0.034 | 0.010 | 0.039 | 0.002 | 0.035 |  | *Fbox* | -0.121 | 0.074 | 0.157 | -0.028 | -0.083 |
| *TuB* | 0.229 | 0.004 | 0.146 | 0.070 | 0.014 |  | *TuB* | -0.207 | 0.198 | -0.283 | 0.151 | 0.141 |
| *TuA* | 0.068 | 0.063 | 0.004 | 0.029 | 0.018 |  | *TuA* | 0.113 | -0.163 | 0.021 | -0.064 | 0.093 |
| *60s* | 0.081 | 0.008 | 0.002 | 0.019 | 0.030 |  | *60s* | -0.066 | 0.050 | -0.095 | -0.018 | 0.130 |

**No.3** NormFinder analysis of **candidate reference miRNA** for mature miRNA normalization in **leaf** tissue samples

| **Gene name** | **Stability value** |  |  |  |  |
| --- | --- | --- | --- | --- | --- |
| *156a* | 0.080 |  | **Best gene** | | *166a* |
| *166a* | 0.021 |  | **Stability value** | | 0.021 |
| *167a* | 0.032 |  |  | |  |
| *171a* | 0.034 |  | **Best combination of two genes** | | *166a and 167a* |
| *172a* | 0.180 |  | **Stability value for best combination of two genes** | | 0.020 |
| *393a* | 0.209 |  |  | |  |
| *397a* | 0.218 |  |  | |  |
| *1520d* | 0.061 |  |  |

| **Intragroup variation** | | | |  |  |  | **Intergroup variation** | | | |  |  |
| --- | --- | --- | --- | --- | --- | --- | --- | --- | --- | --- | --- | --- |
| Group identifier | 1 | 2 | 3 | 4 | 5 |  | Group identifier | 1 | 2 | 3 | 4 | 5 |
| *Act* | 0.049 | 0.017 | 0.032 | 0.021 | 0.015 |  | *Act* | 0.065 | 0.077 | -0.110 | -0.042 | 0.010 |
| *Cyp* | 0.002 | 0.001 | 0.001 | 0.002 | 0.002 |  | *Cyp* | 0.051 | -0.008 | -0.009 | -0.004 | -0.030 |
| *EF1a* | 0.000 | 0.001 | 0.004 | 0.019 | 0.005 |  | *EF1a* | 0.060 | -0.014 | -0.024 | -0.084 | 0.061 |
| *EF1b* | 0.000 | 0.008 | 0.002 | 0.002 | 0.020 |  | *EF1b* | -0.014 | 0.147 | -0.075 | 0.027 | -0.085 |
| *Fbox* | 0.124 | 0.185 | 0.128 | 0.149 | 0.074 |  | *Fbox* | -0.237 | 0.051 | 0.048 | 0.151 | -0.013 |
| *TuB* | 0.222 | 0.150 | 0.157 | 0.189 | 0.162 |  | *TuB* | -0.053 | -0.094 | 0.057 | 0.091 | -0.001 |
| *TuA* | 0.178 | 0.474 | 0.067 | 0.163 | 0.168 |  | *TuA* | 0.081 | -0.341 | 0.210 | -0.075 | 0.124 |
| *60s* | 0.011 | 0.045 | 0.011 | 0.005 | 0.014 |  | *60s* | 0.047 | 0.180 | -0.096 | -0.065 | -0.067 |

**No.4** NormFinder analysis of **candidate reference miRNA** for mature miRNA normalization in **root** tissue samples

| **Gene name** | **Stability value** |  |  |  |  |
| --- | --- | --- | --- | --- | --- |
| *156a* | 0.109 |  | **Best gene** | | *167a* |
| *166a* | 0.102 |  | **Stability value** | | 0.067 |
| *167a* | 0.067 |  |  | |  |
| *171a* | 0.095 |  | **Best combination of two genes** | | *167a and 171a* |
| *172a* | 0.225 |  | **Stability value for best combination of two genes** | | 0.067 |
| *393a* | 0.143 |  |  | |  |
| *397a* | 0.142 |  |  | |  |
| *1520d* | 0.129 |  |  |

| **Intragroup variation** | | | |  |  |  | **Intergroup variation** | | | |  |  |
| --- | --- | --- | --- | --- | --- | --- | --- | --- | --- | --- | --- | --- |
| Group identifier | 1 | 2 | 3 | 4 | 5 |  | Group identifier | 1 | 2 | 3 | 4 | 5 |
| *Act* | 0.039 | 0.057 | 0.014 | 0.049 | 0.024 |  | *Act* | 0.008 | -0.118 | 0.119 | 0.006 | -0.014 |
| *Cyp* | 0.001 | 0.029 | 0.006 | 0.042 | 0.003 |  | *Cyp* | 0.188 | -0.136 | 0.018 | -0.076 | 0.007 |
| *EF1a* | 0.000 | 0.005 | 0.005 | 0.003 | 0.003 |  | *EF1a* | 0.046 | -0.090 | 0.054 | 0.030 | -0.040 |
| *EF1b* | 0.011 | 0.016 | 0.025 | 0.026 | 0.047 |  | *EF1b* | 0.050 | -0.057 | 0.024 | -0.063 | 0.046 |
| *Fbox* | 0.253 | 0.145 | 0.262 | 0.136 | 0.180 |  | *Fbox* | -0.043 | 0.107 | -0.260 | 0.026 | 0.170 |
| *TuB* | 0.056 | 0.100 | 0.048 | 0.004 | 0.099 |  | *TuB* | -0.006 | 0.126 | -0.096 | -0.154 | 0.130 |
| *TuA* | 0.076 | 0.008 | 0.115 | 0.005 | 0.088 |  | *TuA* | -0.089 | 0.069 | 0.022 | 0.181 | -0.182 |
| *60s* | 0.046 | 0.183 | 0.007 | 0.010 | 0.033 |  | *60s* | -0.154 | 0.100 | 0.120 | 0.051 | -0.117 |
