# Supplementary material for: Normalization for Relative Quantification of mRNA and microRNA in Soybean Exposed to Various Abiotic Stresses
Source: PLoS One. 2016 May 13;11(5):e0155606. doi: 10.1371/journal.pone.0155606 (PMC4866712; doi:10.1371/journal.pone.0155606)
Supplement: S4 File — (DOC) [file pone.0155606.s005.doc]

**S4 File BestKeeper Analysis**

**No.1** BestKeeper analysis of **candidate reference mRNA genes** for mRNA and miRNA precursors normalization in **leaf** tissue samples

| **CP data of housekeeping Genes** | | | | | | | | |
| --- | --- | --- | --- | --- | --- | --- | --- | --- |
|  | ***Act*** | ***Cyp*** | ***EF1a*** | ***EF1b*** | ***Fbox*** | ***TuB*** | ***TuA*** | ***60s*** |
| **N** | 26 | 26 | 26 | 26 | 26 | 26 | 26 | 26 |
| **geo Mean [CP]** | 23.13 | 23.63 | 22.07 | 25.57 | 26.46 | 24.22 | 24.17 | 27.10 |
| **ar Mean [CP]** | 23.14 | 23.64 | 22.08 | 25.57 | 26.46 | 24.22 | 24.18 | 27.10 |
| **min [CP]** | 22.68 | 22.45 | 21.41 | 24.87 | 25.81 | 23.57 | 23.26 | 26.39 |
| **max [CP]** | 24.15 | 24.42 | 23.12 | 26.42 | 27.14 | 25.14 | 25.33 | 27.94 |
| **std dev [± CP]** | 0.31 | 0.42 | 0.37 | 0.29 | 0.25 | 0.28 | 0.44 | 0.34 |
| **CV [% CP]** | 1.36 | 1.76 | 1.66 | 1.13 | 0.94 | 1.15 | 1.81 | 1.26 |
| **min [x-fold]** | -1.38 | -2.26 | -1.58 | -1.62 | -1.57 | -1.57 | -1.88 | -1.63 |
| **max [x-fold]** | 2.01 | 1.73 | 2.06 | 1.80 | 1.60 | 1.90 | 2.24 | 1.79 |
| **std dev [± x-fold]** | 1.24 | 1.33 | 1.29 | 1.22 | 1.19 | 1.21 | 1.35 | 1.27 |
| **Pearson correlation coefficient ( r )** | | | | | | | | |
| **vs.** | ***Act*** | ***Cyp*** | ***EF1a*** | ***EF1b*** | ***Fbox*** | ***TuB*** | ***TuA*** | ***60s*** |
| ***Cyp*** | 0.493 | - | - | - | - | - | - | - |
| **p-value** | 0.052 | - | - | - | - | - | - | - |
| ***EF1a*** | 0.923 | 0.396 | - | - | - | - | - | - |
| **p-value** | 0.001 | 0.130 | - | - | - | - | - | - |
| ***EF1b*** | 0.781 | 0.610 | 0.873 | - | - | - | - | - |
| **p-value** | 0.001 | 0.012 | 0.001 | - | - | - | - | - |
| ***Fbox*** | 0.610 | 0.493 | 0.511 | 0.540 | - | - | - | - |
| **p-value** | 0.012 | 0.052 | 0.043 | 0.031 | - | - | - | - |
| ***TuB*** | 0.871 | 0.538 | 0.867 | 0.760 | 0.524 | - | - | - |
| **p-value** | 0.001 | 0.031 | 0.001 | 0.001 | 0.037 | - | - | - |
| ***TuA*** | 0.838 | 0.204 | 0.874 | 0.614 | 0.307 | 0.868 | - | - |
| **p-value** | 0.001 | 0.448 | 0.001 | 0.011 | 0.246 | 0.001 | - | - |
| ***60s*** | 0.787 | 0.596 | 0.771 | 0.833 | 0.751 | 0.628 | 0.571 | - |
| **p-value** | 0.001 | 0.015 | 0.001 | 0.001 | 0.001 | 0.009 | 0.021 | - |
| **BestKeeper vs.** | ***Act*** | ***Cyp*** | ***EF1a*** | ***EF1b*** | ***Fbox*** | ***TuB*** | ***TuA*** | ***60s*** |
| **coeff. of corr. [r]** | 0.945 | 0.648 | 0.934 | 0.895 | 0.674 | 0.914 | 0.807 | 0.873 |
| **p-value** | 0.001 | 0.007 | 0.001 | 0.001 | 0.004 | 0.001 | 0.001 | 0.001 |
| **Regression Analysis: HKG vs. BestKeeper** | | | | | | | | |
|  | ***Act*** | ***Cyp*** | ***EF1a*** | ***EF1b*** | ***Fbox*** | ***TuB*** | ***TuA*** | ***60s*** |
| **coeff. of corr. [r]** | 0.95 | 0.65 | 0.93 | 0.90 | 0.67 | 0.91 | 0.81 | 0.87 |
| **coeff. of det. [r^2]** | 0.89 | 0.42 | 0.87 | 0.80 | 0.45 | 0.84 | 0.65 | 0.76 |
| **intercept [CP]** | -2.05 | 0.55 | -6.03 | 2.06 | 10.88 | 1.24 | -6.90 | 2.33 |
| **slope [CP]** | 1.03 | 0.94 | 1.15 | 0.96 | 0.64 | 0.94 | 1.27 | 1.01 |
| **SE [CP]** | ±0.135 | ±0.419 | ±0.166 | ±0.181 | ±0.264 | ±0.158 | ±0.351 | ±0.214 |
| **p-value** | 0.001 | 0.007 | 0.001 | 0.001 | 0.004 | 0.001 | 0.001 | 0.001 |
| **Power of HKG [x-fold]** | 2.04 | 1.92 | 2.22 | 1.95 | 1.55 | 1.92 | 2.41 | 2.02 |

**No.2** BestKeeper analysis of **candidate reference mRNA genes** for mRNA and miRNA precursors normalization in **root** tissue samples

| **CP data of housekeeping Genes** | | | | | | | | |
| --- | --- | --- | --- | --- | --- | --- | --- | --- |
|  | ***Act*** | ***Cyp*** | ***EF1a*** | ***EF1b*** | ***Fbox*** | ***TuB*** | ***TuA*** | ***60s*** |
| **N** | 26 | 26 | 26 | 26 | 26 | 26 | 26 | 26 |
| **geo Mean [CP]** | 21.80 | 22.86 | 20.57 | 23.27 | 25.07 | 23.11 | 23.66 | 26.08 |
| **ar Mean [CP]** | 21.81 | 22.87 | 20.58 | 23.28 | 25.08 | 23.12 | 23.66 | 26.08 |
| **min [CP]** | 20.97 | 21.95 | 19.89 | 22.54 | 24.28 | 22.06 | 22.66 | 25.03 |
| **max [CP]** | 22.41 | 23.53 | 21.41 | 24.01 | 25.82 | 24.35 | 24.17 | 26.94 |
| **std dev [± CP]** | 0.30 | 0.38 | 0.31 | 0.26 | 0.42 | 0.57 | 0.35 | 0.38 |
| **CV [% CP]** | 1.39 | 1.65 | 1.53 | 1.13 | 1.66 | 2.48 | 1.46 | 1.44 |
| **min [x-fold]** | -1.79 | -1.89 | -1.61 | -1.67 | -1.73 | -2.07 | -2.00 | -2.07 |
| **max [x-fold]** | 1.52 | 1.58 | 1.79 | 1.66 | 1.68 | 2.36 | 1.42 | 1.82 |
| **std dev [± x-fold]** | 1.23 | 1.30 | 1.24 | 1.20 | 1.34 | 1.49 | 1.27 | 1.30 |
| **Pearson correlation coefficient ( r )** | | | | | | | | |
| **vs.** | ***Act*** | ***Cyp*** | ***EF1a*** | ***EF1b*** | ***Fbox*** | ***TuB*** | ***TuA*** | ***60s*** |
| ***Cyp*** | 0.417 | - | - | - | - | - | - | - |
| **p-value** | 0.107 | - | - | - | - | - | - | - |
| ***EF1a*** | 0.447 | 0.881 | - | - | - | - | - | - |
| **p-value** | 0.083 | 0.001 | - | - | - | - | - | - |
| ***EF1b*** | 0.552 | 0.763 | 0.922 | - | - | - | - | - |
| **p-value** | 0.026 | 0.001 | 0.001 | - | - | - | - | - |
| ***Fbox*** | 0.564 | 0.837 | 0.784 | 0.804 | - | - | - | - |
| **p-value** | 0.023 | 0.000 | 0.001 | 0.001 | - | - | - | - |
| ***TuB*** | 0.840 | 0.385 | 0.267 | 0.353 | 0.455 | - | - | - |
| **p-value** | 0.001 | 0.141 | 0.316 | 0.180 | 0.077 | - | - | - |
| ***TuA*** | 0.600 | 0.512 | 0.634 | 0.651 | 0.408 | 0.589 | - | - |
| **p-value** | 0.014 | 0.043 | 0.008 | 0.006 | 0.117 | 0.016 | - | - |
| ***60s*** | 0.655 | 0.575 | 0.636 | 0.814 | 0.725 | 0.593 | 0.530 | - |
| **p-value** | 0.006 | 0.020 | 0.008 | 0.001 | 0.001 | 0.015 | 0.035 | - |
| **BestKeeper vs.** | ***Act*** | ***Cyp*** | ***EF1a*** | ***EF1b*** | ***Fbox*** | ***TuB*** | ***TuA*** | ***60s*** |
| **coeff. of corr. [r]** | 0.805 | 0.812 | 0.823 | 0.869 | 0.845 | 0.739 | 0.757 | 0.845 |
| **p-value** | 0.001 | 0.001 | 0.001 | 0.001 | 0.001 | 0.001 | 0.001 | 0.001 |
| **Regression Analysis: HKG vs. BestKeeper** | | | | | | | | |
|  | ***Act*** | ***Cyp*** | ***EF1a*** | ***EF1b*** | ***Fbox*** | ***TuB*** | ***TuA*** | ***60s*** |
| **coeff. of corr. [r]** | 0.81 | 0.81 | 0.82 | 0.87 | 0.85 | 0.74 | 0.76 | 0.85 |
| **coeff. of det. [r^2]** | 0.65 | 0.66 | 0.68 | 0.76 | 0.71 | 0.55 | 0.57 | 0.71 |
| **intercept [CP]** | 2.06 | -0.46 | -0.01 | 3.80 | 0.31 | -10.34 | 4.06 | 0.32 |
| **slope [CP]** | 0.85 | 1.00 | 0.89 | 0.84 | 1.07 | 1.44 | 0.84 | 1.11 |
| **SE [CP]** | ±0.247 | ±0.285 | ±0.241 | ±0.188 | ±0.266 | ±0.517 | ±0.287 | ±0.276 |
| **p-value** | 0.001 | 0.001 | 0.001 | 0.001 | 0.001 | 0.001 | 0.001 | 0.001 |
| **Power of HKG [x-fold]** | 1.80 | 2.00 | 1.85 | 1.79 | 2.09 | 2.71 | 1.79 | 2.16 |

**No.3** BestKeeper analysis of **candidate reference miRNA** for mature miRNA normalization in **leaf** tissue samples

| **CP data of housekeeping Genes** | | | | | | | | |
| --- | --- | --- | --- | --- | --- | --- | --- | --- |
|  | ***156a*** | ***166a*** | ***167a*** | ***171a*** | ***172a*** | ***393a*** | ***397a*** | ***1520d*** |
| **N** | 26 | 26 | 26 | 26 | 26 | 26 | 26 | 26 |
| **geo Mean [CP]** | 18.60 | 20.38 | 20.35 | 22.35 | 23.42 | 25.53 | 23.22 | 27.54 |
| **ar Mean [CP]** | 18.60 | 20.38 | 20.35 | 22.36 | 23.42 | 25.54 | 23.23 | 27.54 |
| **min [CP]** | 18.26 | 19.88 | 19.59 | 21.54 | 23.00 | 24.77 | 22.38 | 26.92 |
| **max [CP]** | 19.00 | 20.96 | 20.73 | 23.93 | 23.93 | 26.79 | 24.42 | 27.89 |
| **std dev [± CP]** | 0.15 | 0.25 | 0.22 | 0.55 | 0.28 | 0.53 | 0.44 | 0.20 |
| **CV [% CP]** | 0.82 | 1.22 | 1.07 | 2.44 | 1.19 | 2.07 | 1.90 | 0.72 |
| **min [x-fold]** | -1.27 | -1.42 | -1.70 | -1.75 | -1.34 | -1.70 | -1.79 | -1.54 |
| **max [x-fold]** | 1.32 | 1.49 | 1.30 | 2.99 | 1.43 | 2.40 | 2.30 | 1.27 |
| **std dev [± x-fold]** | 1.11 | 1.19 | 1.16 | 1.46 | 1.21 | 1.44 | 1.36 | 1.15 |
| **Pearson correlation coefficient ( r )** | | | | | | | | |
| **vs.** | ***156a*** | ***166a*** | ***167a*** | ***171a*** | ***172a*** | ***393a*** | ***397a*** | ***1520d*** |
| ***166a*** | 0.542 | - | - | - | - | - | - | - |
| **p-value** | 0.030 | - | - | - | - | - | - | - |
| ***167a*** | 0.577 | 0.849 | - | - | - | - | - | - |
| **p-value** | 0.019 | 0.001 | - | - | - | - | - | - |
| ***171a*** | 0.644 | 0.777 | 0.654 | - | - | - | - | - |
| **p-value** | 0.007 | 0.001 | 0.006 | - | - | - | - | - |
| ***172a*** | 0.426 | 0.879 | 0.707 | 0.774 | - | - | - | - |
| **p-value** | 0.100 | 0.000 | 0.002 | 0.001 | - | - | - | - |
| ***393a*** | 0.248 | 0.864 | 0.715 | 0.608 | 0.941 | - | - | - |
| **p-value** | 0.353 | 0.001 | 0.002 | 0.012 | 0.001 | - | - | - |
| ***397a*** | 0.220 | 0.189 | 0.326 | -0.134 | 0.000 | 0.079 | - | - |
| **p-value** | 0.415 | 0.483 | 0.218 | 0.618 | 1.000 | 0.769 | - | - |
| ***1520d*** | 0.698 | 0.466 | 0.413 | 0.833 | 0.456 | 0.276 | -0.095 | - |
| **p-value** | 0.003 | 0.069 | 0.111 | 0.001 | 0.075 | 0.303 | 0.724 | - |
| **BestKeeper vs.** | ***156a*** | ***166a*** | ***167a*** | ***171a*** | ***172a*** | ***393a*** | ***397a*** | ***1520d*** |
| **coeff. of corr. [r]** | 0.628 | 0.948 | 0.877 | 0.910 | 0.799 | 0.868 | 0.308 | 0.560 |
| **p-value** | 0.009 | 0.001 | 0.001 | 0.001 | 0.001 | 0.001 | 0.246 | 0.024 |
| **Regression Analysis: HKG vs. BestKeeper** | | | | | | | | |
|  | ***156a*** | ***166a*** | ***167a*** | ***171a*** | ***172a*** | ***393a*** | ***397a*** | ***1520d*** |
| **coeff. of corr. [r]** | 0.63 | 0.95 | 0.88 | 0.91 | 0.80 | 0.87 | 0.31 | 0.56 |
| **coeff. of det. [r^2]** | 0.39 | 0.90 | 0.77 | 0.83 | 0.64 | 0.75 | 0.10 | 0.31 |
| **intercept [CP]** | 9.20 | -1.15 | 0.96 | -24.43 | 4.47 | -16.86 | 10.47 | 16.70 |
| **slope [CP]** | 0.42 | 0.96 | 0.86 | 2.08 | 0.84 | 1.88 | 0.57 | 0.48 |
| **SE [CP]** | ±0.162 | ±0.1 | ±0.147 | ±0.296 | ±0.198 | ±0.336 | ±0.547 | ±0.222 |
| **p-value** | 0.009 | 0.001 | 0.001 | 0.001 | 0.001 | 0.001 | 0.246 | 0.024 |
| **Power of HKG [x-fold]** | 1.34 | 1.94 | 1.82 | 4.22 | 1.79 | 3.69 | 1.48 | 1.40 |

**No.4** BestKeeper analysis of **candidate reference miRNA** for mature miRNA normalization in **root** tissue samples

| **CP data of housekeeping Genes** | | | | | | | | |
| --- | --- | --- | --- | --- | --- | --- | --- | --- |
|  | ***156a*** | ***166a*** | ***167a*** | ***171a*** | ***172a*** | ***393a*** | ***397a*** | ***1520d*** |
| **N** | 26 | 26 | 26 | 26 | 26 | 26 | 26 | 26 |
| **geo Mean [CP]** | 24.55 | 21.07 | 24.15 | 29.23 | 21.04 | 28.02 | 21.90 | 26.74 |
| **ar Mean [CP]** | 24.57 | 21.07 | 24.15 | 29.23 | 21.04 | 28.03 | 21.91 | 26.75 |
| **min [CP]** | 23.62 | 20.57 | 23.58 | 28.25 | 20.56 | 27.28 | 21.26 | 25.37 |
| **max [CP]** | 26.92 | 22.31 | 25.32 | 30.08 | 21.79 | 29.74 | 22.65 | 27.48 |
| **std dev [± CP]** | 0.71 | 0.48 | 0.28 | 0.31 | 0.27 | 0.36 | 0.36 | 0.36 |
| **CV [% CP]** | 2.88 | 1.73 | 1.16 | 1.06 | 1.29 | 1.71 | 1.66 | 1.36 |
| **min [x-fold]** | -1.91 | -1.42 | -1.48 | -1.97 | -1.40 | -1.67 | -1.56 | -2.59 |
| **max [x-fold]** | 5.14 | 2.36 | 2.26 | 1.80 | 1.68 | 3.30 | 1.68 | 1.66 |
| **std dev [± x-fold]** | 1.63 | 1.29 | 1.22 | 1.24 | 1.21 | 1.39 | 1.29 | 1.29 |
| **Pearson correlation coefficient ( r )** | | | | | | | | |
| **vs.** | ***156a*** | ***166a*** | ***167a*** | ***171a*** | ***172a*** | ***393a*** | ***397a*** | ***1520d*** |
| ***166a*** | 0.709 | - | - | - | - | - | - | - |
| **p-value** | 0.002 | - | - | - | - | - | - | - |
| ***167a*** | 0.901 | 0.864 | - | - | - | - | - | - |
| **p-value** | 0.001 | 0.001 | - | - | - | - | - | - |
| ***171a*** | 0.674 | 0.830 | 0.766 | - | - | - | - | - |
| **p-value** | 0.004 | 0.001 | 0.001 | - | - | - | - | - |
| ***172a*** | 0.560 | 0.802 | 0.738 | 0.874 | - | - | - | - |
| **p-value** | 0.024 | 0.000 | 0.001 | 0.001 | - | - | - | - |
| ***393a*** | 0.928 | 0.736 | 0.841 | 0.706 | 0.546 | - | - | - |
| **p-value** | 0.001 | 0.001 | 0.001 | 0.002 | 0.029 | - | - | - |
| ***397a*** | 0.456 | 0.406 | 0.580 | 0.539 | 0.545 | 0.440 | - | - |
| **p-value** | 0.075 | 0.119 | 0.019 | 0.031 | 0.029 | 0.089 | - | - |
| ***1520d*** | 0.449 | 0.476 | 0.592 | 0.571 | 0.657 | 0.488 | 0.759 | - |
| **p-value** | 0.081 | 0.062 | 0.016 | 0.021 | 0.006 | 0.055 | 0.001 | - |
| **BestKeeper vs.** | ***156a*** | ***166a*** | ***167a*** | ***171a*** | ***172a*** | ***393a*** | ***397a*** | ***1520d*** |
| **coeff. of corr. [r]** | 0.889 | 0.883 | 0.951 | 0.868 | 0.817 | 0.863 | 0.684 | 0.716 |
| **p-value** | 0.001 | 0.001 | 0.001 | 0.001 | 0.001 | 0.001 | 0.003 | 0.002 |
| **Regression Analysis: HKG vs. BestKeeper** | | | | | | | | |
|  | ***156a*** | ***166a*** | ***167a*** | ***171a*** | ***172a*** | ***393a*** | ***397a*** | ***1520d*** |
| **coeff. of corr. [r]** | 0.89 | 0.88 | 0.95 | 0.87 | 0.82 | 0.86 | 0.68 | 0.72 |
| **coeff. of det. [r^2]** | 0.79 | 0.75 | 0.90 | 0.75 | 0.67 | 0.78 | 0.47 | 0.51 |
| **intercept [CP]** | -21.24 | -1.97 | 1.94 | 8.11 | 5.70 | -3.94 | 4.94 | 6.29 |
| **slope [CP]** | 1.88 | 0.94 | 0.91 | 0.86 | 0.63 | 1.31 | 0.69 | 0.84 |
| **SE [CP]** | ±0.426 | ±0.243 | ±0.131 | ±0.218 | ±0.196 | ±0.306 | ±0.326 | ±0.36 |
| **p-value** | 0.001 | 0.001 | 0.001 | 0.001 | 0.001 | 0.001 | 0.003 | 0.002 |
| **Power of HKG [x-fold]** | 3.67 | 1.92 | 1.88 | 1.82 | 1.55 | 2.48 | 1.62 | 1.79 |
