# Supplementary material for: Normalization for Relative Quantification of mRNA and microRNA in Soybean Exposed to Various Abiotic Stresses
Source: PLoS One. 2016 May 13;11(5):e0155606. doi: 10.1371/journal.pone.0155606 (PMC4866712; doi:10.1371/journal.pone.0155606)
Supplement: S1 Table — (DOC) [file pone.0155606.s006.doc]

**Table S1 Description of the samples used for RT-qPCR.**

| **Sample No.** | **Cultivar** | **Developmental stage** | **Abiotic Stress Conditions** | **Treated time** | **Tissue** | **The number of technical replicates** | **The number of biological replicates** |
| --- | --- | --- | --- | --- | --- | --- | --- |
| 1 | Williams 82 | 1^st^ unifoliolate leaves | Drought (8% PEG8000) | 1 hours | Leaves | 3 | 3 |
| 2 | Williams 82 | 1^st^ unifoliolate leaves | Drought (8% PEG8000) | 3 hours | Leaves | 3 | 3 |
| 3 | Williams 82 | 1^st^ unifoliolate leaves | Drought (8% PEG8000) | 6 hours | Leaves | 3 | 3 |
| 4 | Williams 82 | 1^st^ unifoliolate leaves | Drought (8% PEG8000) | 9 hours | Leaves | 3 | 3 |
| 5 | Williams 82 | 1^st^ unifoliolate leaves | Drought (8% PEG8000) | 12 hours | Leaves | 3 | 3 |
| 6 | Williams 82 | 1^st^ unifoliolate leaves | Drought (8% PEG8000) | 1 hours | Roots | 3 | 3 |
| 7 | Williams 82 | 1^st^ unifoliolate leaves | Drought (8% PEG8000) | 3 hours | Roots | 3 | 3 |
| 8 | Williams 82 | 1^st^ unifoliolate leaves | Drought (8% PEG8000) | 6 hours | Roots | 3 | 3 |
| 9 | Williams 82 | 1^st^ unifoliolate leaves | Drought (8% PEG8000) | 9 hours | Roots | 3 | 3 |
| 10 | Williams 82 | 1^st^ unifoliolate leaves | Drought (8% PEG8000) | 12 hours | Roots | 3 | 3 |
| 11 | Williams 82 | 1^st^ unifoliolate leaves | Salinity (120 mM NaCl) | 1 hours | Leaves | 3 | 3 |
| 12 | Williams 82 | 1^st^ unifoliolate leaves | Salinity (120 mM NaCl) | 3 hours | Leaves | 3 | 3 |
| 13 | Williams 82 | 1^st^ unifoliolate leaves | Salinity (120 mM NaCl) | 6 hours | Leaves | 3 | 3 |
| 14 | Williams 82 | 1^st^ unifoliolate leaves | Salinity (120 mM NaCl) | 9 hours | Leaves | 3 | 3 |
| 15 | Williams 82 | 1^st^ unifoliolate leaves | Salinity (120 mM NaCl) | 12 hours | Leaves | 3 | 3 |
| 16 | Williams 82 | 1^st^ unifoliolate leaves | Salinity (120 mM NaCl) | 1 hours | Roots | 3 | 3 |
| 17 | Williams 82 | 1^st^ unifoliolate leaves | Salinity (120 mM NaCl) | 3 hours | Roots | 3 | 3 |
| 18 | Williams 82 | 1^st^ unifoliolate leaves | Salinity (120 mM NaCl) | 6 hours | Roots | 3 | 3 |
| 19 | Williams 82 | 1^st^ unifoliolate leaves | Salinity (120 mM NaCl) | 9 hours | Roots | 3 | 3 |
| 20 | Williams 82 | 1^st^ unifoliolate leaves | Salinity (120 mM NaCl) | 12 hours | Roots | 3 | 3 |
| 21 | Williams 82 | 1^st^ unifoliolate leaves | Aalkalinity (100 mM NaHCO_3_) | 1 hours | Leaves | 3 | 3 |
| 22 | Williams 82 | 1^st^ unifoliolate leaves | Aalkalinity (100 mM NaHCO_3_) | 3 hours | Leaves | 3 | 3 |
| 23 | Williams 82 | 1^st^ unifoliolate leaves | Aalkalinity (100 mM NaHCO_3_) | 6 hours | Leaves | 3 | 3 |
| 24 | Williams 82 | 1^st^ unifoliolate leaves | Aalkalinity (100 mM NaHCO_3_) | 9 hours | Leaves | 3 | 3 |
| 25 | Williams 82 | 1^st^ unifoliolate leaves | Aalkalinity (100 mM NaHCO_3_) | 12 hours | Leaves | 3 | 3 |
| 26 | Williams 82 | 1^st^ unifoliolate leaves | Aalkalinity (100 mM NaHCO_3_) | 1 hours | Roots | 3 | 3 |
| 27 | Williams 82 | 1^st^ unifoliolate leaves | Aalkalinity (100 mM NaHCO_3_) | 3 hours | Roots | 3 | 3 |
| 28 | Williams 82 | 1^st^ unifoliolate leaves | Aalkalinity (100 mM NaHCO_3_) | 6 hours | Roots | 3 | 3 |
| 29 | Williams 82 | 1^st^ unifoliolate leaves | Aalkalinity (100 mM NaHCO_3_) | 9 hours | Roots | 3 | 3 |
| 30 | Williams 82 | 1^st^ unifoliolate leaves | Aalkalinity (100 mM NaHCO_3_) | 12 hours | Roots | 3 | 3 |
| 31 | Williams 82 | 1^st^ unifoliolate leaves | Salinity and alkalinity  (70 mM NaCl and 50 mM NaHCO_3_) | 1 hours | Leaves | 3 | 3 |
| 32 | Williams 82 | 1^st^ unifoliolate leaves | Salinity and alkalinity  (70 mM NaCl and 50 mM NaHCO_3_) | 3 hours | Leaves | 3 | 3 |
| 33 | Williams 82 | 1^st^ unifoliolate leaves | Salinity and alkalinity  (70 mM NaCl and 50 mM NaHCO_3_) | 6 hours | Leaves | 3 | 3 |
| 34 | Williams 82 | 1^st^ unifoliolate leaves | Salinity and alkalinity  (70 mM NaCl and 50 mM NaHCO_3_) | 9 hours | Leaves | 3 | 3 |
| 35 | Williams 82 | 1^st^ unifoliolate leaves | Salinity and alkalinity  (70 mM NaCl and 50 mM NaHCO_3_) | 12 hours | Leaves | 3 | 3 |
| 36 | Williams 82 | 1^st^ unifoliolate leaves | Salinity and alkalinity  (70 mM NaCl and 50 mM NaHCO_3_) | 1 hours | Roots | 3 | 3 |
| 37 | Williams 82 | 1^st^ unifoliolate leaves | Salinity and alkalinity  (70 mM NaCl and 50 mM NaHCO_3_) | 3 hours | Roots | 3 | 3 |
| 38 | Williams 82 | 1^st^ unifoliolate leaves | Salinity and alkalinity  (70 mM NaCl and 50 mM NaHCO_3_) | 6 hours | Roots | 3 | 3 |
| 39 | Williams 82 | 1^st^ unifoliolate leaves | Salinity and alkalinity  (70 mM NaCl and 50 mM NaHCO_3_) | 9 hours | Roots | 3 | 3 |
| 40 | Williams 82 | 1^st^ unifoliolate leaves | Salinity and alkalinity  (70 mM NaCl and 50 mM NaHCO_3_) | 12 hours | Roots | 3 | 3 |
| 41 | Williams 82 | 1^st^ unifoliolate leaves | Abscisic Acid (200uM ABA ) | 1 hours | Leaves | 3 | 3 |
| 42 | Williams 82 | 1^st^ unifoliolate leaves | Abscisic Acid (200uM ABA ) | 3 hours | Leaves | 3 | 3 |
| 43 | Williams 82 | 1^st^ unifoliolate leaves | Abscisic Acid (200uM ABA ) | 6 hours | Leaves | 3 | 3 |
| 44 | Williams 82 | 1^st^ unifoliolate leaves | Abscisic Acid (200uM ABA ) | 9 hours | Leaves | 3 | 3 |
| 45 | Williams 82 | 1^st^ unifoliolate leaves | Abscisic Acid (200uM ABA ) | 12 hours | Leaves | 3 | 3 |
| 46 | Williams 82 | 1^st^ unifoliolate leaves | Abscisic Acid (200uM ABA ) | 1 hours | Roots | 3 | 3 |
| 47 | Williams 82 | 1^st^ unifoliolate leaves | Abscisic Acid (200uM ABA ) | 3 hours | Roots | 3 | 3 |
| 48 | Williams 82 | 1^st^ unifoliolate leaves | Abscisic Acid (200uM ABA ) | 6 hours | Roots | 3 | 3 |
| 49 | Williams 82 | 1^st^ unifoliolate leaves | Abscisic Acid (200uM ABA ) | 9 hours | Roots | 3 | 3 |
| 50 | Williams 82 | 1^st^ unifoliolate leaves | Abscisic Acid (200uM ABA ) | 12 hours | Roots | 3 | 3 |
| 51 | Williams 82 | 1^st^ unifoliolate leaves | Blank control (No treatment) | 0 hours | Leaves | 3 | 3 |
| 52 | Williams 82 | 1^st^ unifoliolate leaves | Blank control (No treatment) | 0 hours | Roots | 3 | 3 |

Note: 1. Three biological replicates have been pooled together after cDNA synthesis for expression stability assessment of candidate reference mRNA/miRNA.

2. The sets of samples. (1) Leaf and root combined samples (n = 52). (2) Leaf samples (n = 26). (3)Root samples (n = 26).
